# Genome-wide Association Analysis in Humans Links Nucleotide Metabolism to Leukocyte Telomere Length

**DOI:** 10.1016/j.ajhg.2020.02.006

**Published:** 2020-02-27

**Authors:** Chen Li, Svetlana Stoma, Luca A. Lotta, Sophie Warner, Eva Albrecht, Alessandra Allione, Pascal P. Arp, Linda Broer, Jessica L. Buxton, Alexessander Da Silva Couto Alves, Joris Deelen, Iryna O. Fedko, Scott D. Gordon, Tao Jiang, Robert Karlsson, Nicola Kerrison, Taylor K. Loe, Massimo Mangino, Yuri Milaneschi, Benjamin Miraglio, Natalia Pervjakova, Alessia Russo, Ida Surakka, Ashley van der Spek, Josine E. Verhoeven, Najaf Amin, Marian Beekman, Alexandra I. Blakemore, Federico Canzian, Stephen E. Hamby, Jouke-Jan Hottenga, Peter D. Jones, Pekka Jousilahti, Reedik Mägi, Sarah E. Medland, Grant W. Montgomery, Dale R. Nyholt, Markus Perola, Kirsi H. Pietiläinen, Veikko Salomaa, Elina Sillanpää, H. Eka Suchiman, Diana van Heemst, Gonneke Willemsen, Antonio Agudo, Heiner Boeing, Dorret I. Boomsma, Maria-Dolores Chirlaque, Guy Fagherazzi, Pietro Ferrari, Paul Franks, Christian Gieger, Johan Gunnar Eriksson, Marc Gunter, Sara Hägg, Iiris Hovatta, Liher Imaz, Jaakko Kaprio, Rudolf Kaaks, Timothy Key, Vittorio Krogh, Nicholas G. Martin, Olle Melander, Andres Metspalu, Concha Moreno, N. Charlotte Onland-Moret, Peter Nilsson, Ken K. Ong, Kim Overvad, Domenico Palli, Salvatore Panico, Nancy L. Pedersen, Brenda W.J. H. Penninx, J. Ramón Quirós, Marjo Riitta Jarvelin, Miguel Rodríguez-Barranco, Robert A. Scott, Gianluca Severi, P. Eline Slagboom, Tim D. Spector, Anne Tjonneland, Antonia Trichopoulou, Rosario Tumino, André G. Uitterlinden, Yvonne T. van der Schouw, Cornelia M. van Duijn, Elisabete Weiderpass, Eros Lazzerini Denchi, Giuseppe Matullo, Adam S. Butterworth, John Danesh, Nilesh J. Samani, Nicholas J. Wareham, Christopher P. Nelson, Claudia Langenberg, Veryan Codd

**Affiliations:** 1 MRC Epidemiology Unit, University of Cambridge, CB2 0SL, United Kingdom; 2 Department of Cardiovascular Sciences, University of Leicester, LE3 9QP, United Kingdom; 3 NIHR Leicester Biomedical Research Centre, Glenfield Hospital, Leicester, LE3 9QP, United Kingdom; 4 Institute of Epidemiology, Helmholtz Zentrum München—German Research Centre for Environmental Health, D-85764 Neuherberg, Germany; 5 Department of Medical Science, Genomic Variation and Translational Research Unit, University of Turin, 10126 Turin, Italy; 6 Italian Institute for Genomic Medicine (IIGM), 10126 Turin, Italy; 7 Department of Internal Medicine, Erasmus Medical Centre, Postbus 2040, 3000 CA, Rotterdam, the Netherlands; 8 School of Life Sciences, Pharmacy, and Chemistry, Kingston University, Kingston upon Thames, KT1 2EE, United Kingdom; 9 Genetics and Genomic Medicine Programme, UCL Great Ormond Street Institute of Child Health, London, WC1N 1EH, United Kingdom; 10 School of Public Health, Imperial College London, St Mary’s Hospital, London W2 1PG, United Kingdom; 11 School of Biosciences and Medicine, University of Surrey, Guildford, GU2 7XH, United Kingdom; 12 Max Planck Institute for Biology of Ageing, D-50931, Cologne, Germany; 13 Department of Biomedical Data Sciences, Section of Molecular Epidemiology, Leiden University Medical Centre, PO Box 9600, 2300 RC, Leiden, the Netherlands; 14 Department of Biological Psychology, Vrije Universteit, 1081 BT Amsterdam, the Netherlands; 15 Genetic Epidemiology, QIMR Berghofer Medical Research Institute, Queensland, 4006 Australia; 16 BHF Cardiovascular Epidemiology Unit, Department of Public Health and Primary Care, University of Cambridge, CB1 8RN, United Kingdom; 17 Department of Medical Epidemiology and Biostatistics, Karolinska Institutet, Stockholm 17177, Sweden; 18 Department of Molecular Medicine, The Scripps Research Institute, La Jolla, CA 92037, USA; 19 Department of Twin Research and Genetic Epidemiology, Kings College London, London SE1 7EH, United Kingdom; 20 NIHR Biomedical Research Centre at Guy’s and St Thomas’ Foundation Trust, London SE1 9RT, United Kingdom; 21 Department of Psychiatry, Amsterdam Public Health and Amsterdam Neuroscience, Amsterdam UMC/Vrije Universiteit, 1081HJ, Amsterdam, the Netherlands; 22 Institute for Molecular Medicine Finland (FIMM), PO Box 20, 00014 University of Helsinki, Finland; 23 Estonian Genome Centre, Institute of Genomics, University of Tartu, 51010, Tartu, Estonia; 24 Division of Cardiovascular Medicine, Department of Internal Medicine, University of Michigan, Ann Arbor, MI 48109, USA; 25 Department of Epidemiology, Erasmus Medical Centre, Postbus 2040, 3000 CA, Rotterdam, the Netherlands; 26 Department of Life Sciences, Brunel University London, Uxbridge UB8 3PH, United Kingdom; 27 Department of Medicine, Imperial College London, London, W12 0HS, United Kingdom; 28 Genomic Epidemiology Group, German Cancer Research Centre (DKFZ), 69120 Heidelberg, Germany; 29 Department of Public Health Solutions, Finnish Institute for Health and Welfare, PO Box 30, FI-00271 Helsinki, Finland; 30 Institute for Molecular Bioscience, The University of Queensland, 4072, Queensland, Australia; 31 School of Biomedical Sciences and Institute of Health and Biomedical Innovation, Queensland University of Technology, Queensland, 4059, Australia; 32 Research Program for Clinical and Molecular Metabolism, Faculty of Medicine, Biomedicum 1, PO Box 63, 00014 University of Helsinki, Finland; 33 Obesity Research Unit, Research Program for Clinical and Molecular Metabolism, Haartmaninkatu 8, 00014 University of Helsinki, Helsinki, Finland; 34 Obesity Center, Abdominal Center, Endocrinology, Helsinki University Hospital and University of Helsinki, Haartmaninkatu 4, 00029 HUS, Helsinki, Finland; 35 Gerontology Research Center, Faculty of Sport and Health Sciences, PO Box 35, 40014 University of Jyväskylä, Finland; 36 Department of Internal Medicine, Section of Gerontology and Geriatrics, Leiden University Medical Centre, PO Box 9600, 2300 RC, Leiden, the Netherlands; 37 Unit of Nutrition, Environment, and Cancer, Cancer Epidemiology Research Program, Catalan Institute of Oncology—ICO, Group of Research on Nutrition and Cancer, Bellvitge Biomedical Research Institute—IDIBELL, L’Hospitalet of Llobregat, 08908 Barcelona, Spain; 38 German Institute of Human Nutrition Potsdam—Rehbruecke, 14558 Nuthetal, Germany; 39 Department of Epidemiology, Murcia Regional Health Council, IMIB—Arrixaca, 30008, Murcia, Spain; 40 CIBER of Epidemiology and Public Health (CIBERESP), 28029 Madrid, Spain; 41 Center of Research in Epidemiology and Population Health, UMR 1018 Inserm, Institut Gustave Roussy, Paris-Sud Paris-Saclay University, 94805 Villejuif, France; 42 Digital Epidemiology Research Hub, Department of Population Health, Luxembourg Institute of Health, L-1445 Strassen, Luxembourg; 43 International Agency for Research on Cancer, 69372 Lyon, France; 44 Department of Clinical Sciences, Clinical Research Center, Skåne University Hospital, Lund University, 20502 Malmö, Sweden; 45 Department of Public Health and Clinical Medicine, Umeå University, 90187 Umeå, Sweden; 46 Research Unit of Molecular Epidemiology, Helmholtz Zentrum München, German Research Center for Environmental Health, D 85764 Neuherberg, Germany; 47 German Center for Diabetes Research (DZD e.V.), D-85764 Neuherberg, Germany; 48 Department of General Practice and Primary Health Care, University of Helsinki and Helsinki University Hospital, PO Box 20, 00014 University of Helsinki, Finland; 49 Folkhälsan Research Centre, PO Box 20, 00014 University of Helsinki, Finland; 50 Obstetrics and Gynaecology, Yong Loo Lin School of Medicine, National University of Singapore, Singapore 117597; 51 SleepWell Research Program, Haartmaninkatu 3, 00014 University of Helsinki, Finland; 52 Department of Psychology and Logopedics, Haartmaninkatu 3, 00014 University of Helsinki, Finland; 53 Ministry of Health of the Basque Government, Public Health Division of Gipuzkoa, 20013 Donostia-San Sebastian, Spain; 54 Biodonostia Health Research Institute, 20014 Donostia-San Sebastian, Spain; 55 Department of Public Health, PO Box 20, 00014 University of Helsinki, Finland; 56 Division of Cancer Epidemiology, German Cancer Research Center (DKFZ), 69120 Heidelberg, Germany; 57 Cancer Epidemiology Unit, Nuffield Department of Population Health, University of Oxford, OX3 7LF, United Kingdom; 58 Epidemiology and Prevention Unit, Fondazione IRCCS—Istituto Nazionale dei Tumori, 20133 Milan, Italy; 59 Department of Clinical Sciences, Hypertension, and Cardiovascular Disease, Lund University, 21428 Malmö, Sweden; 60 Instituto de Salud Pública, 31003 Pamplona, Spain; 61 Julius Center for Health Sciences and Primary Care, University Medical Center Utrecht, Utrecht University, 3584 CG Utrecht, the Netherlands; 62 Department of Paediatrics, University of Cambridge, CB2 0QQ, United Kingdom; 63 Department of Public Health, Aarhus University, DK-8000 Aarhus, Denmark; 64 Department of Cardiology, Aalborg University Hospital, DK-9000 Aalborg, Denmark; 65 Cancer Risk Factors and Life-Style Epidemiology Unit, Institute for Cancer Research—ISPRO, 50139 Florence, Italy; 66 Dipartimento di Medicina Clinica e Chirurgia, Federico II University, 80131 Naples, Italy; 67 Consejería de Sanidad, Public Health Directorate, 33006 Asturias, Spain; 68 School of Epidemiology and Biostatistics, Imperial College London, SW7 2AZ, United Kingdom; 69 Andalusian School of Public Health (EASP), 18080 Granada, Spain; 70 Instituto de Investigación Biosanitaria ibs.GRANADA, 18012 Granada, Spain; 71 CESP, Facultés de médecine, Université Paris, 94805 Villejuif, France; 72 Gustave Roussy, 94805 Villejuif, France; 73 Department of Statistics, Computer Science, Applications “G. Parenti,” University of Florence, 50134 Firenze, Italy; 74 Danish Cancer Society Research Center, 2100 Copenhagen, Denmark; 75 Hellenic Health Foundation, 11527 Athens, Greece; 76 Cancer Registry and Histopathology Department, Provincial Health Authority (ASP), 97100 Ragusa, Italy; 77 Hyblean Association for Research on Epidemiology, No Profit Organization, 97100 Ragusa, Italy; 78 Nuffield Department of Population Health, University of Oxford, OX3 7LF, United Kingdom; 79 Laboratory of Chromosome Instability, National Cancer Institute, NIH, Bethesda, MD 20892 USA; 80 Health Data Research UK Cambridge, Wellcome Genome Campus and University of Cambridge, CB10 1SA, United Kingdom; 81 NIHR Blood and Transplant Research Unit in Donor Health and Genomics, Department of Public Health and Primary Care, University of Cambridge, CB1 8RN, United Kingdom; 82 Department of Human Genetics, Wellcome Sanger Institute, Hinxton, CB10 1SA, United Kingdom; 83 BHF Cambridge Centre of Excellence, School of Clinical Medicine, Addenbrookes’ Hospital, Cambridge, CB2 0QQ, United Kingdom; 84 NIHR Cambridge Biomedical Research Centre, School of Clinical Medicine, Addenbrooke’s Hospital, Cambridge CB2 0QQ, United Kingdom

**Keywords:** telomere length, biological aging, Mendelian randomisation, age-related disease

## Abstract

Leukocyte telomere length (LTL) is a heritable biomarker of genomic aging. In this study, we perform a genome-wide meta-analysis of LTL by pooling densely genotyped and imputed association results across large-scale European-descent studies including up to 78,592 individuals. We identify 49 genomic regions at a false dicovery rate (FDR) < 0.05 threshold and prioritize genes at 31, with five highlighting nucleotide metabolism as an important regulator of LTL. We report six genome-wide significant loci in or near *SENP7*, *MOB1B*, *CARMIL1*, *PRRC2A*, *TERF2,* and *RFWD3*, and our results support recently identified *PARP1, POT1*, *ATM,* and *MPHOSPH6* loci. Phenome-wide analyses in >350,000 UK Biobank participants suggest that genetically shorter telomere length increases the risk of hypothyroidism and decreases the risk of thyroid cancer, lymphoma, and a range of proliferative conditions. Our results replicate previously reported associations with increased risk of coronary artery disease and lower risk for multiple cancer types. Our findings substantially expand current knowledge on genes that regulate LTL and their impact on human health and disease.

## Introduction

Telomeres are DNA-protein complexes found at the ends of eukaryotic chromosomes, and they serve to maintain genomic stability and determine cellular lifespan.^1^ Telomere length (TL) declines with cellular divisions; this is due to the inability of DNA polymerase to fully replicate the 3′ end of the DNA strand (the “end replication problem”), and once a critically short TL is reached, the cell enters replicative senescence.^2^ Protein complexes, including the SHELTERIN complexes—which are comprised of TERF1 (MIM: 600951), TERF2 (MIM: 602027), POT1 (MIM: 606478), TERF2IP (MIM: 605061), TINF2 (MIM: 604319), ACD (MIM: 609377), and CST (CTC1 [MIM: 613129], STN1 [MIM: 613128], and TEN1 [MIM: 613130])—along with DNA helicases such as RTEL1 (MIM: 608833), bind telomeres and regulate TL and structure.^3^ In some cell types, such as stem and germline progenitor cells, TL is maintained by the enzyme telomerase, a ribonucleoprotein containing the RNA template TERC (MIM: 602322), a reverse transcriptase (TERT [MIM: 187270]), and accessory proteins (DKC1 [MIM: 300126], NOP10 [MIM: 606471], GAR1 [MIM: 606468], and NHP2 [MIM: 606470]).^4^

Severe telomere loss, through loss-of-function mutations of core telomere and telomerase components, leads to several diseases which share features such as bone marrow failure and organ damage. These “telomere syndromes” include dyskeratosis congenita (MIM: 305000), aplastic anemia (MIM: 609135), and idiopathic pulmonary fibrosis (MIM:614742) among others.^5^^,^^6^ While the prevalence of such syndromes varies, they are all relatively rare. One feature of these syndromes is premature aging.^5^ Along with shorter TL observed at older ages in cross sectional population studies, this has led to TL (most commonly measured in human leukocytes as leucocyte telomere length [LTL]) to be proposed as a marker of biological age. LTL has been shown to be associated with the risk of common age-related diseases, including coronary artery disease (CAD) and some cancers.^7^, ^8^, ^9^, ^10^, ^11^, ^12^ However, whether LTL (reflecting TL across tissues) was causally associated with disease or whether the observed associations may have been due to reverse causation or confounding was unclear.

LTL is both variable among individuals, from birth and throughout the life course, and highly heritable, with heritability estimates from 44%–86%.^13^^,^^14^ Identification of genetic determinants of LTL through a genome-wide association study (GWAS) has allowed further studies to suggest a causal role for LTL in several diseases, including CAD, abdominal aortic aneurysm, several cancers, interstitial lung disease, and celiac disease.^15^, ^16^, ^17^, ^18^, ^19^ However, these studies are limited due to the small number of genetic variants that have been identified that replicate between studies.^15^^,^^20^, ^21^, ^22^, ^23^, ^24^, ^25^ To further our understanding of LTL regulation and its relationship with disease, we have conducted a genome-wide association (GWA) meta-analysis of 78,592 individuals from the European Network for Genetic and Genomic Epidemiology (ENGAGE) study and from the European Prospective Investigation into Cancer and Nutrition (EPIC) Cardiovascular Disease (CVD) and InterAct studies.

## Subjects and Methods

Full descriptions of the EPIC-CVD and EPIC-InterAct cohorts, along with the participating cohorts within the ENGAGE consortium, are given in the Supplemental Information.

### LTL Measurements and QC Analysis

Mean LTL measurements were conducted using an established quantitative PCR technique which expressed TL as a ratio of the telomere repeat number (T) to a single-copy gene (S).^26^^,^^27^ The majority of the ENGAGE samples were included within our previous analysis.^15^ LTL measurements were standardized either by using a calibrator sample or by quantifying against a standard curve, depending on the laboratory (Table S1 and Supplemental Methods). Full details of the methodology employed by each laboratory, along with quality control (QC) parameters, is given in the Supplemental Information or is given in detail elsewhere.^15^ Because the use of different calibrator samples or of standard curves for quantification can lead to different ranges in the *T/S* ratios being observed between laboratories, we standardized LTL by using a z-transformation approach (z = (μ - μ_0_)/σ, μ, *T/S* ratio, μ_0_, the mean *T/S* ratio, σ, standard deviation [SD]).

### Genotyping, GWAS Analysis, and Study-Level QC

Genotyping platforms and imputation methods and panels varied across participating study centers. Detailed information about these is provided in Figure S1 and Table S2. A GWAS was run within each study through the use of linear regression under an additive mode of inheritance with adjustment for age, sex, and any study-specific covariates, including batch, center, and genetic principle components. There are 21 studies contributing to ENGAGE. For the EPIC InterAct and CVD studies, association analyses were stratified based on genotyping platform and disease status, resulting in nine strata. Within each study or stratum, related samples (k > 0.088) were removed. Population stratification was estimated using the genomic control inflation factor λ and used to adjust the standard errors. Genetic variants were filtered on the basis of the published standards that included call rate >95%, Hardy–Weinberg equilibrium p < 1 × 10^−6^, imputation quality info-score >0.4 or R^2^ > 0.3, minor allele count ≧10, and standard error of association estimates ranging from 0 to 10.^15^^,^^28^^,^^29^ These data were taken forward to the meta-analysis.

### Meta-analyses

GWAS summary statistics were combined via two steps of meta-analyses by using inverse variance weighting in GWAMA.^30^ We first combined all 21 ENGAGE studies together and separately combined the nine EPIC-InterAct and EPIC-CVD strata, where a genetic variant was retained if it had >40% of the available sample size within these two cohorts. Fixed effects were used except for variants with significant heterogeneity (Cochrane’s Q: p < 1 × 10^−6^), in which case random effects were used. Additional adjustment was made for genomic inflation (see Figure S2). In the second step, association estimates derived from the two separate meta-analyses estimated in the first step were combined using fixed effects inverse variance weighted meta-analyses. We estimated the FDR by estimating q-values^31^ for these data.

### Conditional Association Analysis

Conditionally independent signals were identified via an approximate genome-wide stepwise method, using GCTA (Version 1.25.2),^32^^,^^33^ that allows for conditional analyses to be run on summary statistics without individual-level data. Summary statistics from the final meta-analysis were used as the input, with p value cut-offs at 5 × 10^−8^ (genome-wide significance) or 1.03 × 10^−5^ (equivalent to an FDR < 0.05). The model starts with the most significant SNP, adds in SNPs iteratively in a forward stepwise manner, and calculates conditional p values for all SNPs within the model. If the target SNP shows evidence of collinearity (correlation coefficients r^2^ > 0.9, with linkage disequilibrium (LD) estimated based on a random subcohort of 50,000 UK Biobank samples) with any of the SNPs selected into the model, the conditional p value of the target SNP was set to 1. The selection process was repeated until no more SNPs could be fitted into the model, i.e., there were no more SNPs that could reach the conditional p value thresholds (5 × 10^−8^ or 1.03 × 10^−5^, corresponding to the p value cut-offs in the input). Joint effects of all selected SNPs that fitted in the model were calculated and reported as independent variants’ effects. Regional plots of a 1Mb window flanking the locus sentinel variants (p < 5 × 10^−8^) were generated using LocusZoom^34^ with LD structure estimated in the UK Biobank subcohort (see Figure S3).

### Gene Prioritization

#### Variant Annotation

Sentinel variants (conditional p < 1.03 × 10^−5^) and their proxies (r^2^ < 0.8) were annotated on the human reference genome sequence hg19 using Annovar (v2017July16).^35^ Their functional consequences on the protein sequences encoded by the nearest genes were cross-validated using definitions from RefGene,^36^ Ensembl gene annotation,^37^ GENCODE,^38^ and the University of California, Santa Cruz (UCSC) human genome database.^39^ These variants were also evaluated for features including evolutionary conservation (whether they reside in or specifically encode an conserved element based on multiple alignments across 46 vertebrate species), chromatin states predicted using Hidden Markov Models trained by CHIP-seq data from ENCODE (15 classified states across nine cell types), histone modification markers (active promoter: H3K4Me3, H3K9Ac; active enhancer: H3K4me1, H3K27Ac; active elongation: H3K36me3; and repressed promoters and broad regions: H3K27me3), and CTCF transcription factor binding sites across nine cell lines, conserved putative TFBS, and DNaseI hypersensitive areas curated from the ENCODE database.^38^ Variants within the exonic regions were further annotated with allele frequencies in seven ethnical groups (retrieved from the Exome Aggregation Consortium database) and functional effects prediction performed using a number of different algorithms. For non-coding variants, we performed integrated analysis with SNP Nexus IW scoring.^40^

#### Transcriptomic Data Integration

(1) With summary statistics, we performed a gene-level analysis, using S-PrediXcan, that links LTL to predicted gene expressions across 44 tissues (GTex v6p). It uses multivariate sparse regression models that integrate *cis*-SNPs within 2Mb windows around gene transcript boundaries in order to predict the corresponding gene expression levels. A detailed description of the method can be found elsewhere.^41^^,^^42^ In brief, individual SNP-LTL associations were weighted by SNP-gene (*w*_*lg*_) and SNP-SNP (σl/σg) association matrix, estimated from the PredictDB training set ($zg\;=\;\sum_{l\in g}wlg\;\left(\sigma l/\sigma g\right)\;zl$, for a gene (*g*); the set of SNPs (*l*) were selected from an elastic net model with a mixing parameter of 0.5). Protein-coding genes with qualified prediction model performance (average Pearson’s correlation coefficients r^2^ between predicted and observed gene expressions >0.01, FDR < 0.05) were included in our analysis. We considered a predicted gene expression to be significantly associated with LTL at a Bonferroni corrected p value threshold (p < 2.61 × 10^−7^), conservatively assuming association of each gene in each tissue as an independent test.

(2) For a given region significantly associated with LTL (FDR < 0.05), we tested whether the potential causal variants are shared between LTL and gene expressions by using COLOC Bayesian approach.^43^ Regions for testing were determined as 2Mb windows surrounding the sentinel variants. Regional summary statistics were extracted from this GWA meta-analysis for associations with LTL and GTex v7^44^ for *cis*-eGenes (genes with significant expression quantitative trait loci [eQTLs], FDR < 0.05) located within or on the boundaries of LTL regions defined. We selected the default priors for this analysis. We set p1 = p2 = 10^−4^, meaning that 1 in 10,000 variants is associated with either trait (LTL or gene expression), as has been suggested by others.^43^ We set p12 = 10^−5^, meaning that 1 in 10 (p12/(p12 + p1)) variants that are associated with one trait is also associated with the other. This was chosen because sensitivity analyses have shown broadly consistent results between this setting and more stringent (p12 = 10^−5^) settings, while allowing greater power.^45^ Evidence for colocalization was assessed by comparing the posterior probability (PP) for two hypotheses: that the associations for both traits were driven by the same causal variants (hypothesis 4) and that they were driven by distinct ones (hypothesis 3). Strong evidence of a co-localized eQTL was defined as PP3 + PP4 ≥ 0.99 and PP4/PP3 ≥ 5, and suggestive evidence was defined as PP3 + PP4 ≥ 0.90 and PP4/PP3 ≥ 3, consistent with previous studies.^46^^,^^47^

#### Epigenomic (DNA Methylation) Data Integration

For genes whose expressions are modulated by epigenetic modifications, such as the methylation of transcriptional regulators in *cis*, linking genetic variants associated with *cis*-methylation probes (*cis*-meQTLs, FDR < 0.05) to LTL can help gene prioritization. For this: (1) We conducted a systematic search of LTL-associated sentinel variants and their proxies (r^2^ > 0.8) in multiple publicly available meQTL databases.^48^, ^49^, ^50^ (2) We also performed an epigenome-wide association analysis that integrated multiple variants’ associations in a regularized linear regression model which was algorithmically similar to the transcriptome-wide association analyses.^51^ A reference panel for meQTLs was constructed based on individuals in the EPIC-Norfolk cohort, with detailed description published elsewhere.^52^ Bonferroni correction was applied, accounting for the total number of CpG markers tested (p = 1.00 × 10^−7^).

### Pathway Enrichment Analysis

Using two different approaches, we sought to identify pathways that are responsible for regulating TL.

#### PANTHER

A list of our prioritized genes at each locus (or the nearest gene where no prioritization was possible) was submitted for statistical overrepresentation testing (Fisher’s exact test) in Protein Analysis through Evolutionary Relationships (PANTHER).^53^ Pathways (Gene Ontology [GO] molecular function complete annotation dataset) were considered over-represented where FDR p < 0.05.

#### DEPICT

We also used a hypothesis-free, data-driven approach using Data-driven Expression Prioritized Integration for Complex Traits (DEPICT)^54^ to highlight reconstituted gene sets and tissue and/or cell types where LTL-associated loci were enriched. Summary statistics of uncorrelated SNPs (LD r^2^ ≦ 0.5) significantly associated with LTL at a genome-wide level (p < 5 × 10^−8^) were used as the input, and the HLA region (chr6:29691116–33054976) was excluded. DEPICT first defined each locus around the uncorrelated variants and selected the genes within the region. It then characterized gene functions based on pairwise co-regulation of gene expressions, and these gene functions were quantified as membership probabilities across the 14,461 reconstituted gene sets. Then for each gene set, it assessed the enrichment by testing whether the sum of membership scores of all genes within each LTL-associated locus was higher than that for a gene-density-matched random locus. Detailed description of gene set construction was published elsewhere.^54^ In brief, DEPICT leveraged a broad range of pre-defined pathway-oriented databases to construct gene sets (14,461), including GO terms,^55^ KEGG,^56^ REACTOME pathways,^57^ the experimentally derived protein-protein interaction (PPI) subnetwork,^58^ and the gene-phenotype matrix curated by Mouse Genetics Initiative.^59^ Correlations (r ≧ 0.3) between significant gene sets were visualized using CytoScape.^60^

### Clinical Relevance of LTL

#### Mendelian Randomization

Using two-sample Mendelian randomization (MR)^61^ we investigated the potential effect of LTL on 122 diseases manually curated in the UK Biobank (Table S3).^62^ Diseases were selected where there were sufficient case numbers to detect an odds ratio >1.1 (Table S4). LTL was genetically proxied based on 52 independently associated variants (FDR < 0.05). Individual SNP effects on disease were tested using logistic regression in SNPTEST,^63^ adjusting for sex, age, the first five genetic principal components, and genotyping array within the UK Biobank. MR estimates were calculated using an inverse variance weighted MR approach. Sensitivity analyses were performed using median-based MR,^64^ MR-RAPS,^65^ MR-Eggers,^66^ and MR-Steigers^67^ to identify inconsistency in the MR estimates, account for weak instrument bias, highlight any evidence of directional pleiotropy, and estimate direction of the MR relationship, repectively.

#### LD Score Regression

Cross-trait linkage disequilibrium score regression (LDSC) analysis was used to measure genetic correlations between LTL and selected traits through the use of the LD Hub database (version 1.4.1).^68^ From the 832 available traits in LD Hub, we *a priori* selected traits of interest in order to remove redundancy and/or duplication within the analysis. We removed poorly defined traits and diseases, those without prior evidence of a genetic basis, and medications. We also removed lipid sub-fractions because we thought these unlikely to be relevant. We excluded studies with a sample size <1,000. Where multiple datasets for the same trait existed, we first prioritized datasets from large specialist consortia (where relevant factors would have been accounted for within the GWAS analysis) over the UK Biobank analyses conducted by the Neale group (where the GWAS was acknowledged to be a “quick and dirty” analysis). We then prioritized larger sample size, more recent studies, and diagnosed conditions over self-reported ones. We also removed traits with low heritability estimates within LD Hub, leaving us with 320 traits (information, including PMIDs of the selected studies, is given in the Results section).

Genome-wide summary statistics were used as the input, and standardized quality control was implemented within the software, including minor allele frequency (MAF) (>1% for HapMap3 and >5% for 1000 Genomes EUR-imputed SNPs), effective sample size (>0.67 times the 90^th^ percentile of sample size), removal of insertions or deletions or structural variants, allelic alignment to 1000 Genomes, and removal of SNPs within the major histocompatibility complex (MHC) region.

#### Variants-based Cross-database Query

Independent variants and their strong proxies (r^2^ ≥ 0.8) were queried against publicly available GWAS databases; for this, we used PhenoScanner^69^ for computational efficiency. A list of GWAS results implemented in the software was previously published. Results were filtered to include associations with p < 1 × 10^−6^, in high LD (r^2^>0.8) with the most significant SNPs within the region, and manually curated to retain only the most recent and largest study per trait.

## Results

### Discovery of Genetic Determinants of LTL

Mean LTL was measured within each cohort by using a quantitative polymerase chain reaction (qPCR)-based method, which expresses TL as a ratio of telomere repeat content (T) to single-copy gene (S) within each sample (see Subjects and Methods, Supplemental Information, and Table S1). T/S ratios were z-standardized to harmonize differences in the quantification and calibration protocols between cohorts. Associations of shorter LTL with increasing age and male gender were observed as expected (Table S1).

Variants were assessed for association with mean LTL within each cohort through the use of additive models adjusted for age, gender, and cohort-specific covariates and then combined using inverse-variance-weighted meta-analysis (Table S2).

In total, 20 sentinel variants at 17 genomic loci were independently associated with LTL at a level of genome-wide statistical significance (p < 5 × 10^−8^, Table 1, Figure S1), including six loci that had not previously been associated with LTL (*SENP7* [MIM: 612846], *MOB1B* [MIM:609282], *CARMIL1* [MIM: 609593], *PRRC2A* [MIM: 142580], *TERF2*, and *RFWD3* [MIM: 614151]). We also identified genome-wide significant variants in four recently reported loci from a Singaporean Chinese population (*POT1*, *PARP1* [MIM: 173870], *ATM* [MIM:607585], and *MPHOSPH6* [MIM:605500])^70^ and confirmed association at seven previously reported loci in European ancestry studies (*TERC*, *NAF1* [MIM: 617868], *TERT*, *STN1(OBFC1)*, *DCAF4* [MIM: 616372], *ZNF208* [MIM: 603977], and *RTEL1*).^15^^,^^23^ Two and three conditionally independent signals were detected within the *TERT* and *RTEL1* loci, respectively (Table 1). Within the known loci, three variants within the *DCAF4* (r^2^ = 0.05) and *TERT* (r^2^ < 0.5) loci were distinct from the previously reported sentinel variants, while five (*TERC*, *NAF1*, *STN1*, *ZNF208*, and *RTEL1*; r^2^ > 0.8; Table S5) were in high LD with the previously reported ones from European studies. For the loci identified in a Chinese ancestry population, we observed the same sentinel variant for *PARP1* and high LD variants for *ATM* and *MPHOSPH6* (r^2^ > 0.8) but a distinct sentinel for *POT1* (r^2^ < 0.5, Table S5). While we observed a distinct sentinel for *POT1*, we cannot rule out the possibility that the association signal observed in this region could be shared. In that case, the sentinels identified in each population would be reflective of a third, as yet unidentified, variant that is the true causal variant in this region. For the *RTEL1* locus, there are significant differences in LD structure between ancestral populations. All of the *RTEL1* variants we report at genome-wide statistical significance are in low LD with those reported in Singaporean Chinese and in South Asians.^25^^,^^70^ Our novel variants are of lower frequency (MAF < 0.1) and either are reported as being monoallelic (monomorphic) or fall below the MAF threshold for analysis in the Southern Han Chinese (CHS) population (MAF < 0.01). This suggests that genetic variation in this region may be, in part, population specific or that the MAF is so low that we currently are unable to detect any association.

**Table 1:** Independent Variants Associated with LTL at Genome-Wide Significance (5x10^−8^)

**SNP**	**Gene**	**Chr**	**Position (hg19)**	**EA**	**EAF**	**Beta**	**SE**	**p Value**
**Previously Reported Loci**								

rs3219104	*PARP1*	1	226562621	C	0.83	0.042	0.006	9.60 × 10^−11^
rs10936600	*TERC*	3	169514585	T	0.24	−0.086	0.006	7.18 × 10^−51^
rs4691895	*NAF1*	4	164048199	C	0.78	0.058	0.006	1.58 × 10^−21^
rs7705526	*TERT*	5	1285974	A	0.33	0.082	0.006	5.34 × 10^−45^
rs2853677∗	*TERT*	5	1287194	A	0.59	−0.064	0.006	3.35 × 10^−31^
rs59294613	*POT1*	7	124554267	A	0.29	−0.041	0.006	1.17 × 10^−13^
rs9419958	*STN1 (OBFC1)*	10	105675946	C	0.86	−0.064	0.007	5.05 × 10^−19^
rs228595	*ATM*	11	108105593	A	0.42	−0.029	0.005	1.43 × 10^−8^
rs2302588	*DCAF4*	14	73404752	C	0.10	0.048	0.008	1.68 × 10^−8^
rs7194734	*MPHOSPH6*	16	82199980	T	0.78	−0.037	0.006	6.94 × 10^−10^
rs8105767	*ZNF208*	19	22215441	G	0.30	0.039	0.005	5.42 × 10^−13^
rs75691080	*RTEL1/STMN3*	20	62269750	T	0.09	−0.067	0.009	5.99 × 10^−14^
rs34978822∗	*RTEL1*	20	62291599	G	0.02	−0.140	0.023	7.26 × 10^−10^
rs73624724∗	*RTEL1/ZBTB46*	20	62436398	C	0.13	0.051	0.007	6.33 × 10^−12^

**Additional Loci**								

rs55749605	*SENP7*	3	101232093	A	0.58	−0.037	0.007	2.45 × 10^−8^
rs13137667	*MOB1B*	4	71774347	C	0.96	0.077	0.014	2.43 × 10^−8^
rs34991172	*CARMIL1*	6	25480328	G	0.07	−0.061	0.011	6.19 × 10^−9^
rs2736176	*PRRC2A*	6	31587561	C	0.31	0.035	0.006	3.53 × 10^−10^
rs3785074	*TERF2*	16	69406986	G	0.26	0.035	0.006	4.64 × 10^−10^
rs62053580	*RFWD3*	16	74680074	G	0.17	−0.039	0.007	4.08 × 10^−8^

Gene—the closest or candidate gene (known telomere-related function) within the region. EA—effect allele. EAF—effect allele frequency within the study. Beta—the per-allele effect on z-scored LTL. SE—standard error.

∗ Additional, independent signals detected using conditional analysis are included.

It has been shown that many loci that fall just below the conventional threshold of genome-wide significance are genuinely associated with the trait of interest and do subsequently reach the conventional threshold when sample size is increased.^71^ In an attempt to gain additional insight into the genetic determination of LTL in humans, we applied a less stringent FDR threshold to the data. An additional 32 variants met an FDR threshold of <0.05, totaling 52 variants that estimate ~2.93% of the variance in TL (Table S6).^71^ Within this FDR list, 5% of variants (2–3) are estimated to be false positives, although we are not able to determine which they are. While we believe that this FDR is acceptable, we advise that individual loci should be interpreted with some caution. These variants were located within separate loci from those reported above, with the exception of a fourth, independent signal in the *RTEL1* locus. Although we did not replicate the previously reported *ACYP2* (MIM: 102595) locus, this did remain within the variants identified at the FDR < 0.05 threshold. *TYMS* (MIM: 188350), identified as genome-wide significant in a trans-ethnic meta-analysis of Singaporean Chinese^67^ and in the previously reported ENGAGE analysis,^15^ is within our FDR < 0.05 identified loci. This was to be expected considering the substantial sample overlap of the ENGAGE data; however, our sentinel variant is distinct and not reported in the Dorajoo et al. study. Aligning our data with available summary statistics from the Dorajoo et al. study (Singaporean Chinese samples only), we see at least nominal support for the vast majority of our genome-wide significant loci, with the exception of *STN1(OBFC1)* and *SENP7* (Table S7). Although *SENP7* has not previously been reported, variants in high LD (r^2^ > 0.6) with our *STN1* sentinel have been reported in other European populations.^21^^,^^22^ There is also support for many variants in our extended FDR list. However, it should be noted that data are not available for around half of our FDR < 0.05 loci, with most of these being either monoallelic or too low frequency to have been included within the analysis in the CHS population, again suggesting that several may be specific to the European population.

### Prioritization of Likely Candidate Genes

We applied *in silico* prediction tools, leveraging large-scale human genomic data integrated with multi-tissue gene expression, transcriptional regulation, and DNA methylation data, coupled with knowledge-driven manual curation, to prioritisze the genes that are most likely influenced by the genetic variants within each locus. All 52 sentinel variants identified at GWS and FDR < 0.05 (listed in Table S6) plus their high LD proxies (r^2^ > 0.8) were taken forward into our *in silico* analyses. First, we annotated all variants for genomic location and location with respect to regulatory chromatin marks (Tables S8 and S9). This also identified variants that led to non-synonymous changes in nine loci. Of these, five loci contained variants with predicted damaging effects on protein function (Table S10). We also found evidence that variants were associated with changes in gene expression in multiple loci (Table S11), with several showing co-localization and evidence from two approaches. This data, along with prediction of functional non-coding variants (Table S12), methylation QTL data (Table S13), and curation of gene functions within the region (Supplemental Methods), are summarized in Table S14. The summary data were utilized to prioritize genes that are most likely influenced at each locus. Where the prioritization methods suggested multiple genes for a given locus, we prioritized based on the amount of evidence across all considered lines of enquiry stated above. We were able to prioritize genes at 15 of the 17 genome-wide significant loci and 16 at of the 32 FDR loci (Table S14).

**Figure 1:**
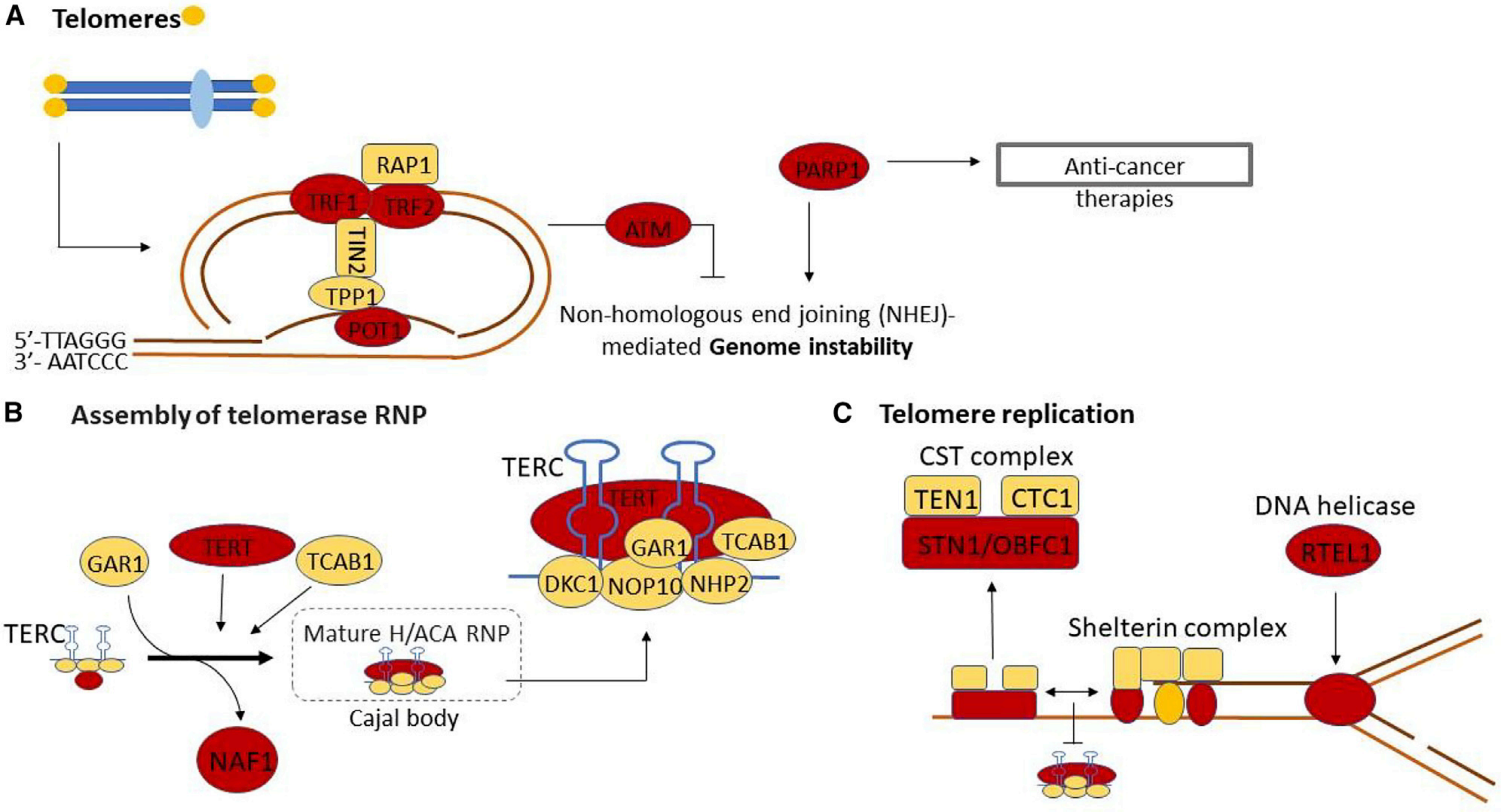
Loci with Established Roles in Telomere Biology Candidate genes found in this study are shown in red. These include genes that encode components of the SHELTERIN complex (A), regulate the formation and activity of telomerase (B), and regulate telomere structure (C).

Four of the prioritized genes for newly identified loci have known roles in telomere regulation (*PARP1*, *POT1*, *ATM*, and *TERF2*; Figure 1). PARP1 (poly(ADP-ribose) polymerase 1), a variant in high LD (r^2^ = 1.0) with our identified sentinel variant, causes a Val762Ala substitution (Table S10) which is known to reduce PARP1 activity.^72^ This variant was associated with shorter LTL, in agreement with studies showing that knockdown of *PARP1* leads to telomere shortening.^73^ PARP1 catalyzes the poly(ADP-ribosyl)ation of proteins in several cellular pathways, including DNA repair.^73^ It interacts with TERF2 and it regulates the binding of TERF2 to telomeric DNA through this post-translational modification.^74^

Three genes, *DCAF4*, *SENP7*, and *RFWD3*, prioritized based on deleterious protein coding changes (*DCAF4*, *SENP7*) or strong evidence linking to gene expression levels (*RFWD3*), are all involved in DNA damage repair.^75^, ^76^, ^77^ SENP7 has previously been demonstrated to bind damaged telomeres.^78^ Components of DNA damage response and repair pathways (such as ATM) have been shown to also play roles in telomere regulation.^79^ Mutations in *RFWD3* cause Fanconi anemia (MIM: 617784), a disease linked to telomere shortening and/or abnormalities.^80^

The *PRRC2A* locus contains 11 genetically linked SNPs located across the MHC class III region, which is a highly polymorphic and gene-dense region with complex LD structure. *BAG6* (MIM: 142590) and *CSNK2B* (MIM: 115441) were suggested as gene candidates for this region, supported by gene expression data (see Supplemental Information and Tables S11 and S14). BAG6 is linked to DNA damage signaling and apoptosis,^81^ while CSNK2B, a subunit of casein kinase 2, interacts with TERF1 and regulates TERF1 binding at telomeres.^82^

### Pathway Enrichment

To investigate context-specific functional connections between prioritized genes of the identified loci and to suggest plausible biological roles of these genes in the TL regulation, we performed enrichment analyses for pathways and tissues through the use of DEPICT^54^ and PANTHER.^53^ DEPICT is a hypothesis-free, data-driven approach for which we used summary statistics of all uncorrelated SNPs (LD r^2^ ≦ 0.5) associated at p < 5 × 10^−8^ as input. For PANTHER, we assessed overrepresentation of genes within our loci within known pathways. To minimize noise, we used our prioritized genes as input, along with the closest gene to the sentinel SNP, where no prioritization was possible. In total, 55 genes were submitted to PANTHER, of which six were not available within PANTHER, leaving 49 within the analysis.

**Figure 2:**
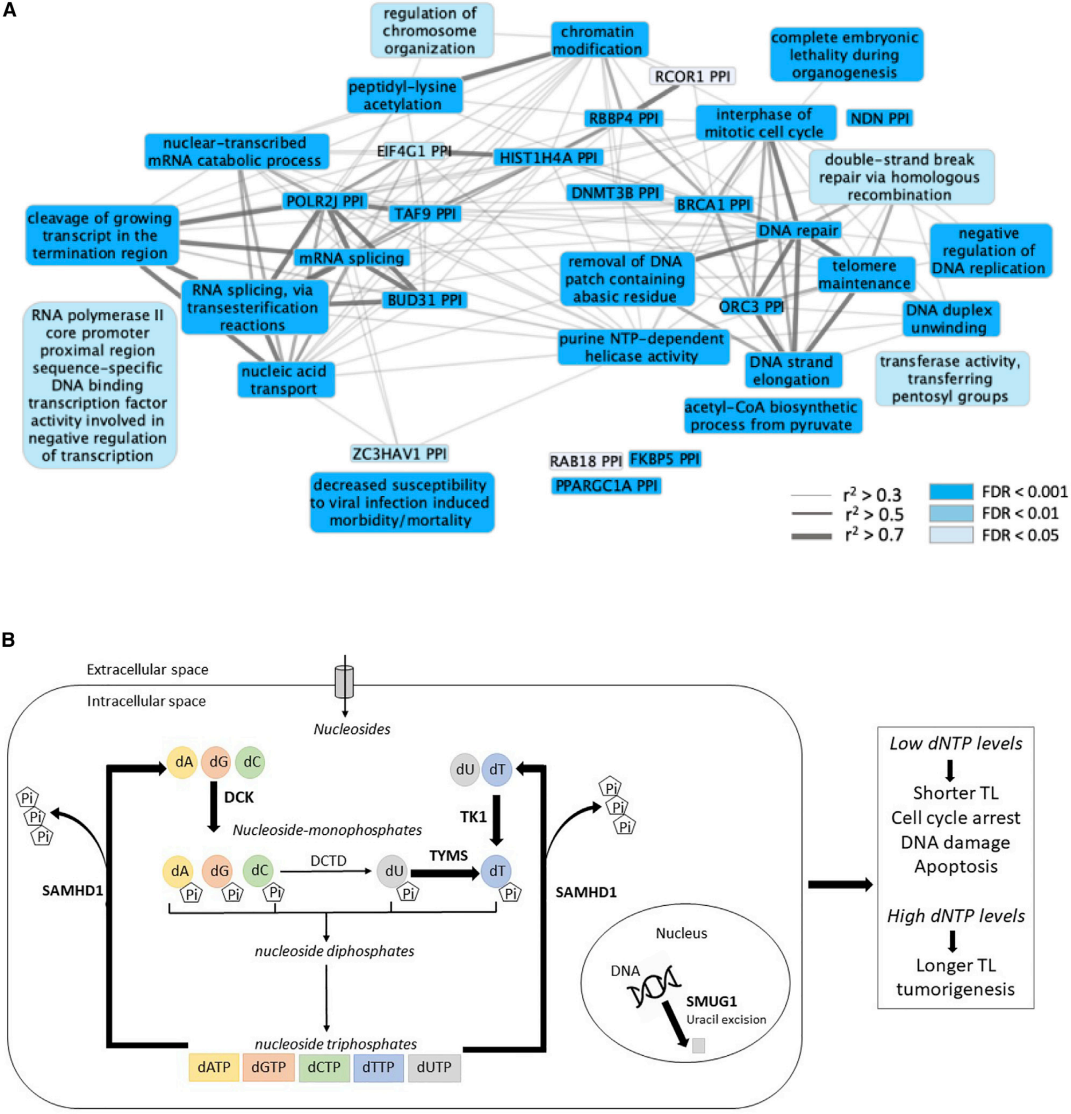
Pathways Enriched for Telomere-Associated Genes (A) Gene sets significantly (false discovery rate [FDR] < 0.05) enriched for prioritised LTL-associated genes. Color intensity of the nodes (gene sets), classified into three levels, reflects enrichment strengths (FDR). Edge width indicates Pearson correlation coefficient (r^2^) between each pair of the gene sets. Some of the most significantly associated gene sets include telomere maintenance along with DNA replication and repair pathways as may be expected. How other enriched pathways may influence LTL is unclear. (B) Role of LTL-associated genes in nucleotide metabolism. Five enzymatic reactions and genes encoding the corresponding enzymes prioritized from this GWAS are highlighted in bold.

Over 300 reconstituted gene sets (DEPICT) were significantly enriched for the LTL loci (FDR < 0.05); these could be further clustered into 34 meta-gene sets, highlighting pathways that are involved in several major cellular activities, including DNA replication, transcription, and repair; cell cycle regulation; immune response; and intracellular trafficking (Figure 2A).

The PANTHER analysis identified a number of telomere-related pathways, including regulation of telomeric loop disassembly, t-circle formation, protein binding at telomeres, and single-strand break repair, as being the mostly highly overrepresented (Table S15). Among other expected pathways, cellular aging and senescence were also highlighted. Of note, nucleotide metabolism pathways were overrepresented (2′-deoxyribonucleotide metabolic process, deoxyribose phosphate metabolic process, and deoxyribonucleotide metabolic process; Figure 2B; Table S15). The genes matched to these pathways were *TYMS*, *SAMHD1* (MIM: 606754), and *SMUG1* (MIM: 607753). While TYMS is critical for deoxythymidine monophosphate (dTMP) biosynthesis, SAMHD1 controls deoxynucloeside triphosphate (dNTP) catabolism and SMUG1 removes misincorporated uracil from DNA.^83^, ^84^, ^85^ Although not highlighted in the pathway analysis, two further genes within other identified loci (*TK1* [MIM: 188300] and *DCK* [MIM:125450]) are key regulators of deoxynucleoside monophosphate (dNMP) biosynthesis;^85^ this adds further support to the possibility that nucleotide metabolism is a key pathway in regulating LTL. dNTPs constitute the fundamental building blocks required for DNA replication and repair.^86^ Genetic perturbations that disrupt dNTP homeostasis have been shown to result in increased replication error, cell cycle arrest, and DNA-damage-induced apoptosis.^85^^,^^87^

### Relationship between Genetically Determined TL and Disease

**Figure 3:**
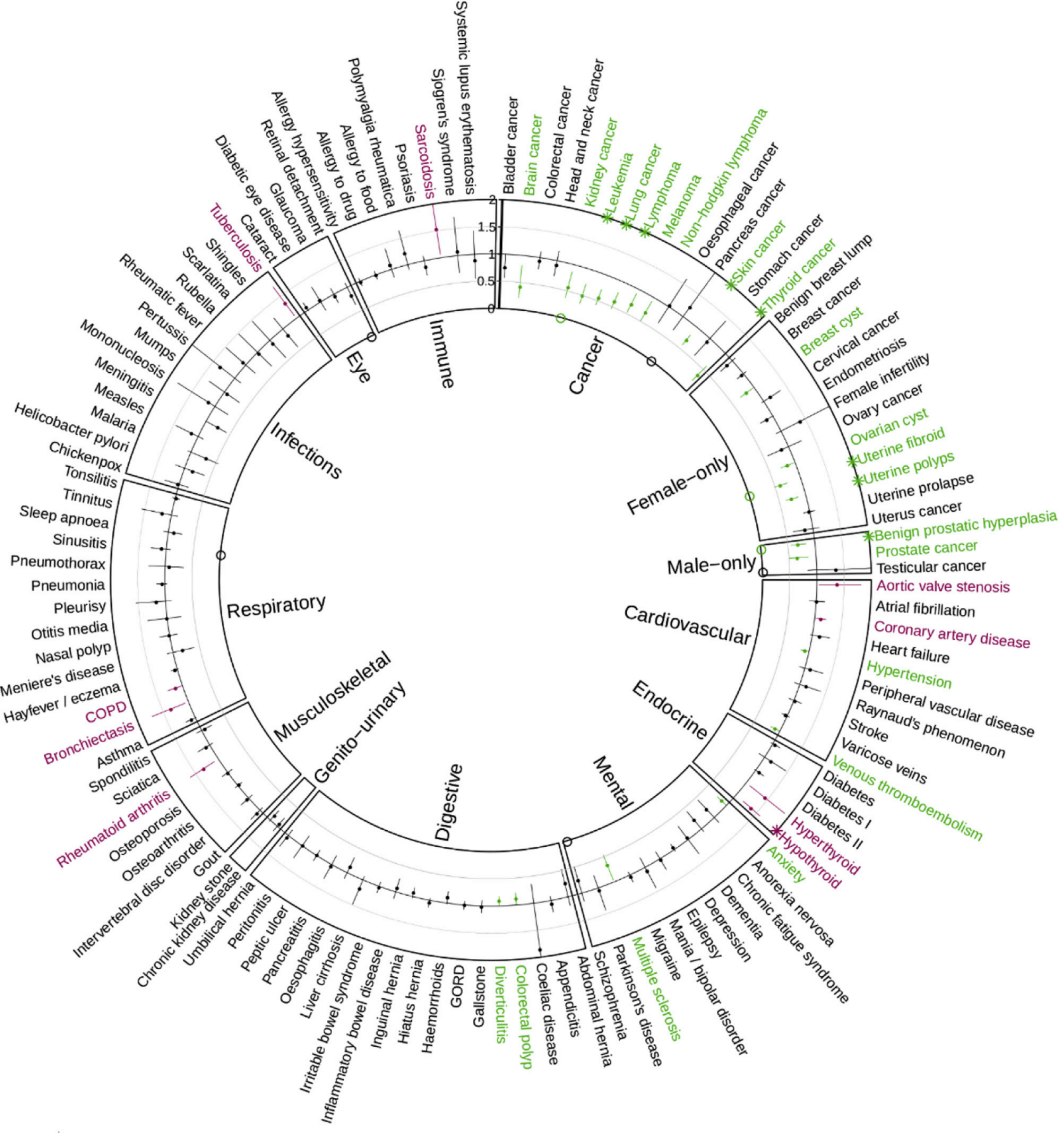
Mendelian Randomization Results for the Effect of Shorter LTL on the Risk of 122 Diseases in the UK Biobank Data shown are odds ratios and 95% confidence intervals for a 1 standard deviation shorter LTL. Diseases are classified into groups, as indicated by the boxing, and sorted alphabetically within disease group. Nominally significant (p < 0.05) associations estimated via inverse-variance-weighted Mendelian randomization are shown in green for a reduction in risk and purple for an increase in risk due to shorter LTL. ^O^ indicates nominal (p < 0.05) evidence of pleiotropy estimated by MR-Eggers intercept. Full results are also shown in Table S16 along with the full MR sensitivity analysis.

To further understand the clinical relevance of TL, we used the 52 independent variants identified at FDR < 0.05 as genetic instruments for TL, and we applied a two-sample MR approach using UK Biobank data.^62^ We manually curated 122 diseases available in the UK Biobank and examined their relationships with shorter TL (Tables S3 and S16). We observed nine associations which passed a Bonferroni corrected threshold (p < 4.1x10^−4^). These included novel findings of an increased risk of hypothyroidism, and decreased risk of thyroid cancer, lymphoma, and diseases of excessive growth (uterine fibroids, uterine polyps, and benign prostatic hyperplasia). We also confirmed findings for decreased risk of lung and skin cancer and leukemia for subjects with shorter TL (Figure 3, Table S16).^16^^,^^18^^,^^88^ We observed a further 30 nominally significant associations (p < 0.05), confirming previous MR findings of an increased risk of CAD, within the UK Biobank population (Figure 3, Table S16). Our results also provide genetic evidence for associations of shorter LTL with increased risk of rheumatoid arthritis, aortic valve stenosis, chronic obstructive pulmonary disease, and heart failure, all of which have previously been observationally associated with shorter LTL.^89^, ^90^, ^91^, ^92^ We also ran the MR analyses using only the genome-wide significant variants (Figure S4), and we did not lose any Bonferroni-significant hits, with only small differences in those diseases that are nominally associated. In our sensitivity analyses, effect estimates were consistent across MR methods. The MR-Steigers analysis indicated that the direction of the relationship is that TL influences disease risk. This analysis also indicated that this direction was estimated correctly for the majority of diseases (Table S16).

We next sought to explore human diseases and traits that share common genetic etiologies with LTL. We did this by performing LD score regression analyses to test for genetic correlations between TL and 320 curated traits and diseases (Table S17) within LD Hub.^15^^,^^16^ In comparison to the MR approach, these analyses utilize genome-wide genetic information rather than selected SNPs with the most significant associations. In agreement with our MR analyses, TL was negatively correlated with CAD (r = −0.17, p = 0.01, Table S17). Dyslipidaemia risk factors for CAD also showed concordant associations with shorter TL, including higher LDL and total cholesterol and lower HDL cholesterol (Table S17). These results are suggestive of a shared genetic architecture underlying TL, CAD, and CAD risk factors. However, these results would not survive correction for multiple testing.

We also examined individual locus-driven genetic correlations between TL and a variety of human phenotypes and diseases by using PhenoScanner^69^ to query 52 FDR sentinel variants and their closely related SNPs in LD (r^2^ ≧ 0.8) against publicly available GWAS databases. While some morbidities showed specific correlations to a single locus, others showed correlations to a broader spectrum of loci. For example, self-reported hypothyroidism or myxoedema exhibited a strong association particularly at the *TERT* locus, which was also exclusively responsible for several subtypes of ovarian cancers (Table S18). In contrast, blood cell traits and hematological diseases were implicated with a wider range of loci, including *TERC*, *TERT*, *SENP7*, *ATM*, *BBOF1*, and *MROH8*; this result is similar to those for the respiratory function and lung cancers that also involved multiple TL loci (Table S18).

## Discussion

We identify 20 lead variants at a level of genome-wide significance and a further 32 at FDR < 0.05. Within established loci, we report a second, independent, association signal within the *TERT* locus and redefine the *RTEL1* locus into three independent signals. By applying a range of *in silico* tools that integrate multiple lines of evidence, we were able to pinpoint likely influenced genes for the majority of independent lead variants (34 of 52), several of which represent key telomere-regulating pathways (including components of the telomerase complex, the telomere-binding SHELTERIN and CST complexes, and the DNA damage response [DDR] pathway).

Telomeres function to prevent the 3′ single-stranded overhang at the end of the chromosome from being detected as a double-stranded DNA break. This is achieved through binding of the SHELTERIN complex (TERF1, TERF2, TERF2IP, TINF2, ACD, and POT1), which acts to block activation of DDR pathways via several mechanisms.^3^ SHELTERIN also binds a number of accessory factors that facilitate processing and replication of the telomere, including the DNA helicase RTEL1.^3^ SHELTERIN also interacts with the CST complex that regulates telomerase access to the telomeric DNA (Figure 1C).^3^ The associated loci contain two of the SHELTERIN components (TERF2 and POT1), a regulator of TERF1, CSNK2B (*PRRC2A* locus),^82^ the helicase RTEL1, and the CST component STN1.

Although telomere-binding proteins and structure aim to inhibit activation of DDR pathways, there is also evidence of a paradoxical involvement of a number of DDR factors in TL maintenance; these factors include both of the prioritized genes, *ATM* and *PARP1*.^73^^,^^93^ TERF2 inhibits ATM activation and the classical non-homologous end joining (c-NHEJ) at telomeres, thus preventing synapsis of chromosome ends (Figure 1A).^94^ However, ATM activation is required for telomere elongation, potentially by regulating access of telomerase to the telomere end through ATM-mediated phosphorylation of TERF1.^93^ It is possible that other DDR regulators can impact TL maintenance by regulating telomeric chromatin states, T-loop dynamics, and single-stranded telomere overhang processing.^79^ Other prioritised genes (*SENP7* and *RFWD3*) also function within DDR pathways; this suggests a plausible mechanism through which they may influence LTL.

The telomerase enzyme is capable of extending telomeres and/or compensating sequence loss due to the end replication problem in stem and reproductive cells.^4^ Associated loci include genes encoding the core telomerase components TERT and TERC along with the chaperone protein NAF1. NAF1 is required for *TERC* accumulation and its incorporation into the telomerase complex.^95^ After transcription, *TERC* undergoes complex 3′ processing to produce the mature 451bp template.^96^ This involves components of the RNA exosome complex, PARN (MIM: 604212) and TENT4B (MIM: 605540), among others; this process is not fully understood.^97^ In addition to variants within regions containing *TERT*, *TERC*, and *NAF1*, a prioritiszed gene from another locus (*MPHOSPH6*) is a component of the RNA exosome.^98^

Comparing our findings to those reported in a non-European study,^70^ we find support for our most significantly associated loci. For many of our FDR < 0.05 loci, we were unable to look for support from this study because our sentinel variants were either monoallelic or rare (MAF < 0.01) in the CHS population. Different LD structures in regions such as RTEL1, coupled with the reported absence of some of the variants in other ancestral populations, suggest that some of our reported variants may specific to Europeans. Adding additional support for the existence of population-specific rare variants regulating LTL is the discovery of two loci in the Singaporean Chinese study that are monoallelic in Europeans.^70^ Because both of these replicate within CHS subjects and are located within regions containing telomere-related genes, they are unlikely to be false positive findings. Future large-scale trans-ethnic meta-analyses will be critical in determining shared causal variants from population-specific rare variants. This is of key importance to downstream analyses using genetically determined LTL to investigate disease risk in different populations. However, the current lack of large-scale data on LTL in non-European cohorts is limiting.

Utilizing the prioritized gene list as well as the closest genes to the sentinel variants, we showed a number of pathways to be enriched for telomere-associated loci. Of note, we observed significant overrepresentation of genes in several nucleotide metabolism pathways (Table S15, Figure 2B). Key genes were highlighted by this function in both the biosynthesis (*TYMS*, *TK1*, and *DCK*) and catabolism (*SAMHD1*) of dNTPs. Biosynthesis of dNTPs occurs via two routes: de-novo synthesis and the nucleotide salvage pathway. Thymidine kinase (TK1) and deoxycytidine kinase (DCK) are the rate-limiting enzymes that catalyze the first step of the salvage pathway of nucleotide biosynthesis, converting deoxynucleosides to their monophosphate forms (dNMPs) before other enzymes facilitate further phosphorylation into deoxynucleodie diphophates (dNDPs) and dNTPs (Figure 2B).^85^ Thymidylate synthetase (TYMS) is considered to be a component of the *de novo* pathway, and is the key regulator of dTMP biosynthesis, converting deoxyuridine monophosphate (dUMP) to dTMP.^85^ However, because the dUMP substrates can be derived from either *de novo* synthesis or deamination of deoxycytidine monophosphate (dCMP) produced from the salvage pathway, it could be considered to function within both pathways (Figure 2B).^85^ Besides controlling biosynthetic pathways, the equilibrium of cellular dNTP levels is also achieved by regulating degradation of dNTPs, a key regulator of which is SAMHD1. It catalyzes the hydrolysis of dNTPs to deoxynucleosides and triphosphates, thereby preventing the accumulation of excess dNTPs (Figure 2B).^81^ Although the finely tuned dNTP supply system inhibits incorrect insertions of bases into DNA synthesis, potential errors are monitored by the product of another prioritized gene, the base excision repair enzyme, SMUG1, which removes uracil and oxidized derivatives from DNA molecules.^84^

A balanced cellular pool of dNTPs is required for DNA replication and repair and for maintaining proliferative capacity and genome stability. Low levels of dNTPs can induce replication stress, subsequently leading to increased mutation rates.^99^ A surplus of dNTPs, on the other hand, reduces replication fidelity, thus also causing higher levels of spontaneous mutagenesis.^100^ A dynamic balance between biosynthesis and catabolism is required to maintain an equilibrium. Because maintaining the balance of the intracellular dNTP pool is also fundamental to other pathways that are implicated in telomere homeostasis, including cellular proliferation and DNA repair, disruption of dNTP homeostasis may trigger a sequence of cellular events that interplay synergistically, leading to abnormalities of TL and genome instability.

By clustering our prioritized genes via their functional connections, we highlighted a number of pathways that were enriched for TL regulation, which included DNA replication, transcription, and repair; cell cycle regulation; immune response; and intracellular trafficking. However, we noted that because the gene prioritization was based on integration of bioinformatic evidence from a number of publicly available databases, which also laid the foundation for establishing the pathways used in the enrichment analyses, this approach may suffer from self-fulfilling circular arguments.

While supporting previous evidence linking shorter TL to an increased risk of CAD and lower risk of several cancers, we demonstrated additional associations between TL and thyroid disease, thyroid cancer, lymphoma, and several non-malignant neoplasms. Shorter TL was protective against all of these proliferative disorders, potentially through limiting cell proliferative capacity, which in turn reduces the occurrence of potential oncogenic mutations that can occur during DNA replication. Furthermore, we also provide evidence suggesting that shorter TL is potentially causally associated with increased risk of several cardiovascular, inflammatory, and respiratory disorders that have previously been linked to TL in observational studies. Our findings linking nucleotide metabolism to TL regulation could in part explain the link between TL and cancer and proliferative disorders. This would suggest that cells with longer TL have higher dNTP levels that lead to higher proliferation rates and reduced DNA replication fidelity leading to higher mutation rates.

In summary, our findings substantially expand current knowledge on the genetic determinants of LTL, and they elucidate genes and pathways that regulate telomere homeostasis and their potential impact on human diseases and cancer development.

## Declaration of Interests

A.S.B. holds grants unrelated to this work from AstraZeneca, Merck, Novartis, Biogen, and Bioverativ/Sanofi.

J.D. reports personal fees and non-financial support from Merck Sharpe and Dohme UK Atherosclerosis; personal fees and non-financial support from Novartis Cardiovascular and Metabolic Advisory Board; personal fees and non-financial support from Pfizer Population Research Advisory Panel; and grants from the British Heart Foundation, the European Research Council, Merck, the NIHR, NHS Blood and Transplant, Novartis, Pfizer, the UK Medical Research Council, Health Data Research UK, and the Wellcome Trust outside the submitted work.

## Web Resources

Ensembl Genome Browser, https://useast.ensembl.org/index.html
GENCODE, https://www.gencodegenes.org/
Online Mendelian Inheritance in Man, https://www.omim.org/
UCSC Genome Browser, https://genome.ucsc.edu/

## Supplemental Data

Document S1 Figures S1–S4, Supplemental Methods, Description of Individual Loci Associated with LTL, Supplemental Acknowledgments, Supplementary References

Document S2. Tables S1–S18

Document S3. Article plus Supplemental Information
