# Supplementary material for: Genome-wide Association Analysis in Humans Links Nucleotide Metabolism to Leukocyte Telomere Length
Source: Am J Hum Genet. 2020 Feb 27;106(3):389–404. doi: 10.1016/j.ajhg.2020.02.006 (PMC7058826; doi:10.1016/j.ajhg.2020.02.006)
Supplement: Document S1 — Figures S1–S4, Supplemental Methods, Description of Individual Loci Associated with LTL, Supplemental Acknowledgments, Supplementary References [file mmc1.pdf]

The American Journal of Human Genetics, Volume *106*

Supplemental Data

Genome-wide Association Analysis in Humans Links Nucleotide Metabolism to Leukocyte Telomere Length

Chen Li, Svetlana Stoma, Luca A. Lotta, Sophie Warner, Eva Albrecht, Alessandra Allione, Pascal P. Arp, Linda Broer, Jessica L. Buxton, Alexessander Da Silva Couto Alves, Joris Deelen, Iryna O. Fedko, Scott D. Gordon, Tao Jiang, Robert Karlsson, Nicola Kerrison, Taylor K. Loe, Massimo Mangino, Yuri Milaneschi, Benjamin Miraglio, Natalia Pervjakova, Alessia Russo, Ida Surakka, Ashley van der Spek, Josine E. Verhoeven, Najaf Amin, Marian Beekman, Alexandra I. Blakemore, Federico Canzian, Stephen E. Hamby, Jouke-Jan Hottenga, Peter D. Jones, Pekka Jousilahti, Reedik Mägi, Sarah E. Medland, Grant W. Montgomery, Dale R. Nyholt, Markus Perola, Kirsi H. Pietiläinen, Veikko Salomaa, Elina Sillanpää, H. Eka Suchiman, Diana van Heemst, Gonneke Willemsen, Antonio Agudo, Heiner Boeing, Dorret I. Boomsma, Maria-Dolores Chirlaque, Guy Fagherazzi, Pietro Ferrari, Paul Franks, Christian Gieger, Johan Gunnar Eriksson, Marc Gunter, Sara Hägg, Iiris Hovatta, Liher Imaz, Jaakko Kaprio, Rudolf Kaaks, Timothy Key, Vittorio Krogh, Nicholas G. Martin, Olle Melander, Andres Metspalu, Concha Moreno, N. Charlotte Onland-Moret, Peter Nilsson, Ken K. Ong, Kim Overvad, Domenico Palli, Salvatore Panico, Nancy L. Pedersen, Brenda W.J. H. Penninx, J. Ramón Quirós, Marjo Riitta Jarvelin, Miguel Rodríguez-Barranco, Robert A. Scott, Gianluca Severi, P. Eline Slagboom, Tim D. Spector, Anne Tjonneland, Antonia Trichopoulou, Rosario Tumino, André G. Uitterlinden, Yvonne T. van der Schouw, Cornelia M. van Duijn, Elisabete Weiderpass, Eros Lazzerini Denchi, Giuseppe Matullo, Adam S. Butterworth, John Danesh, Nilesh J. Samani, Nicholas J. Wareham, Christopher P. Nelson, Claudia Langenberg, and Veryan Codd

Supplemental Data

Supplemental Figure 1. Study design. Schematic graph to illustrate study design of the LTL GWAS meta-analysis. GWAS was conducted in each individual study cohort, stratified by genotyping platform and disease status. SNP genotyping, GWAS and meta-analyses as well as the corresponding QC procedures are described within the methods.

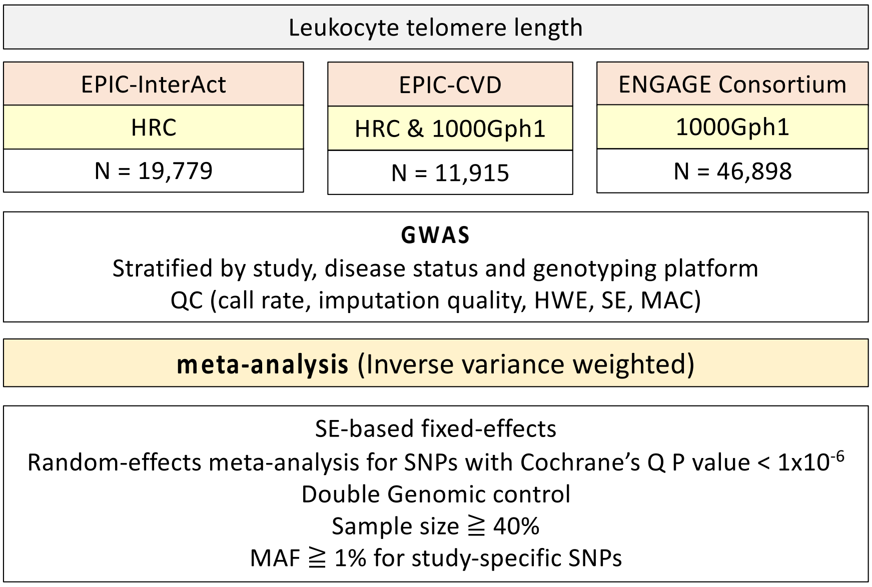

Supplemental Figure 2. Manhattan Plot of GWAS results. Manhattan plot with quantile-quantile plot inlay. Known loci were labelled in blue, novel loci associated with LTL at genome-wide significance (p-value<$5x10^{-8}$, red line) in red, and at FDR threshold of 5% (blue line) in orange.

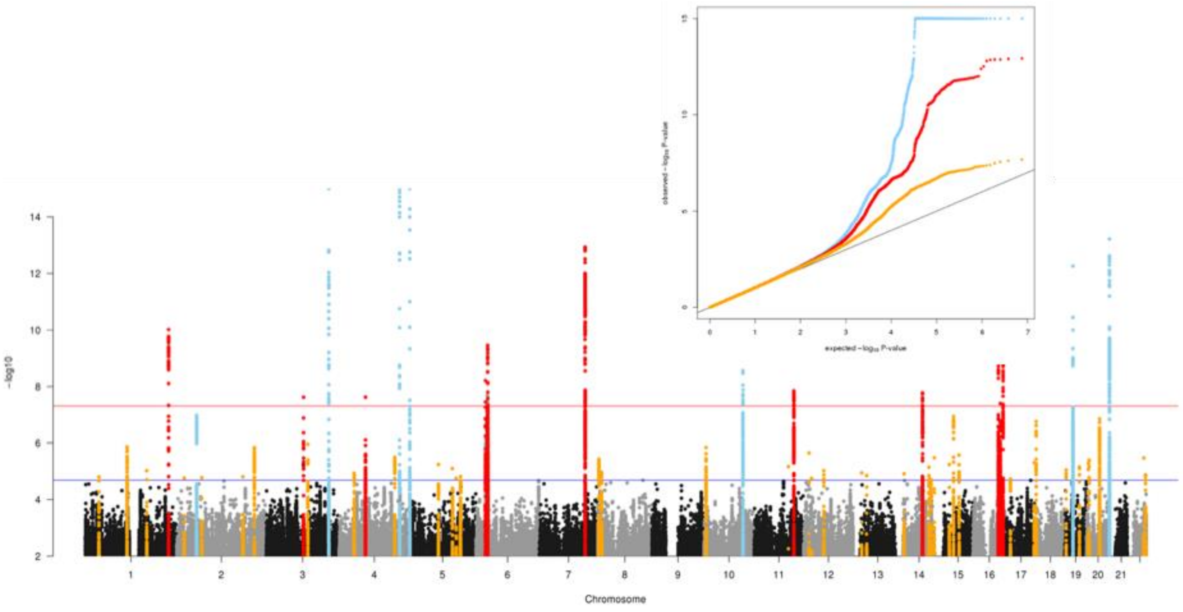

Supplementary Figure 3. Regional plots of genome-wide significant loci ($p<5x10^{-8}$). For all loci 400kb windows encompassing conditionally independent variants, except the TERT locus which is illustrated as a 200kb window.

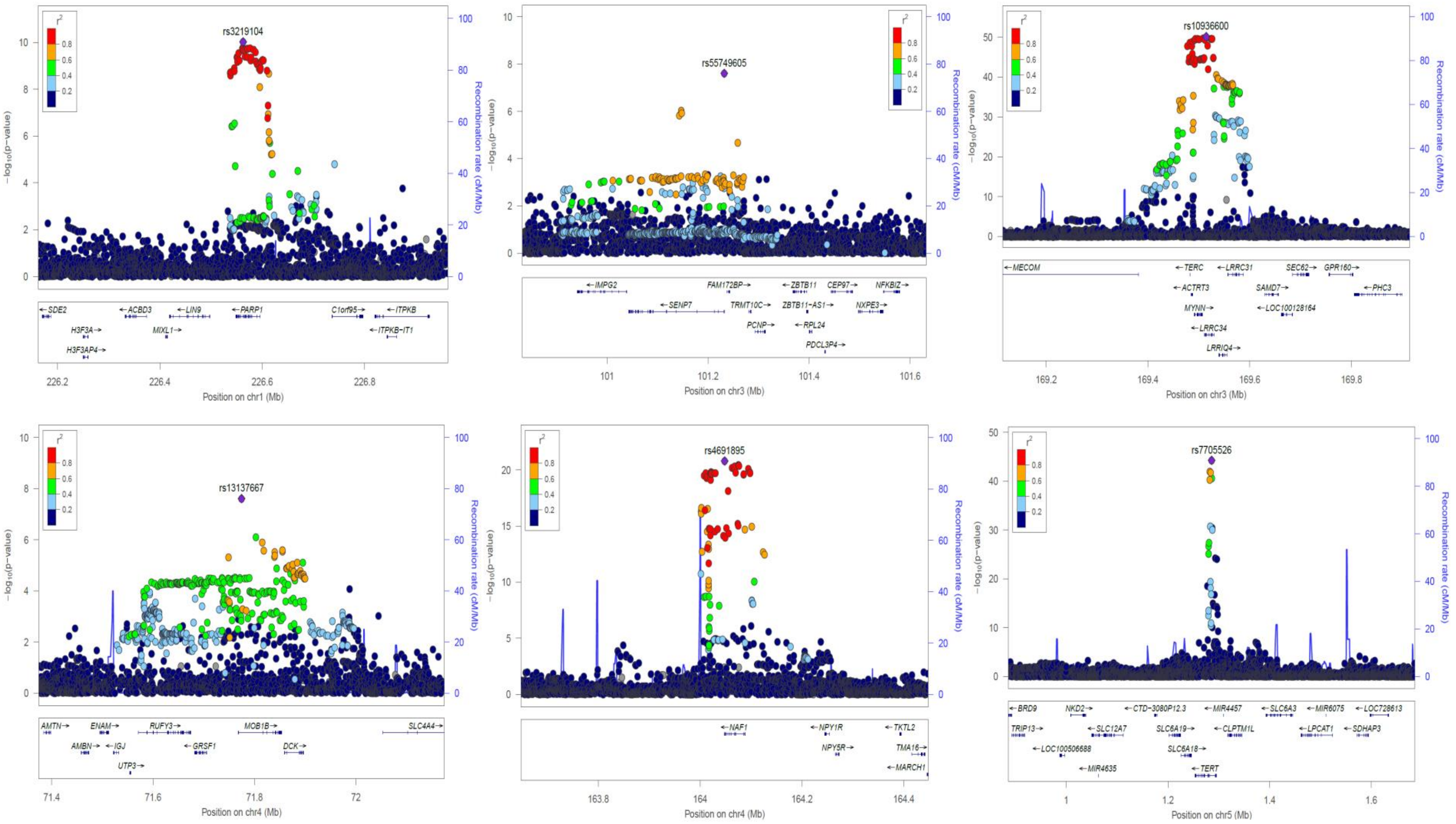

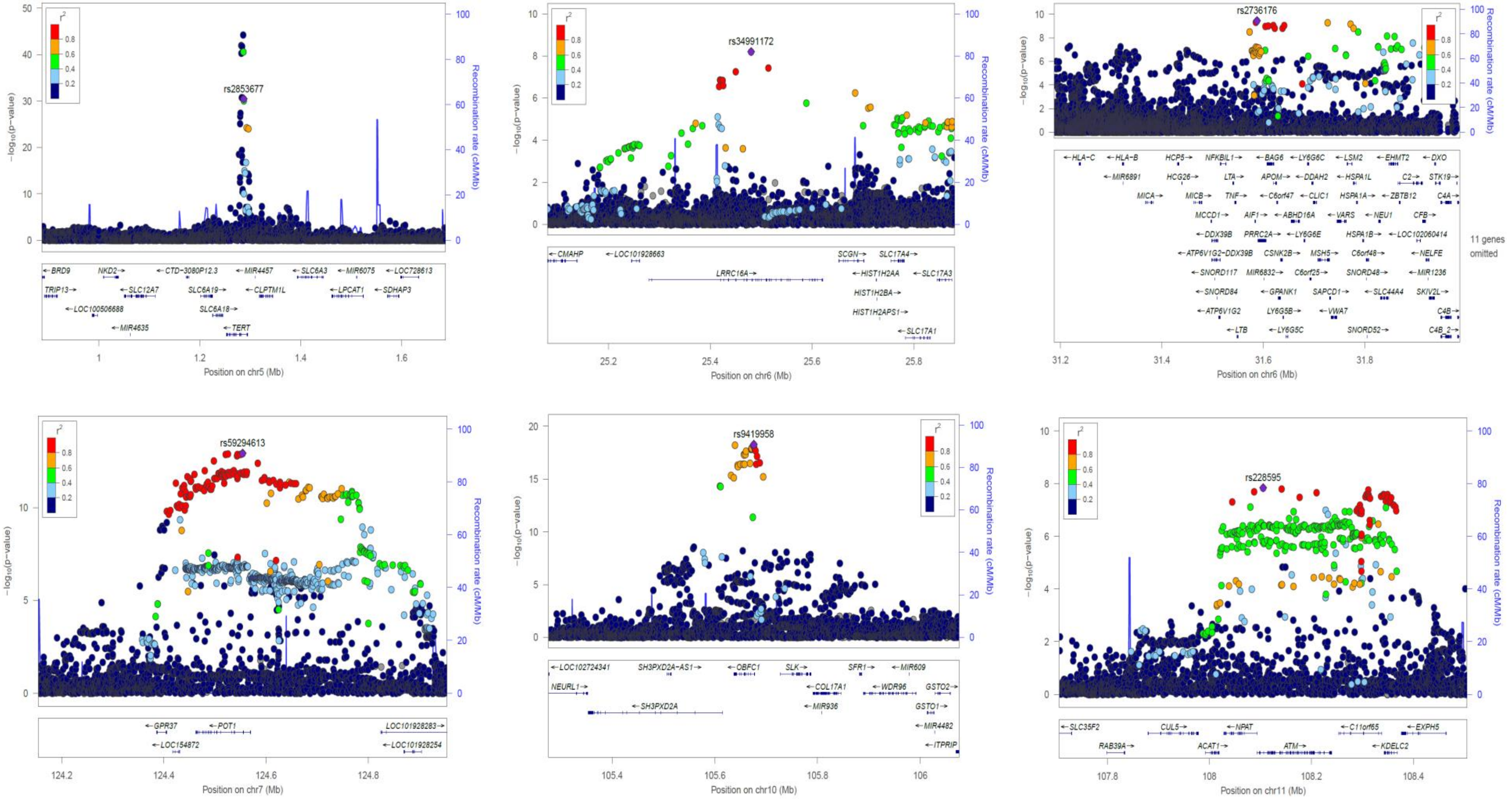

rs2853677
Position on chr5 (Mb)
rs34991172
Position on chr6 (Mb)
rs2736176
Position on chr6 (Mb)
11 genes omitted
rs59294613
Position on chr7 (Mb)
rs9419958
Position on chr10 (Mb)
rs228595
Position on chr11 (Mb)
Recombination rate (cM/Mb)

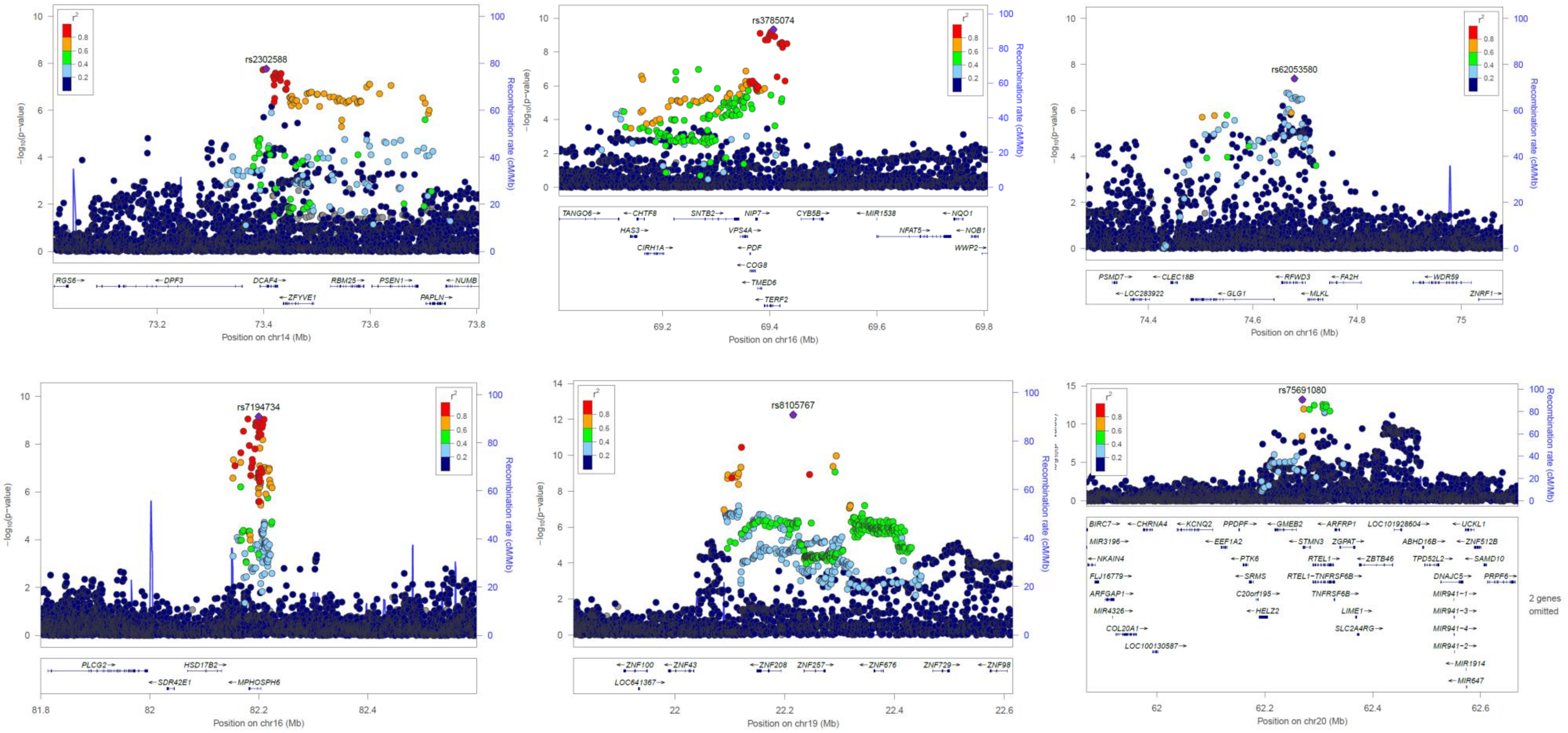
rs2302588
Position on chr14 (Mb)
rs3785074
Position on chr16 (Mb)
rs62053580
Position on chr16 (Mb)
rs7194734
Position on chr16 (Mb)
rs8105767
Position on chr19 (Mb)
rs75691080
Position on chr20 (Mb)
Recombination rate (cM/Mb)
2 genes omitted

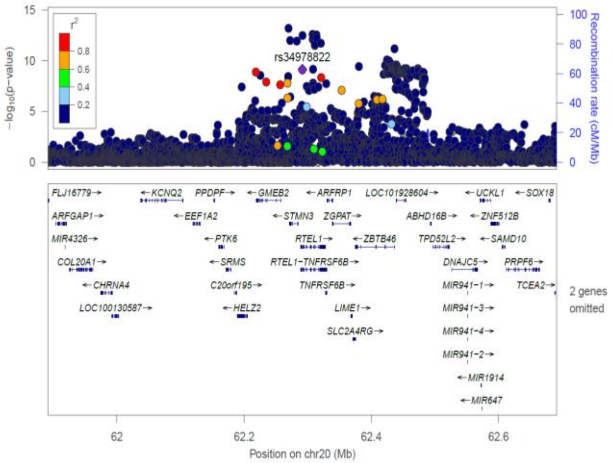
rs34978822
$-\log_{10}(p\text{-value})$
Recombination rate (cM/Mb)
r^2
0.8
0.6
0.4
0.2
FLJ16779→
←KCNQ2
PPDPF→
←GMEB2
←ARFRP1
LOC101928604→
←UCKL1
←SOX18
ARFGAP1→
←EEF1A2
←STMN3
ZGPAT→
ABHD16B→
←ZNF512B
MIR4326→
←PTK6
RTEL1→
←ZBTB46
TPD52L2→
←SAMD10
COL20A1→
←SRMS
RTEL1−TNFRSF6B→
DNAJC5→
PRPF6→
←CHRNA4
C20orf195→
TNFRSF6B→
MIR941−1→
TCEA2→
LOC100130587→
←HELZ2
LIME1→
MIR941−3→
SLC2A4RG→
MIR941−4→
MIR941−2→
←MIR1914
←MIR647
2 genes omitted
Position on chr20 (Mb)

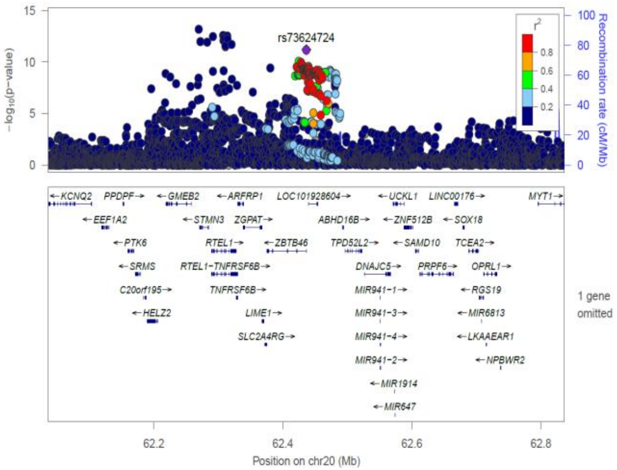
rs73624724
$-\log_{10}(p\text{-value})$
Recombination rate (cM/Mb)
r^2
0.8
0.6
0.4
0.2
←KCNQ2
PPDPF→
←GMEB2
←ARFRP1
LOC101928604→
←UCKL1
LINC00176→
MYT1→
←EEF1A2
←STMN3
ZGPAT→
ABHD16B→
←ZNF512B
←SOX18
←PTK6
RTEL1→
←ZBTB46
TPD52L2→
←SAMD10
TCEA2→
←SRMS
RTEL1−TNFRSF6B→
DNAJC5→
PRPF6→
OPRL1→
C20orf195→
TNFRSF6B→
MIR941−1→
←RGS19
←HELZ2
LIME1→
MIR941−3→
←MIR6813
SLC2A4RG→
MIR941−4→
←LKAAEAR1
MIR941−2→
←NPBWR2
←MIR1914
←MIR647
1 gene omitted
Position on chr20 (Mb)

Supplementary Figure 4. Mendelian randomisation results for the effect of shorter LTL on the risk of 122 diseases in UK Biobank. MR analysis was repeating using only SNPs that reached genome-wide significance ($P<5x10^{-8}$). Data shown are odds ratios and 95% confidence intervals for a 1 standard deviation shorter LTL. Diseases are classified into groups as indicated by the boxing and sorted alphabetically within disease group. Nominally significant (P<0.05) associations estimated via inverse-variance weighted Mendelian randomisation are shown in green for a reduction in risk and purple for an increase in risk due to shorter LTL. Where ^{O} indicates nominal (P<0.05) evidence of pleiotropy estimated by MR-Eggers intercept. Full results are also shown in Table S16 along with the full MR sensitivity analysis.

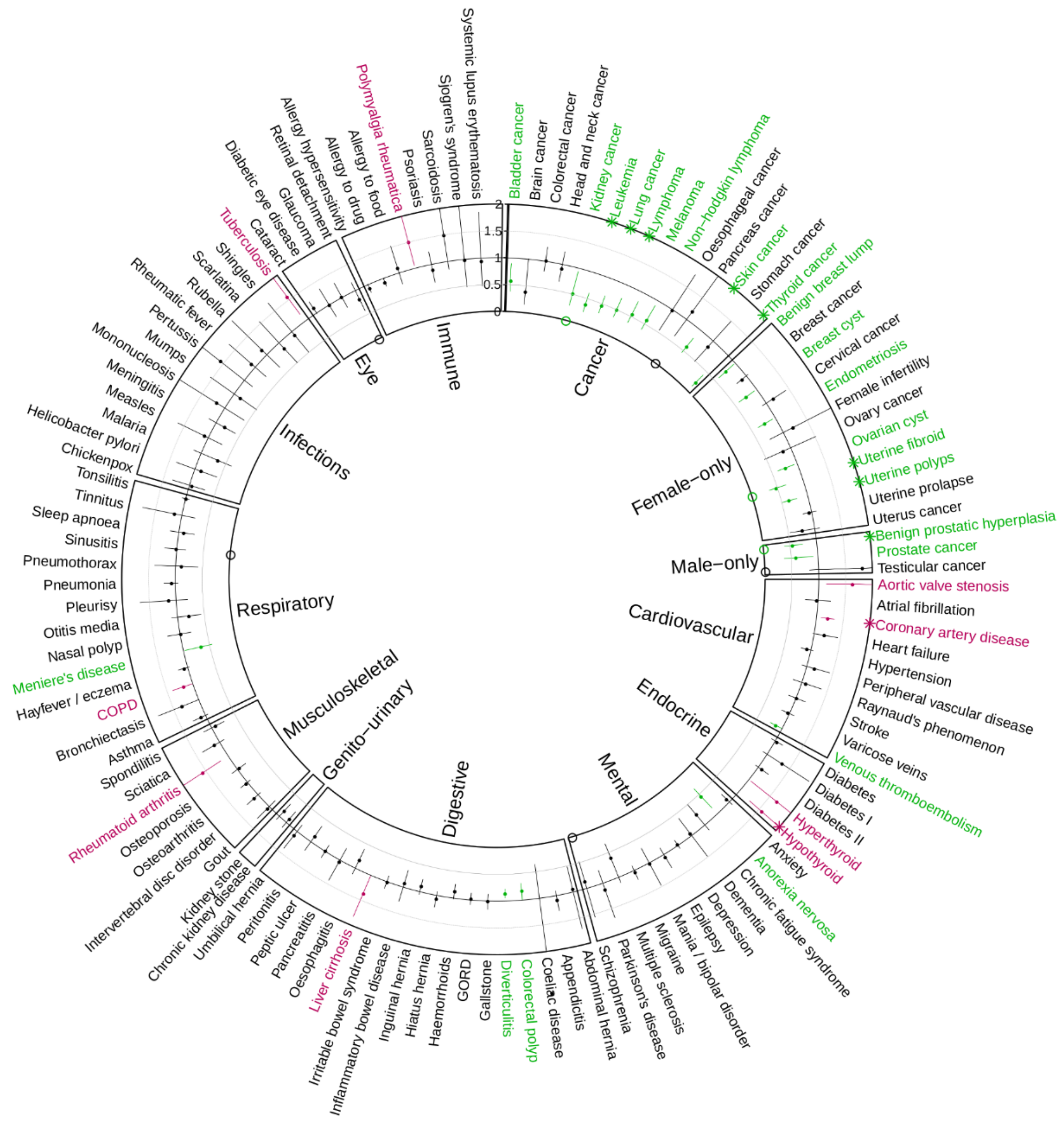

Supplemental Methods

Information on study cohorts

The demographic characteristics of all study cohorts, for both discovery and replication phases are shown in Table S1. All individuals included in the analysis are of European descent.

ENGAGE

The majority of the studies included have previously been described[1]. In addition to these the following studies were included in this analysis.

GENMETS

GENMETS is a subcohort of the Finnish population-based Health 2000 study, comprising of metablic syndrome cases and controls. This cohort is described in more detail elsewhere[2].

NESDA

The Netherlands Study of Depression and Anxiety (NESDA) is an ongoing cohort study into the long- term course and consequences of depressive and anxiety disorders. A description of the study rationale, design, and methods is given elsewhere[3]. Briefly, in 2004 to 2007, participants aged 18 to 65 years were recruited from the community (19%), general practice (54%), and secondary mental health care (27%), therefore reflecting various settings and developmental stages of psychopathology to obtain a full and generalizable picture of the course of psychiatric disorders. A total of 2981 participants were included, consisting of persons with a current or past depressive and/or anxiety disorder and healthy control subjects. Exclusion criteria were a clinically overt primary diagnosis of psychotic, obsessive compulsive, bipolar, or severe addiction disorder and not being fluent in Dutch. The research protocol was approved by the ethical committee of participating universities, and all respondents provided written informed consent.

ROTTERDAM

The Rotterdam Study is a population-based cohort study that investigates the occurrence and determinants of diseases in the elderly, which has been ongoing since 1990[4]. As of 2008, detailed phenotypic and genetic data has been collected on ~15,000 subjects aged 45 years or over. For this study the RS-I and RS-III cohorts were used. The Medical Ethics Committee at Erasmus Medical Center approved the study protocol.

EPIC-InterAct case-cohort study

The EPIC-InterAct study aimed to investigate the independent and interactive effects of genetic and behavioural risk factors on type 2 diabetes risk[5,6]. EPIC-InterAct is a case-cohort study nested within 8 of the 10 countries participating in the EPIC-Europe cohort study. EPIC-InterAct ascertained 12,403 cases of type 2 diabetes from a total cohort of 340,234 participants who provided blood samples at baseline and were followed-up for an average of 7 years (~4 million years of follow-up. Cases were ascertained from multiple data sources including self-report of a physician diagnosis of diabetes, linkage to primary/secondary care records, medication use, hospital admission data and death registration data. We also established a random sub-cohort of 16,154 participants who were representative of participants within each country. By design there is an overlap with the set of incident diabetes cases (n=778). Participant characteristics have been previously reported in detail[5,6]. Observational statistics of LTL, genotyping and imputation are summarised in the Table S1 and Table S2.

EPIC-CVD case-cohort study

EPIC-CVD was designed as a case-cohort study that uses the same random sub-cohort as InterAct, with a focus on incident coronary heart disease and stroke events[7]. The participants included in this analysis are thus incident cases only (7722 coronary heart disease cases and 3451 cerebrovascular disease cases). We also included an additional 752 participants as a random sub-cohort from the two countries not included in EPIC-InterAct (Greece and Norway). Detailed characteristics of the EPIC-CVD participants has been previously reported[8].

Telomere length measurements

Telomere length measurements were performed using an established quantitative PCR technique[9] across 6 laboratories. Laboratory specific information is given below and in Table S1. Details of the techniques used within Helsinki, Leicester and London have been given elsewhere[1].

NESDA: Fasting blood was drawn from participants in the morning between 8:30 and 9:30 am and blood samples were stored in a -80°C freezer afterwards. Leukocyte TL was determined at the laboratory of Telomere Diagnostics, Inc. (Menlo Park, CA, USA), using quantitative polymerase chain reaction (qPCR), adapted from the published original method[9]. Telomere sequence copy number in each patient's sample (T) was compared to a single-copy gene copy number (S), relative to a reference sample. The detailed method is described elsewhere[10].

Rotterdam: Telomere length was measured using a qPCR assay based on the method described elsewhere[9] with minor modifications. For each sample the telomere and 36B4 assay were run in separate wells but in the same 384 wells PCR plate. Each reaction contained 5 ng DNA, 1 uM of each of the telomere primers (tel1b-forward: GGTTTGTTTGGGTTTGGGTTTGGGTTTGGGTTTGGGTT, tel2b-reverse: GGCTTGCCTTACCCTTACCCTTACCCTTACCCTTACCCT) or 250 nM of the 34B4 primers (36B4u-forward: CAGCAAGTGGGAAGGTGTAATCC, 36B4d-reverse:

CCCATTCTATCATCAACGGGTACAA) and 1x Quantifast SYBR green PCR Mastermix (Qiagen). The reactions for both assays were performed in duplicate for each sample in a 7900HT machine (Applied Biosystems). Ct values and PCR efficiencies were calculated per plate using the MINER algorithm[11]. Duplicate Ct values that had a Coefficient of Variance (CV) of more than 1% were excluded from further analysis. Using the average Ct value per sample and the average PCR efficiency per plate the samples were quantified using the formula Q=1/(1+PCR eff)^Ct. The relative telomere length was calculated by dividing the Q of the telomere assay by the Q of the 34B4 assay. To validate the assay 96 random samples were run twice and the CV of that experiment was 4.5%.

Cambridge: Relative mean LTL was measured using a ViiATM Real-Time quantitative PCR system (ThermoFisher Sicentific, Inc), and expressed as a ratio (T/S) of the relative quantities of the telomeric TTAGGG repeat (T) and the single copy of a housekeeping gene, *Albumin* (S). The denominator determines total genome copies per sample, controlling the technical errors during quantification. The measurement was validated by the Terminal Restriction Fragment (TRF) analysis (the “gold standard” measurement of TL) using separate DNA samples extracted from peripheral blood mononuclear cells in 30 individuals (Pearson’s r =0.69). Batch effect was corrected by normalising all the other batches to the fourth batch. Each sample was measured repetitively for three times within one batch, when the same sample was measured in more than one batch, measurement from the last batch was kept for the sample. Samples with coefficients of variation greater than 10% were excluded.

Description of Individual loci associated with LTL

Chr1p13.2. The lead SNP (rs12065882) and three high LD variants are all located within introns of *MAGI3* (*membrane associated guanylate kinase, WW and PDZ domain containing 3*). *MAGI3* has been proposed to act as a tumour suppressor; it regulates cell proliferation in glioma via wnt/β-catenin signalling and interacts with PTEN[12,13]. Both S-PrediXcan and COLOC analyses give evidence to support expression of *AP4B1* (*adaptor related protein complex 4 subunit beta 1*) being influenced by the associated variants. This gene encodes a subunit of a heterotetrametric adapter-like complex 4 that involves in Golgi-associated and lysosomal vesicle biogenesis and membrane trafficking, transporting proteins from the trans-Golgi network to the endosomal-lysosomal system[14,15]. Mutations in this gene are associated with an autosomal recessively inherited disease, spastic paraplegia type 47[16]. There is also evidence of a colocalised eQTL signal for *PTPN22* (*protein tyrosine phosphatase, non-receptor type 22*) in three tissues. PTPN22 interacts with the proto-oncogene CBL, a member of the E3 ubiquitin ligase family that has been implicated in several cancers.

Chr1q24.2. rs35675808 is located downstream of the 3' UTR of *CD247 (CD247 molecule),* which encodes T-cell receptor zeta that constitutes the T-cell receptor-CD3 complex, coupling antigen recognition to several signalling transduction pathways, essential in adaptive immune response[17,18]. Pathways that have been shown to be implicated with this gene include HIV life cycle and translocation of ZAP-70 to immunological synapse (Reactome). Mutations in this gene are associated with autosomal recessive immunodeficiency 25 (IMD25 [MIM: 610163]), characterised by T-cells impaired response to alloantigens, tetanus toxoid and mitogens. Another gene, the *POU2F1* (*POU class 2 homeobox 1*), located 3kb upstream of this variant, might be biologically relevant. This gene, also known as the *OCT1*, belongs to the first identified members of the POU transcription factor family[19,20]. Members of this family contain the POU domain, a 160-amino acid region necessary for DNA binding to the octameric motif (5'-ATGCAAAT-3')[19]. POU2F1, as a transcriptional factor, is involved in cell cycle regulation and transcription of histone H2B and other cellular housekeeping genes[20,21]. It has also been suggested that the expression of histone H2B was downregulated in response to double-stranded DNA breaks via a mechanism that modulates transcriptional regulatory potential of POU2F1 by site-specific phosphorylation[22]. POU2F1 is implicated with various pathways, including the RNA Polymerase III transcription initiation, cytokine signalling in immune system, BRCA1 pathway and glucocorticoid receptor signalling (Reactome). This gene also facilitates human herpes simplex virus (HSV) infection by forming a multiprotein-DNA complex with the virion proteins, activating transcription of the viral immediate early genes[23].

Chr1q42.12. Variants at this locus are focused across the *PARP1* gene, which encodes the first protein member of the poly(ADP-ribosyl)transferases family, also termed as the ADP-ribosyltransferases with diphtheria toxin homology (ARTDs). It plays an essential role in various pathways of DNA repair and chromatin remodelling, including single- and double-strand break repair, nucleotide excision repair, stabilization of replication forks, and modulation of chromatin structure, thereby maintaining genomic integrity and stability[24]. Because the DNA double-strand breaks structurally resemble telomeres, regulators and components of DNA repair machinery have been shown to be implicated in telomere homeostasis[25]. Of note, rs1136410 (r^2=1.0 to the lead) causes a known V762A substitution in

PARP1 (poly(ADP-ribose) polymerase 1), which has been shown to reduce PARP1 activity. The allele that reduces activity is associated with shorter LTL, consistent with previous studies where knockdown of PARP1 leads to telomere shortening. PARP1 was identified as a telomeric double-stranded repeats binding factor in a proteomic study of telomeres using DNA *in situ* hybridization in conjugation with mass spectrometry[26]. In addition to the coding change there is also eQTL evidence for *PARP1* (S-PrediXcan and COLOC, online methods, Table S7) in pancreas, with the shorter LTL allele associating with reduced *PARP1* expression. Another SNP, rs907187, is highlighted in the integrated analysis of non-coding variants and is located within the 5' UTR of *PARP1*, which could mediate the effect on gene expression.

Chr2p16.2. rs754017156 is located within intron 3 of *ACYP2* (*acylphosphatase 2*) and also causes an in-frame insertion of two amino acids into *TSPYL6* (*TSPY like 6*). This gene encodes a nuclear protein, the Testis-Specific Y-Encoded-Like Protein 6, that involves in the nucleosome assembly. Biological function of this protein is largely unexplored. Studies have associated genetic polymorphisms of this gene region with increased risk of ischemic stroke[27], and breast cancer in the Han Chinese population[28]. There are no high LD SNPs, but an evidence of an eQTL in testis for *TSPYL6*.

Chr2q34. rs56810761 is located within intron 7 of *UNC80* (*unc-80 homolog, NALCN channel complex subunit, A*) gene. There are no high LD SNPs, but an evidence of an eQTL for *SNAI1P1* (*snail family zinc finger 1 pseudogene 1*) in testis in the co-localisation analysis. *SNAIP1* is a processed pseudogene of *SNAI1*, which encodes the human ortholog of a zinc finger protein of the snail family, first cloned in *Drosophila*, which was demonstrated to be essential in the formation of mesoderm during gastrulation and embryonic development[29].

Chr3q12.3. This locus consists of a 77 SNPs located predominantly across *SENP7* (*SUMO1/sentrin specific peptidase 7*) gene. The lead SNP is located 53bp upstream of *SENP7* within a proximal promoter. It is associated with a DNaseI sensitivity QTL and with *SENP7* expression in one tissue (co-localisation). Lower expression of *SENP7* associates with shorter LTL. Although it has no known role in telomere regulation, the small ubiquitin-like modifier (SUMO) functions as a post-translational modification, regulating various biological events, especially in DNA repair, chromatin organization, transcription, and RNA metabolism[30], which are essential biological events pertinent to telomere homeostasis.

Chr3q13.2. The variants in this region are all located within intron 2 of a predicted mRNA, *RP11-572M11.4* and downstream of a non-coding RNA *RP11-572M11.3* (also named *LINC02044*). There is no supporting evidence to suggest which gene is potentially influenced at this locus.

Chr3q26.2. This locus contains 47 SNPs in high LD ($r^2 < 0.8$) with the lead SNP (rs1093660). The *telomerase RNA component* (*TERC*) is the functional candidate in this locus. One SNP (rs2293607, r^2=0.81 to rs1093660) is located 63bp downstream of the *TERC* sequence, which potentially leads to altered *TERC* expression[31]. However, the lead variant, rs10936600, encodes a L241I substitution within *LRRC34* (*Leucine rich repeat containing 34*), which is predicted to be deleterious (Table S6). The CADD score (19.81) places this SNP just outside of the 1% most deleterious mutations. LRRC34 is a member of the leucine rich repeat containing protein family. Although little is known about its biological function, it has been suggested to

be implicated in the maintenance and regulation of pluripotency[32]. Knock down of LRRC34 results in reduced expression of some, but not all, pluripotency genes[32]. As genes encoding the telomerase enzyme share the same expression patterns as those of the pluripotency genes, thereby they are potentially subjected to the LRRC34-mediated transcriptional regulation. Another highly linked variant, rs10936599 (r^2=1.0) is predicted to have a functional effect in the integrated analysis of non-coding variants (Table S7). It is located on the edge of the active promoter region of *MYNN*, just inside the coding sequence. An eQTL is observed for *MYNN* in testis (shorter TL associated with higher expression), suggesting that this SNP may alter *MYNN* expression. MYNN protein is a member of the BTB/POZ and zinc finger containing family that is involved in transcriptional regulation. It has also been shown to interact with CUL3, a core component of the E3 Ubiquitin ligase complex, which functions in many cellular processes including DNA repair. LTL variants at this locus have been associated with idiopathic pulmonary fibrosis, of which telomere dysregulation is attributed to the disease aetiology[33]. Despite the obvious involvement of *TERC* in telomere length regulation, little bioinformatic evidence is available to support it to be the only likely-causal gene in this region, i.e. other candidate genes might also explain the locus association, such as *LRRC34* and *MYNN*. However, it is also possible that with *TERC* being a processed non-coding RNA, the relevant information is limited in standard datasets. There are no eQTLs for *TERC* in the GTex dataset, but a study has shown that variants in the regulatory region can affect its expression level, possibly by facilitating the maturation of *TERC* via 3' processing[31].

Chr4q13.3. The lead variant rs13137667 is located within the first intron of *MOB1B* (*MOB kinase activator 1B*). There are 49 variants in high LD, the majority of which are located intronically within *MOB1B* or *DCK* (*deoxycytidine kinase*). No high LD non-synonymous variants or co-localised eQTLs were found at this locus. MOB (Mps one binder) was originally identified as an Mps1 binding protein in yeast, regulating mitotic checkpoint and cytokinesis, and is evolutionarily conserved across all major kingdoms[34]. Human MOB1B homolog activates LATS1/2 (Large tumour suppressor 1/2) through protein-protein interaction in the Hippo signalling pathway, resulting in the inhibition of cell proliferation, apoptosis, and thus tumour suppression[35]. DCK is a key component of the deoxyribonucleoside salvage pathway and phosphorylates deoxycytidine, deoxyguanosine and deoxyadenosine to dCMP, dGMP and dAMP respectively.

Chr4q31.23. There are 65 associated variants clustered towards the 5' end of *DCLK2* (*doublecortin like kinase 2*). There is an eQTL co-localised with *DCLK2* in one tissue (Table S7). *DCLK2* encodes a protein that contains four independent functional domains: two doublecortin domains at the N-terminus, essential for microtubule binding and regulating microtubule polymerisation, a serine/threonine protein kinase domain at the C-terminus, sharing substantial homology to Ca^{2+}/calmodulin-dependent protein kinase, and a serine/proline-rich domain in between the two termini, which mediates multiple protein-protein interactions. Mouse models with single or double copies of *Dclk2* gene ablated are viable and fertile, however, a simultaneous deletion of *Dcx* gene, encoding another protein member of the doublecortin family, results in spontaneous seizures, hippocampal disorganisation and poor survival[36], phenotypically mimicking human lissencephaly, X-linked, 1 disease (LISX1 [MIM: 300067]).

Chr4q32.2. This locus contains 70 closely related (r^2>0.8) SNPs spanning *NAF1* (*nuclear assembly factor 1 ribonucleoprotein*), a gene encoding an RNA-binding protein, required for the synthesis of box H/ACA RNAs and sequential assembly with proteins to form ribonucleoprotein (RNP) complex. The box H/ACA RNPs regulates three fundamental cellular processes: protein synthesis, mRNA splicing via site-specific pseudouridylation of ribosomal RNAs and small nuclear RNAs and telomere maintenance by facilitating the maturation of *TERC* in telomerase[37]. Expression evidence was found for *NAF1* (S-PrediXcan and COLOC) and an antisense transcript *RP11-563E2.2* (COLOC, online methods, Table S7). The lead SNP, rs4691895, is a non-synonymous variant in *NAF1* (L368V) along with another high LD variant (rs4691896, r^2=1, I162V). Individually both are predicted to be benign; however, it is unclear what effects they may have in combination.

Chr5p15.33. There are two independently associated SNPs at this locus, neither of which have any high LD variants. Both SNPs are located within intron 2 of *TERT*, but little functional evidence was found to support their involvements in regulating *TERT* levels, which might be due to the transcriptional repression of *TERT* in most somatic tissues.

Chr5q14.1. The lead variant, rs62365174, is located in intron 4 of *TENT2* (*terminal nucleotidyltransferase 2*, previously named *PAPD4 and GLD2*). There are 137 SNPs in high LD (r^2<0.8), which fall across the region of *TENT2* and include upstream, intronic and 3' UTR variants. There is strong evidence that these variants can affect the expression of *TENT2*, with eQTLs co-localised in 9 tissues, exhibiting consistent positive correlations, i.e. reduced expression associates with decreased LTL. TENT2 functions as the cytoplasmic poly(A) RNA polymerase that adds successive AMP monomers to the 3'-end of specific RNAs, forming a poly(A) tail, exhibiting strict substrate specificity, that, different from the canonical nuclear poly(A) RNA polymerase, only functions on cytoplasmic RNAs[38]. Previous studies have suggested its role in the polyadenylation and stability of p53 mRNA[39] and several miRNAs[40].

Chr5q31.2. The associated variant, rs112347796, has no further variants in high LD (r^2>0.8). It is located within intron 1 of *UBE2D2* (*ubiquitin conjugating enzyme E2 D2*), which is involved in the DNA damage repair[41]. There is no evidence to suggest the potential function of this variant.

Chr6p22.2. This locus contains 10 SNPs in high LD (r^2>0.8) with the lead SNP, all located around *CARMIL1* (*capping protein regulator and myosin 1 linker 1*, previously named *LRR16A*). One SNP, rs913455, causes a synonymous change within exon 3 and has scored to have possible regulatory function (Table S8), which may be driven in part by its high conservation and location within the coding region. There is no supporting literature evidence to identify which gene(s) may be influenced at this locus.

Chr6p21.33. There are 11 SNPs in high LD (r^2>0.8) with the lead SNP, which are located across the major histocompatibility complex (MHC) class III region. MHC is a highly polymorphic and gene-dense region with complex linkage disequilibrium structure, and thus characterisation of potential causal genes within this region is difficult. A number of genes can potentially serve as causal gene candidates, including *PRRC2A*, *CSNK2B* and *BAG6*. There is evidence that the expression of both *BAG6* and *CSNK2B* (S-PrediXcan and COLOC, Table S7) is affected. The lead variant is located upstream of *PRRC2A*, which was previously known as the *BAT2* (*HLA-B*

associated transcript 2) gene, encoding a large protein (2157 amino acids). PRRC2A has been shown to be involved in the pre-mRNA editing, as spliceosome and splicing regulators were found to be able to bind to the PRRC2A in protein-protein interaction assays, including the heterogeneous nuclear RNPs and the cleavage and polyadenylation specific factor 1[42]. As maturation of the telomerase RNA subunit involves a spliceosome-mediated single cleavage reaction[43], PRRC2A may regulate telomere length via involvement in the biogenesis of *TERC*. Of note, another variant, rs805299 (r^2=1), located within intron 1 of *BAG6* (*BCL2 associated athanogene 6*), shows a high probability for promoter activity and is predicted to have regulatory function in the integrated analysis of non-coding variants (Table S8). *BAG6* was part of a cluster of genes that encode a multifunctional protein, involved in various pathways, including intracellular protein quality controls by promoting proteasomal degradation of misfolded and mislocalised proteins, and DNA damage-induced apoptosis. Another variant, rs5872 (r^2=1), is located within the 3'UTR of *CSNK2B* (*casein kinase 2 beta*). CSNK2B is a subunit of CSNK2 that is involved in multiple pathways but of note has been shown to interact with TRF1. CSNK2-mediated phosphorylation of TRF1 is required for the binding of TRF1 to telomeres, which has been proposed to be essential for telomere length homeostasis[44].

Chr7q31.33. The associated variants cover the *POT1* (*protection of telomeres 1*) gene, which encodes the most conserved protein component of the shelterin complex among all eukaryotes[45]. It is tethered to the TERF1 and TERF2 homodimers via a TIN2-mediated linkage, and specifically bound to the single-stranded telomeric repeats, protecting it from nucleolytic degradation[46]. Moreover, POT1 controls the sequence precision at the 5' ends, which are identical among nearly all human chromosomes, and regulates telomere length by restricting telomerase binding[47]. Rare nonsense mutations within this gene, which blocked physical interactions of POT1 with telomeric single-stranded repeats and other components of the shelterin protein complex, were identified by whole-exome sequencing in families with strong histories of chronic lymphocytic leukaemia[48]. The integrated analysis of non-coding variants highlights rs2239532 (r^2=0.85), located within the 5'UTR of *GPR37* (*G protein-coupled receptor 37*), as having regulatory function (Table S8). Although no direct eQTL evidence is available to support POT1, there is evidence to link the expression of an uncharacterised *POT1-AS* transcript (*RP11-3B12.1*) to LTL via co-localisation in two tissues (Table S8).

Chr8p23.2. This region contains 52 SNPs in high LD (r^2<0.8) and is located within 3 introns towards the 3' end of *CSMD1* (*CUB and Sushi multiple domains 1*) gene. *CSMD1* was potentially associated with a rare neurological disease, the benign adult familial myoclonic epilepsy[49]. It may also act as a suppressor of squamous cell carcinomas, yet unequivocal evidence is lacking[50,51]. The gene-knockout mouse was used as a schizophrenia human disease model, exhibiting increased levels of exploratory activity, behavioural despair anxiety-related response, and decreased startle reflex (MGI: 3528558). However, no direct supporting evidence is available to suggest CSMD1 or other genes as causal gene candidates in this region.

Chr8q22.2. Four SNPs are located upstream of *COX6C* (*cytochrome c oxidase subunit 6C*). COX6C is a subunit of complex IV that catalyses the final step of the mitochondrial respiratory chain[52]. No functional data is available to pinpoint causal genes for this locus.

Chr10p15.1. The 6 associated variants (in LD, r^2>0.8) at this locus are clustered within the first intron of *ASB13* (*ankyrin repeat and SOCS box containing 13*), a member of the suppressor of cytokine signalling box protein superfamily. Members of this protein family can also be components of E3 ubiquitin ligase complexes[53]. No causal gene candidates can be prioritised for this locus.

Chr10q24.33. This region contains *STN1* (*STN1, CST complex subunit*, also termed *OBFC1* in humans), a component of the telomere binding CST complex. There is strong evidence that the variants affect *STN1*(*OBFC1*) expressions across multiple tissues (S-PrediXcan and COLOC, Table S7). The CST complex regulates telomere maintenance by mediating the access to telomeres for telomerase and DNA polymerase α[54].

Chr11q21. The lead variant, rs117037102, is located within intron 5 of *CEP295* (*centrosomal protein 295*, also termed *KIAA1731*). There is a potentially damaging protein coding variant (rs117405490, r^2=1), which results in a P to A substitution at position 783 of CEP295. CEP295 is a centriole-enriched microtubule-binding protein, highly conserved across species and involved in centriole biogenesis, essential for cell cycle regulation and mitotic progression[55].

Chr11q22.3. The associated variants fall across a ~321kb region which includes several genes, including *ATM* (*ATM serine/threonine kinase*), encoding a protein kinase that phosphorylates many checkpoint-determining and regulatory proteins, such as p53, Chk2 and BRCA1, and thus playing an essential role in cell cycle control and DNA-damage-activated signalling pathways[56]. ATM is responsible for the human genetic disorder ataxia telangiectasia (AT [MIM: 208900]), manifested with genome instability, cerebellar and thymic degeneration, immunodeficiency, premature ageing, sensitivity to ionizing radiation and predisposition to cancer[57]. There are eQTLs supporting *ATM* and another gene, *ACAT1* (*acetyl-CoA acetyltransferase 1*), within the region. ACAT1 is a mitochondrial protein, expression levels of which have been linked to some cancers[58]. Defects in this gene are associated with 3-ketothiolase deficiency, an inborn error of isoleucine catabolism[59].

Chr12p13.1. The lead variant and 2 in high LD (r^2<0.8) are located upstream of *ATF7IP* (*activating transcription factor 7 interacting protein*), also named *MCAF1*, actively involved in histone modification, chromatin organisation, and Sp1-dependent maintenance of telomerase activity in cancer cells[60]. It was previously shown to regulate expression of both *TERT* and *TERC* and consequently telomerase activity[61].

Chr12q13.13. There are 7 variants in high LD (r^2 <0.8), located within a 3kb region upstream of *SMUG1* (*single-strand-selective monofunctional uracil-DNA glycosylase 1*), a gene involved in base-excision repair. Although there is no bioinformatic evidence to show that these variants affect SMUG1 expression levels, previous functional studies have suggested that SMUG1 might influence telomere length by interacting with the telomerase component Dyskerin (DKC1) with which it controls rRNA processing[62].

Chr14q24.2. The lead variant is a non-synonymous (W22C) variant in *DCAF4* (*DDB1 and CUL4 associated factor 4*). Another variant in high LD (rs3815460, r^2=1) also causes a protein coding change (S345C). Both variants are predicted to be damaging individually. DCAF4 interacts with the Cul4-Ddb1 E3 ubiquitin ligase macromolecular complex, which regulates processes

including DNA repair and cellular proliferation[63]. *DDB* (*DNA damage binding protein*) is highly expressed in multipotent hematopoietic progenitors, conditional ablation of which in hematopoietic stem and progenitor cells led to a complete loss of pluripotency and self-renewal of progenitors and stem cells, suggesting its role in cell differentiation, apoptosis and death[64]. An intronic G-to-A variant (rs2535913) has been associated with shorter LTL[65]. A further SNP, rs2286838 (r^2=0.9) causes a coding change in *ZFYVE1* (*zinc finger FYVE-type containing 1*, S408R), which also has a predicted damaging effect. This protein, also known as the *double FYVE-containing protein 1* (*DFCP1*), contains two zinc-binding FYVE domains in tandem, which has been shown to be localised on endoplasmic reticulum and Golgi apparatus via binding to phosphatidylinositol 3-phosphate containing membranes, essential for the regulation of autophagy[66].

Chr14q24.3. The lead variant, rs59192843, is located within intron 6 of *BBOF1* (*basal body orientation factor 1*, also termed as *CCDC176*). There are no coding variants or eQTLs associated with the lead variant. Two variants in high LD (r^2<0.8), rs73301475 and rs17094157 scored highly in the integrated analysis of non-coding variants (Table S8). These are located within an enhancer of *ENTPD5* (*ectonucleoside triphosphate diphosphohydrolase 5*) and the 3' UTR of *COQ6* (*coenzyme Q6, monooxygenase*), respectively. ENTPD5 hydrolyses UDP to UMP to promote protein N-glycosylation and folding. It has been shown that ENTPD5 was upregulated in cell lines and primary human tumour samples with active AKT, promoting cell growth and survival[67]. AKT activation also contributes to the elevation of aerobic glycolysis seen in tumour cells, known as the Warburg effect. Of note, ENTPD5 was also involved in stimulating glycolysis by providing substrates for cytidine monophosphate kinase-1 that converts UMP to UDP using a phosphate molecule generated during the ATP hydrolysis cycle[68]. COQ6 is an evolutionarily conserved monooxygenase, belonging to the ubiH/COQ6 family, which is required for the biosynthesis of coenzyme Q10 (or ubiquinone), an essential component of the mitochondrial electron transport chain and one of the most potent lipophilic antioxidants implicated in the protection of cell damage by reactive oxygen species. Gene-ablated mouse model showed abnormal embryo size and growth retardation (MGI: 5548683). Mutations in this gene are associated with autosomal recessive coenzyme Q10 deficiency-6, which manifests as nephrotic syndrome with sensorineural deafness[69].

Chr14q32.11. In this locus the variants are focused across *CALM1* (*calmodulin 1*). There is an eQTL co-localised with *CALM1* expression in testis. Two SNPs (rs12885713 and rs2300496) are within the *CALM1* promoter/enhancer region and predicted to have regulatory function. *CALM1* encodes a member of the EF-hand calcium-binding protein family, regulating a number of protein kinases and phosphatases, among which CP110, by interacting with CALM1 and centrin, regulates centrosome function and cytokinesis[70].

Chr14q32.33. The lead SNP, rs117536281, is located upstream of *CDCA4* (*cell division cycle associated 4*). *CDCA4* encodes a member of the E2F family of transcription factors, regulating spindle organization, cytokinesis and cell proliferation, which may be also involved in differentiation of hematopoietic stem cells and progenitor cell lineage[71]. There are no coding variants or eQTL data for this locus.

Chr15q14. This locus consists of two associated SNPs, rs9972513 and rs12324579, which are located in an intergenic region upstream of both *c15orf53* and *RASGRP1* (*RAS guanyl releasing*

protein 1). There are no coding variants or eQTL data for this locus. *C15orf53* is a protein coding gene with uncharacterised functions, with disputable evidence suggesting its implication with schizophrenia and bipolar disorder[72]. *RASGRP1* encodes a protein that functions as a calcium- and diacylglycerol (DAG)-regulated nucleotide exchange factor specifically activating Ras through the exchange of bound GDP for GTP. RASGRP1 contains a pair of calcium-binding EF hands and a DAG-binding domain[73]. The RASGRP1-mediated Ras activation regulates T cell proliferation, development and homeostasis[74].

Chr15q21.2. There are 17 SNPs clustered around the 5' end of *ATP8B4* (*ATPase phospholipid transporting 8B4 (putative)*). There are no coding variants or eQTL data for this locus. *ATP8B4* encodes a member of the cation transport ATPase (P-type) family and type IV subfamily, which consists of a P4-ATPase flippase complex that catalyses the hydrolysis of ATP coupled to phospholipid translocation across various membranes, playing a role in vesicle biosynthesis and lipid signalling transduction[75,76]. Deleterious rare variants within this gene have been associated with systemic sclerosis, for which the principal cause of death was pulmonary diseases, including interstitial lung disease and pulmonary arterial hypertension[77]. An intronic common variant at the distal promoter region of this gene has been reported to be associated with Alzheimer's Disease[78].

Chr15q21.3. This single variant, rs117610974 is located in an intergenic region, ~220kb downstream of the closest gene, *UNC13C* (*unc-13 homolog C*), which might be implicated with vesicle formation during exocytosis, with potential capabilities of diacylglycerol and calcium binding[79]. However, there is no evidence to suggest what role this lead variant may have.

Chr15q22.31. The lead variant, rs55710439, is located within intron 6 of *ANKDD1A* (*ankyrin repeat and death domain containing 1A*). There is an eQTL for this gene co-localised in one tissue. Little is known about the ANKDD1A protein, except that it contains an ankyrin repeat domain and a death domain, both of which function in the protein-protein interaction. A closely-related SNP (in LD, $r^2<0.8$), rs57438358, predicted to have potential functional effects, is located within the 3'UTR of *SPG21* (*SPG21, maspardin*), a gene which is mutated in mast syndrome.

Chr16p13.3. This is a single variant, rs11640926, located within intron 5 on *CACNA1H*. There is no supporting evidence to suggest the effects of this variant. *CACNA1H* encodes a protein component of the voltage-dependent calcium channel complex, a T-type calcium channel that belongs to the "low-voltage activated" group, which plays an essential role in both central neurons and cardiac nodal cells and supports calcium signalling in secretory cells and vascular smooth muscle[80,81]. It is associated with a form of familial hyperaldosteronism, clinically characterised by hypertension, elevated aldosterone levels and abnormal adrenal steroid production[82]; and another genetic rare disease, the Childhood Absence Epilepsy 6[83].

Chr16q22.1. The most significantly associated variants in this region are located within and around *TERF2* (*telomeric repeat binding factor 2*), a component of the shelterin complex. TERF2 protein directly and specifically binds to the telomeric double-stranded repeats, and by interacting with other telomeric factors forming a T-loop configuration that protects chromosome ends from disruptive end-to-end joining and ligation to exogenous DNA. Mutant

forms of this gene induced DNA fusion, such as formation of anaphase bridges and lagging or ring-like chromosomes[84,85].
There is evidence that the variants affect expression of several genes in this region, with the strongest evidence for *TERF2* (S-PrediXcan and COLOC, Table S7). Longer LTL is associated with reduced expression of *TERF2*, consistent with TERF2 being a negative regulator of telomere length[86]. One variant predicted to have a functional effect, rs9939705, is located within an enhancer region upstream of *TERF2*. There is also evidence to suggest that expression of two other genes (*COG8*, and *PDF*) are also affected by the associated variants.

Chr16q23.1. Variants at this locus show co-localisation with eQTLs for *RFWD3* (*ring finger and WD repeat domain 3*) in multiple tissues. RFWD3 is a ubiquitin ligase that interacts with and ubiquitinates replication protein A (RPA), which has been shown to be essential for DNA replication and repair. Upon replication stress, RPA was recruited to stalled replication folks and ubiquitinated by the RFWD3, an essential process for recovery and homologous recombination-mediated DNA repair[87]. RFWD3 also ubiquitinates and stabilises p53/TP53 in response to DNA damage, thereby regulating the cell cycle checkpoint[88]. This gene was also clinically attributable to the Fanconi anaemia (FA [MIM: 227650]), an autosomal recessive inheritance disease manifested with chromosomal instability, bone marrow failure, dermal pigmentary changes and predisposition to malignancies.

Chr16q23.3. The association signal at this locus is across *MPHOSPH6* (*M-phase phosphoprotein 6*). There is strong eQTL evidence (S-PrediXcan and COLOC) in multiple tissues to support the associated variants influencing MPHOSPH6 expression. MPHOSPH6 is a component of the RNA exosome, a protein complex required for the degradation of RNA molecules and is required for the 3’ processing of the 5.8S rRNA[89]. There is also evidence that MPHOSPH6 interacts with PARN (poly(A)-specific ribonuclease)[90], an important regulator of mRNA catabolism which is also required for the formation of mature *TERC* RNA[91].

Chr17q25.3. The lead variant (rs144204502) is situated within the 5’ UTR of *TK* (*thymidine kinase 1*), with evidence of regulatory functions (Table S8). There are co-localised eQTLs for *TK1* in three tissues. *TK1* encodes a cytosolic enzyme that catalyses the conversion of thymidine to dTMP, which is the first step of the salvage pathway of dTTP biosynthesis, essential for DNA replication. There are two forms of the TK enzyme, besides the TK1, TK2 catalyses the same reaction but in the mitochondria. The activity of TK1 is delicately regulated by a configurational transition, changing from dimer to tetramer upon increases in ATP and enzyme concentrations, with a consequently accompanied upregulation of catalytic efficiency[92]. This regulatory fine-tuning of TK1 activity ensured a balanced pool of nucleic acid precursors. High TK1 expression was detected in numerous types of cancers, including gastrointestinal adenocarcinomas and oesophageal and uterine squamous cell carcinomas[93].

Chr18p11.32. All variants within the locus are located within the *TYMS* (*thymidylate synthetase*) gene, either within the intronic or the 3’UTR regions. There is an eQTL for *TYMS* co-localised in one tissue. TYMS is involved in the *de novo* biosynthesis of dTMP, catalysing the methylation of dUMP to dTMP using a serine-derived one-carbon donor, the 5,10-methyleneTHF[94]. *TYMS* has been targeted for cancer chemotherapeutics, as high expression of which has been detected in various types of cancers, including gastrointestinal adenocarcinomas and squamous cell uterine carcinomas[93].

Chr19p13.3. The lead variant is located within intron 5 of *NMRK2* (*Nicotinamide Riboside kinase 2*), with 6 SNPs in high LD (r^2<0.8) located around this gene. NMRK2 enzyme catalyses the phosphorylation of nicotinamide riboside (NR) and nicotinic acid riboside (NaR) to form nicotinamide mononucleotide (NMN) and nicotinic acid mononucleotide (NaMN), the vitamin precursors of NAD^+, which is required for the function of Sirtuins, a key player in lifespan extension and energy metabolism[95]. It has been demonstrated that increased NAD^+ biosynthesis elevated the Sirtuin 2 function, which improved the subtelomeric gene silencing effects and elongated replicative lifespan in eukaryotic cell models[95]. One further variant in high LD, located upstream of *DAPK3* (*death associated protein kinase 3*), is a regulator of apoptosis. There is no functional data supporting any gene candidates at this locus.

Chr19p12. The lead variant is intergenic, located between *ZNF257* and *ZNF208*, with closer proximity to the former. There is eQTL evidence for both *ZNF257* and *ZNF265*, yet stronger for the *ZNF257* (Table S7). *ZNF257* encodes a member of a zinc finger protein family, the Krüppel-like zinc finger subfamily, signified by a consensus sequence of TGEKPYX (X denotes any amino acids) between concatenated zinc finger motifs[96]. The proteins have the KRAB domain at their amino terminus, which determines the specificity of binding to DNA and other transcriptional co-regulators.

Chr19q13.2. The single associated variant, rs11665818, is located within an intergenic region, downstream of *INFL2* (*interferon lambda 2*, also termed *IL28a*) and within a cytokine gene cluster that consists of three closely related *INFL* genes. *INFL2* encodes a protein with antiviral activities, predominantly in the epithelial tissues[97]. There is no supporting functional evidence at this locus.

Chr20p12.3a. The lead and one variant in high LD (r^2<0.8) are located upstream of *PROKR2* (*Prokineticin receptor 2*), a G protein-coupled receptor for the prokineticin 2, which is a secreted protein expressed in gut and brain, and has been shown to oscillate on a circadian basis[98]. Homozygous gene-knockout mice showed impaired circadian behaviour and thermoregulation (MGI:2181363). Mutations in this gene led to gonadotropin-releasing hormone deficiency and hypogonadism[99]. There are no coding variants or eQTLs associated with this locus.

Chr20p12.3b All variants of this locus are located within an intergenic region, with the closest gene being LINC01706 (long intergenic non-coding RNA 1706), an uncharacterised non-coding transcript.

Chr20q11.23. The association signal spans two genes *MROH8* (*maestro heat like repeat family member 8*) and *RBL1* (*RB transcriptional corepressor like 1*). There is eQTL evidence to support changes in both *RBL1* and *SAMHD1* (*SAM and HD domain containing deoxynucleoside triphosphate triphosphohydrolase 1*) expression. RBL1 functions as a transcriptional repressor for E2F binding sites-containing genes[100], which shares similarity in amino acid sequence and biochemical features to the *retinoblastoma 1* (*RB1*) gene product that functions as a tumour suppressor implicated in cell cycle regulation. *SAMHD1* encodes a dNTP triphosphohydrolase (dNTPase) that converts deoxynucleoside triphosphates (dNTPs) to deoxynucleosides. The gene expression was regulated during cell cycle to maintain a homeostatic pool of dNTP,

required for DNA replication [101]. Studies have suggested an antiretroviral role of SAMHD1 in dendritic and myeloid cells by depleting the intracellular pool of dNTPs[102,103].

Chr20q13.33. There are four independent signals within this locus, which harbours several genes, including the DNA helicase *RTEL1* (*regulator of telomere elongation helicase 1*). There are non-synonymous coding variants in *RTEL1* and *ZBTB46* (*zinc finger and BTB domain containing 46*) although neither are predicted to be deleterious. There are eQTLs for *RTEL1*, *STMN3*(*stathmin 3*) and *TNFRSF6B* (*TNF receptor superfamily member 6b*, also termed *decoy receptor 3*). *RTEL1* encodes an ATP-dependent DNA helicase that functions in the regulation of telomeres, DNA repair and genomic integrity. RTEL1 facilitates access of telomerase to the 3' ends of telomeres by transiently dismantling the T-loop configuration, a lariat-like structure that protects telomeres from degradation and deleterious DNA damage response[104]. Mutations of this gene led to Hoyeraal Hreidarsson syndrome, a clinically severe form of dyskeratosis congenita, of which half of the inherited families carry germline mutations of telomere-related genes[105]. Loss-of-function missense variants of this gene was found to be associated with idiopathic pulmonary fibrosis and shortened telomere lengths[106]. *STMN3* gene encodes a member of the stathmin protein family, which shows microtubule-destabilizing activity and is known to be involved in the development of central nervous system and glioma pathology[107]. *TNFRSF6B* is a regulator of apoptosis and has been linked to angiogenesis[108–110]. *ZBTB46* gene encodes a member of a large BTB zinc-finger protein family, characterised by a DNA binding motif that consists of a tandem array of C2H2 krüppel-like zinc fingers at the carboxyl terminus, with each finger containing a consensus sequence of ~30 amino acids and an embedded zinc ion[111]. In contrast, the BTB domains at the amino termini are more divergent across the family, mainly contributing to the hetero- or homo-dimerization. The BTB domain determines DNA binding specificity and recruitment of co-regulators to form higher chromosomal structures[111]. ZBTB46 has been shown to function as a transcriptional repressor involved in prostate cancer malignancy and cell cycle regulation[112]. Recently, studies have identified another member of the BTB zinc-finger protein family, ZBTB48, also termed as the telomeric zinc finger–associated protein, to be specifically associated with telomeres via the zinc finger domain. Further investigation demonstrated that it was preferentially bound to longer telomeres where protein components of the shelterin complex are rather sparse[113]. Experimental studies suggested that the ZBTB48 might compete with the TERF2 for binding to the telomeric DNA repeats, thereby setting an upper limit of the telomere length, which can further influence lifespan and cancer susceptibility[113,114]. Because the zinc finger domain is conserved among all members of the family, we speculated that the ZBTB46 was also capable of binding to the telomeric DNA, regulating telomere homeostasis via similar mechanisms. However, further experiments are required to validate this hypothesis.

Chr21q22.3. The lead variant is a loss-of-stop mutation in *KRTAP10-4* (*keratin associated protein 10-4*), which was located within a cluster of related genes, encoding proteins that form disulfide bonds between cysteine residues in hair keratins. A genome-wide siRNA-based screen implicated this gene with the homologous recombination DNA double-strand break repair[115]. Although transcripts lacking stop codons would be targeted for degradation, there is no eQTL evidence to suggest loss of expression with this allele, possibly due to poor detection of this transcript in GTex (Median transcripts per million=0). There is one variant in

high LD, located within intron 2 of *TSPEAR* (*thrombospondin type laminin G domain and EAR repeats*), a regulator of the NOTCH signalling.

Chr22q13.31. This is a single variant located within intron 1 of *KIA1644* (Also termed *SHISAL1*). There is no supporting functional data for gene prioritisation at this locus.

Supplemental Acknowledgements

ENGAGE cohorts

BHF-FHS. The British Heart Foundation Family Heart Study (BHF-FHS) was funded by the British Heart Foundation (BHF). Genotyping of the BHF-FHS study was undertaken as part of the WTCCC and funded by the Wellcome Trust.

EGCUT. EGCUT received financing from Horizon2020 programs (ePerMed), targeted financing from Estonian Government IUT 20-60 Estonian Research Roadmap through Estonian Ministry of Education and Research, Centre of Excellence in Genomics and Translational Medicine (GenTransMed). The work of Krista Fischer and Reedik Mägi has also been supported by Estonian Science Foundation grant EstSF ETF9353. We acknowledge EGCUT technical personnel, especially Mr V. Soo and S. Smit. Data analyzes were carried out in part in the High Performance Computing Centre of University of Tartu.

ERF. The ERF study as a part of EUROSPAN (European Special Populations Research Network) was supported by European Commission FP6 STRP grant number 018947 (LSHG-CT-2006-01947) and also received funding from the European Community’s Seventh Framework Programme (FP7/2007-2013)/grant agreement HEALTH-F4-2007-201413 by the European Commission under the programme “Quality of Life and Management of the Living Resources” of 5th Framework Programme (no. QLG2-CT-2002-01254). High-throughput analysis of the ERF data was supported by joint grant from Netherlands Organization for Scientific Research and the Russian Foundation for Basic Research (NWO-RFBR 047.017.043). We are grateful to all study participants and their relatives, general practitioners and neurologists for their contributions and to P. Veraart for her help in genealogy, J. Vergeer for the supervision of the laboratory work and P. Snijders for his help in data collection.

Finnish Twin Cohort/Nicotine Addiction Genetics-Finland study. This work was supported for data collection by Academy of Finland grants (J Kaprio) and a NIH grant DA12854 (PAFM). Genome-wide genotyping in the Finnish sample was funded by Global Research Award for Nicotine Dependence / Pfizer Inc. (JK), and Wellcome Trust Sanger Institute, UK. This work was further supported by the Academy of Finland Centre of Excellence in Complex Disease Genetics (grant numbers: 213506, 129680, J Kaprio) and the European Community's Seventh Framework Programme ENGAGE Consortium, (grant agreement HEALTH-F4-2007-201413). We thank Sirli Raud and Anne Vikman for help in TL measurement.

Finrisk. The FINRISK 2007/ DILGOM-study was supported by the Academy of Finland, grant # 118065. S.M. was supported by grants #136895 and #141005 and V.S. by grants #139635 and 129494 from the Academy of Finland.

GRAPHIC. The GRAPHIC study was funded by the BHF.

HBCS. The study was supported by grants to J.G.E. from the Academy of Finland (JGE: 135072, 129255, 126775), Finska Läkaresällskapet, Samfundet Folkhälsan, Juho Vainio Foundation, Päivikki and Sakari Sohlberg Foundation, Signe and Ane Gyllenberg Foundation, and Yrjö Jahnsson Foundation.

KORA. The KORA Augsburg studies were financed by the Helmholtz Zentrum München, German Research Centre for Environmental Health, Neuherberg, Germany and supported by grants from the German Federal Ministry of Education and Research (BMBF). Part of this work was financed by the German National Genome Research Network (NGFN). Our research was supported within the Munich Centre of Health Sciences (MC Health) as part of LMUinnovativ.

LLS The Leiden Longevity Study has received funding from the European Union's Seventh Framework Programme (FP7/2007-2011) under grant agreement n° 259679. This study was supported by a grant from the Innovation-Oriented Research Program on Genomics (SenterNovem IGE05007), the Centre for Medical Systems Biology, and the Netherlands Consortium for Healthy Ageing (grant 050-060-810), all in the framework of the Netherlands Genomics Initiative, Netherlands Organization for Scientific Research (NWO).

NESDA: Funding was obtained from the Netherlands Organization for Scientific Research (Geestkracht program grant 10-000-1002); the Centre for Medical Systems Biology (CSMB, NWO Genomics), Biobanking and Biomolecular Resources Research Infrastructure (BBMRI-NL), VU University's Institutes for Health and Care Research (EMGO+) and Neuroscience Campus Amsterdam, University Medical Centre Groningen, Leiden University Medical Centre, National Institutes of Health (NIH, R01D0042157-01A, MH081802, Grand Opportunity grants 1RC2 MH089951 and 1RC2 MH089995). Part of the genotyping and analyses were funded by the Genetic Association Information Network (GAIN) of the Foundation for the National Institutes of Health. Computing was supported by BiG Grid, the Dutch e-Science Grid, which is financially supported by NWO.

NFBC Northern Finland Birth Cohort 1966 (NFBC1966): NFBC1966 received financial support from the Academy of Finland (project grants 104781, 120315, 129269, 1114194,

139900/24300796, Centre of Excellence in Complex Disease Genetics and SALVE), University Hospital Oulu, Biocentre, University of Oulu, Finland (75617), the European Commission (EURO-BLCS, Framework 5 award QLG1-CT-2000-01643), NHLBI grant 5R01HL087679-02 through the STAMPEED program (1RL1MH083268-01), NIH/NIMH (5R01MH63706:02), ENGAGE project and grant agreement HEALTH-F4-2007-201413, the Medical Research Council, UK (G0500539, G0600705, G0600331, PrevMetSyn/SALVE, PS0476) and the Wellcome Trust (project grant GR069224, WT089549), UK.Replication genotyping was supported in part by MRC grant G0601261, Wellcome Trust grants 085301, 090532 and 083270, and Diabetes UK grants RD08/0003704 and BDA 08/0003775.The DNA extractions, sample quality controls, biobank up-keeping and aliquotting was performed in the National Public Health Institute, Biomedicum Helsinki, Finland and supported financially by the Academy of Finland and Biocentrum Helsinki. We thank Professor (emerita) Paula Rantakallio (launch of NFBC1966 and 1986), and Ms Outi Tornwall and Ms Minttu Jussila (DNA biobanking). The authors would like to acknowledge the contribution of the late Academian of Science Leena Peltonen. JLB was supported by a Wellcome Trust fellowship grant (WT088431MA).

NTR. We thank the Netherlands Organization for Scientific Research (NWO: MagW/ZonMW): Genotype/phenotype database for behaviour genetic and genetic epidemiological studies (NWO 911-09-032); Spinozapremie (SPI 56-464-14192); NWO-Groot 480-15-001/674: Netherlands Twin Registry Repository; BBMRI –NL: Biobanking and Biomolecular Resources Research Infrastructure (184.021.007 and 184.033.111). Amsterdam Public Health (APH) (Elucidating factors influencing the genetic background estimation in family members). European Science Foundation (ESF): Genomewide analyses of European twin and population cohorts (EU/QLRT-2001-01254); European Community's Seventh Framework Program (FP7/2007-2013): ENGAGE (HEALTH-F4-2007-201413); the European Science Council ERC- 230374; Rutgers University Cell and DNA Repository cooperative agreement (NIMH U24 MH068457-06); Collaborative study of the genetics of DZ twinning (NIH R01D0042157-01A); the Genetic Association Information Network, the Avera Institute for Human Genetics, Sioux Falls, South Dakota (USA), and the National Institutes of Mental Health (NIMH): GODOT: Integration of Genomics & Transcriptomics in Normal Twins & Major Depression (1RC2 MH089951-01) and DETECT: Developmental TrajECtories in Twins (1RC2MH089995-01). DIB acknowledges KNAW Academy Professor Award (PAH/6635).

QIMR. We thank Marlene Grace, Ann Eldridge, and Kerrie McAloney for sample collection; Anjali Henders, Megan Campbell, Lisa Bardsley, Lisa Bowdler, Steven Crooks, and staff of the Molecular Epidemiology Laboratory for sample processing and preparation; Harry Beeby, David Smyth, and Daniel Park for IT/database support; Scott Gordon for his substantial efforts involving the QC and preparation of the GWA data; and the twins and their families for their participation. We acknowledge support from the Australian Research Council (A7960034, A79906588, A79801419, DP0212016, and DP0343921). Telomere length

assessment was co-funded by the European Community's Seventh Framework Programme (FP7/2007-2013), ENGAGE project, grant agreement HEALTH-F4-2007-201413 and National Health and Medical Research Council (NHMRC)-European Union Collaborative Research Grant 496739. GWM was supported by an NHMRC Fellowship (619667), DRN (FT0991022) and SEM (FT110100548) were supported by an ARC Future Fellowship. Genotyping was funded by the NHMRC (Medical Bioinformatics Genomics Proteomics Program, 389891). Genotype imputation was carried out on the Genetic Cluster Computer (http://www.geneticcluster.org), which is financially supported by the Netherlands Scientific Organization (NWO 480-05-003).

TWINGENE. The Ministry for Higher Education, the Swedish Research Council (M-2005-1112), GenomEUtwin (EU/QLRT-2001-01254; QLG2-CT-2002-01254), NIH DK U01-066134, The Swedish Foundation for Strategic Research (SSF) Heart and Lung foundation no. 20070481

TwinsUK. TwinsUK is funded by the Wellcome Trust, Medical Research Council, European Union, the National Institute for Health Research - funded BioResource, Clinical Research Facility and Biomedical Research Centre based at Guy's and St Thomas' NHS Foundation Trust in partnership with King's College London.

UKBS: Recruitment of the United Kingdom Blood Service donors was funded by the Wellcome Trust as part of the WTCCC.

EPIC

EPIC-InterAct: We thank all EPIC participants and staff and the InterAct Consortium members for their contributions to the study. We thank staff from the technical, field epidemiology and data teams of the Medical Research Council Epidemiology Unit in Cambridge, UK, for carrying out sample preparation, DNA provision and quality control, genotyping and data handling work.

EPIC-CVD. this study was supported by core funding from the UK Medical Research Council (MR/L003120/1), the British Heart Foundation (RG/13/13/30194; RG/18/13/33946), the European Commission Framework Programme 7 (HEALTH-F2-2012-279233), and the National Institute for Health Research [Cambridge Biomedical Research Centre at the Cambridge University Hospitals NHS Foundation Trust]. JD is funded by the National Institute for Health Research [Senior Investigator Award].

The funding bodies had no role in the design or conduct of the study; collection, management, analysis, or interpretation of the data; preparation, review, or approval of the manuscript; or the decision to submit the manuscript for publication.

Where authors are identified as personnel of the International Agency for Research on Cancer / World Health Organization, NHS England, National Institute for Health Research or the Department of Health and Social Care, the authors alone are responsible for the views expressed in this article and they do not necessarily represent the decisions, policy or views of the corresponding organisations.

Supplementary References

1. Codd, V. *et al.* Identification of seven loci affecting mean telomere length and their association with disease. *Nat. Genet.* **45**, 422 (2013).
2. Kristiansson, K. *et al.* Genome-wide screen for metabolic syndrome susceptibility loci reveals strong lipid gene contribution but no evidence for common genetic basis for clustering of metabolic syndrome traits. *Circ. Cardiovasc. Genet.* **5**, 242–249 (2012).
3. Penninx, B. W. J. H. *et al.* The Netherlands Study of Depression and Anxiety (NESDA): rationale, objectives and methods. *Int. J. Methods Psychiatr. Res.* **17**, 121–140 (2008).
4. Ikram, M. A. *et al.* The Rotterdam Study: 2018 update on objectives, design and main results. *Eur. J. Epidemiol.* **32**, 807–850 (2017).
5. Langenberg, C. *et al.* Design and cohort description of the InterAct Project: An examination of the interaction of genetic and lifestyle factors on the incidence of type 2 diabetes in the EPIC Study. *Diabetologia* **54**, 2272–2282 (2011).
6. Langenberg, C. *et al.* Gene-Lifestyle Interaction and Type 2 Diabetes: The EPIC InterAct Case-Cohort Study. *PLoS Med.* **11**, (2014).
7. Danesh, J. *et al.* EPIC-Heart: The cardiovascular component of a prospective study of nutritional, lifestyle and biological factors in 520,000 middle-aged participants from 10 European countries. *Eur. J. Epidemiol.* **22**, 129–141 (2007).
8. Crowe, F. L. *et al.* Fruit and vegetable intake and mortality from ischaemic heart disease: results from the European Prospective Investigation into Cancer and Nutrition (EPIC)-Heart study. *Eur. Heart J.* **32**, 1235–1243 (2011).
9. Cawthon, R. M. Telomere length measurement by a novel monochrome multiplex quantitative PCR method. *Nucleic Acids Res.* **37**, e21–e21 (2009).
10. Verhoeven, J. E. *et al.* Major depressive disorder and accelerated cellular aging: results from a large psychiatric cohort study. *Mol. Psychiatry* **19**, 895–901 (2014).
11. Zhao, S. & Fernald, R. D. Comprehensive Algorithm for Quantitative Real-Time Polymerase Chain Reaction. *J. Comput. Biol.* **12**, 1047–1064 (2005).
12. Ma, Q. *et al.* MAGI3 negatively regulates Wnt/beta-catenin signaling and suppresses malignant phenotypes of glioma cells. *Oncotarget* **6**, 35851–65 (2015).
13. Ma, Q. *et al.* MAGI3 Suppresses Glioma Cell Proliferation via Upregulation of PTEN Expression. *Biomed. Environ. Sci.* **28**, 502–9 (2015).
14. Dell'Angelica, E. C., Mullins, C. & Bonifacino, J. S. AP-4, a novel protein complex related to clathrin adaptors. *J. Biol. Chem.* **274**, 7278–7285 (1999).
15. Hirst, J., Bright, N. A., Rous, B. & Robinson, M. S. Characterization of a fourth adaptor-related protein complex. *Mol. Biol. Cell* **10**, 2787–2802 (1999).
16. Bauer, P. *et al.* Mutation in the AP4B1 gene cause hereditary spastic paraplegia type 47 (SPG47). *Neurogenetics* **13**, 73–76 (2012).
17. Barber, E. K., Dasgupta, J. D., Schlossman, S. F., Trevillyan, J. M. & Rudd, C. E. The CD4 and CD8 antigens are coupled to a protein-tyrosine kinase (p56lck) that phosphorylates the CD3 complex. *Proc. Natl. Acad. Sci. U. S. A.* **86**, 3277–3281 (1989).
18. Iwashima, M., Irving, B. A., van Oers, N. S., Chan, A. C. & Weiss, A. Sequential interactions of the TCR with two distinct cytoplasmic tyrosine kinases. *Science* **263**, 1136–1139 (1994).
19. Sturm, R. A., Cassady, J. L., Das, G., Romo, A. & Evans, G. A. Chromosomal structure and expression of the human OTF1 locus encoding the Oct-1 protein. *Genomics* **16**, 333–341 (1993).

20. Segil, N., Roberts, S. B. & Heintz, N. Mitotic phosphorylation of the Oct-1 homeodomain and regulation of Oct-1 DNA binding activity. *Science* **254**, 1814–1816 (1991).
21. Roberts, S. B., Segil, N. & Heintz, N. Differential phosphorylation of the transcription factor Oct1 during the cell cycle. *Science* **253**, 1022–1026 (1991).
22. Schild-Poulter, C., Shih, A., Yarymowich, N. C. & Hache, R. J. G. Down-regulation of histone H2B by DNA-dependent protein kinase in response to DNA damage through modulation of octamer transcription factor 1. *Cancer Res.* **63**, 7197–7205 (2003).
23. Wysocka, J. & Herr, W. The herpes simplex virus VP16-induced complex: the makings of a regulatory switch. *Trends Biochem. Sci.* **28**, 294–304 (2003).
24. Lupo, B. & Trusolino, L. Inhibition of poly(ADP-ribosyl)ation in cancer: Old and new paradigms revisited. *Biochim. Biophys. Acta - Rev. Cancer* **1846**, 201–215 (2014).
25. Arnoult, N. & Karlseder, J. Complex interactions between the DNA-damage response and mammalian telomeres. *Nat. Struct. Mol. Biol* **22**, 859–866 (2015).
26. Déjardin, J. & Kingston, R. E. Purification of Proteins Associated with Specific Genomic Loci. *Cell* **136**, 175–186 (2009).
27. Liang, Y. *et al.* Association of ACYP2 and TSPYL6 Genetic Polymorphisms with Risk of Ischemic Stroke in Han Chinese Population. *Mol. Neurobiol.* **54**, 5988–5995 (2017).
28. Liu, M. *et al.* Association between single nucleotide polymorphisms in the TSPYL6 gene and breast cancer susceptibility in the Han Chinese population. *Oncotarget* **7**, 54771–54781 (2016).
29. Boulay, J. L., Dennefeld, C. & Alberga, A. The Drosophila developmental gene snail encodes a protein with nucleic acid binding fingers. *Nature* **330**, 395–398 (1987).
30. Hay, R. T. SUMO: A History of Modification. *Mol. Cell* **18**, 1–12 (2005).
31. Jones, a. M. *et al.* TERC polymorphisms are associated both with susceptibility to colorectal cancer and with longer telomeres. *Gut* **61**, 248–254 (2012).
32. Lührig, S. *et al.* Lrrc34, a novel nucleolar protein, interacts with npm1 and ncl and has an impact on pluripotent stem cells. *Stem Cells Dev.* **23**, 2862–74 (2014).
33. Fingerlin, T. E. *et al.* Genome-wide association study identifies multiple susceptibility loci for pulmonary fibrosis. *Nat. Genet.* **45**, 613–620 (2013).
34. Chow, A., Hao, Y. & Yang, X. Molecular characterization of human homologs of yeast MOB1. *Int. J. cancer* **126**, 2079–2089 (2010).
35. Lai, Z.-C. *et al.* Control of cell proliferation and apoptosis by mob as tumor suppressor, mats. *Cell* **120**, 675–685 (2005).
36. Kerjan, G. *et al.* Mice lacking doublecortin and doublecortin-like kinase 2 display altered hippocampal neuronal maturation and spontaneous seizures. *Proc. Natl. Acad. Sci. U. S. A.* **106**, 6766–6771 (2009).
37. Kiss, T., Fayet-Lebaron, E. & Jády, B. E. Box H/ACA Small Ribonucleoproteins. *Mol. Cell* **37**, 597–606 (2010).
38. Kwak, J. E., Wang, L., Ballantyne, S., Kimble, J. & Wickens, M. Mammalian GLD-2 homologs are poly(A) polymerases. *Proc. Natl. Acad. Sci. U. S. A.* **101**, 4407–4412 (2004).
39. Glahder, J. A. & Norrild, B. Involvement of hGLD-2 in cytoplasmic polyadenylation of human p53 mRNA. *APMIS* **119**, 769–775 (2011).
40. Wyman, S. K. *et al.* Post-transcriptional generation of miRNA variants by multiple nucleotidyl transferases contributes to miRNA transcriptome complexity. *Genome Res.* **21**, 1450–1461 (2011).

41. Schmidt, C. K. *et al.* Systematic E2 screening reveals a UBE2D-RNF138-CtIP axis promoting DNA repair. *Nat. Cell Biol.* **17**, 1458–1470 (2015).
42. Lehner, B. *et al.* Analysis of a high-throughput yeast two-hybrid system and its use to predict the function of intracellular proteins encoded within the human MHC class III region. *Genomics* **83**, 153–167 (2004).
43. Tang, W., Kannan, R., Blanchette, M. & Baumann, P. Telomerase RNA biogenesis involves sequential binding by Sm and Lsm complexes. *Nature* **484**, 260–264 (2012).
44. Kim, M. K. *et al.* Regulation of telomeric repeat binding factor 1 binding to telomeres by casein kinase 2-mediated phosphorylation. *J. Biol. Chem.* **283**, 14144–14152 (2008).
45. Baumann, P. Pot1, the Putative Telomere End-Binding Protein in Fission Yeast and Humans. *Science (80-.).* **292**, 1171–1175 (2001).
46. Hockemeyer, D. & Collins, K. Control of telomerase action at human telomeres. *Nat. Struct. Mol. Biol.* **22**, 848–852 (2015).
47. Lange, T. De. Shelterin : the protein complex that shapes and safeguards human telomeres. *Genes Dev.* **19**, 2100–2110 (2005).
48. Speedy, H. E. *et al.* Germ line mutations in shelterin complex genes are associated with familial chronic lymphocytic leukemia. *Blood* **128**, 2319–2326 (2016).
49. Shimizu, A. *et al.* A novel giant gene CSMD3 encoding a protein with CUB and sushi multiple domains: a candidate gene for benign adult familial myoclonic epilepsy on human chromosome 8q23.3-q24.1. *Biochem. Biophys. Res. Commun.* **309**, 143–154 (2003).
50. Toomes, C. *et al.* The presence of multiple regions of homozygous deletion at the CSMD1 locus in oral squamous cell carcinoma question the role of CSMD1 in head and neck carcinogenesis. *Genes. Chromosomes Cancer* **37**, 132–140 (2003).
51. Scholnick, S. B. & Richter, T. M. The role of CSMD1 in head and neck carcinogenesis. *Genes, chromosomes & cancer* **38**, 281–283 (2003).
52. Otsuka, M., Mizuno, Y., Yoshida, M., Kagawa, Y. & Ohta, S. Nucleotide sequence of cDNA encoding human cytochrome c oxidase subunit VIc. *Nucleic Acids Res.* **16**, 10916 (1988).
53. Kile, B. T. *et al.* The SOCS box: a tale of destruction and degradation. *Trends Biochem. Sci.* **27**, 235–241 (2002).
54. Chen, L.-Y., Redon, S. & Lingner, J. The human CST complex is a terminator of telomerase activity. *Nature* **488**, 540–544 (2012).
55. Chang, C.-W., Hsu, W.-B., Tsai, J.-J., Tang, C.-J. C. & Tang, T. K. CEP295 interacts with microtubules and is required for centriole elongation. *J. Cell Sci.* **129**, 2501–2513 (2016).
56. Wu, X. *et al.* ATM phosphorylation of Nijmegen breakage syndrome protein is required in a DNA damage response. *Nature* **405**, 477 (2000).
57. Banin, S. *et al.* Enhanced Phosphorylation of p53 by ATM in Response to DNA Damage. *Science (80-.).* **281**, 1674 LP – 1677 (1998).
58. Fan, J. *et al.* Tetrameric Acetyl-CoA Acetyltransferase 1 Is Important for Tumor Growth. *Mol. Cell* **64**, 859–874 (2016).
59. Fukao, T. *et al.* Molecular cloning and sequence of the complementary DNA encoding human mitochondrial acetoacetyl-coenzyme A thiolase and study of the variant enzymes in cultured fibroblasts from patients with 3-ketothiolase deficiency. *J. Clin. Invest.* **86**, 2086–2092 (1990).

60. Liu, L. *et al.* MCAF1/AM is involved in Sp1-mediated maintenance of cancer-associated telomerase activity. *J. Biol. Chem.* **284**, 5165–5174 (2009).
61. Liu, L. *et al.* MCAF1/AM Is Involved in Sp1-mediated Maintenance of Cancer-associated Telomerase Activity. *J. Biol. Chem.* **284**, 5165–5174 (2009).
62. Jobert, L. *et al.* The Human Base Excision Repair Enzyme SMUG1 Directly Interacts with DKC1 and Contributes to RNA Quality Control. *Mol. Cell* **49**, 339–345 (2013).
63. Lee, J. & Zhou, P. DCAFs, the Missing Link of the CUL4-DDB1 Ubiquitin Ligase. *Mol. Cell* **26**, 775–780 (2007).
64. Gao, J. *et al.* The CUL4-DDB1 ubiquitin ligase complex controls adult and embryonic stem cell differentiation and homeostasis. *Elife* **4**, (2015).
65. Mangino, M. *et al.* DCAF4, a novel gene associated with leucocyte telomere length. *J. Med. Genet.* **52**, 157–162 (2015).
66. Axe, E. L. *et al.* Autophagosome formation from membrane compartments enriched in phosphatidylinositol 3-phosphate and dynamically connected to the endoplasmic reticulum. *J. Cell Biol.* **182**, 685 LP – 701 (2008).
67. Shen, Z., Huang, S., Fang, M. & Wang, X. ENTPD5, an Endoplasmic Reticulum UDPase, Alleviates ER Stress Induced by Protein Overloading in AKT-Activated Cancer Cells. *Cold Spring Harb. Symp. Quant. Biol.* **76**, 217–223 (2011).
68. Fang, M. *et al.* The ER UDPase ENTPD5 Promotes Protein N-Glycosylation, the Warburg Effect, and Proliferation in the PTEN Pathway. *Cell* **143**, 711–724 (2010).
69. Heeringa, S. F. *et al.* COQ6 mutations in human patients produce nephrotic syndrome with sensorineural deafness. *J. Clin. Invest.* **121**, 2013–2024 (2011).
70. Tsang, W. Y. *et al.* CP110 Cooperates with Two Calcium-binding Proteins to Regulate Cytokinesis and Genome Stability. *Mol. Biol. Cell* **17**, 3423–3434 (2006).
71. Hayashi, R., Goto, Y., Ikeda, R., Yokoyama, K. K. & Yoshida, K. CDCA4 is an E2F transcription factor family-induced nuclear factor that regulates E2F-dependent transcriptional activation and cell proliferation. *J. Biol. Chem.* **281**, 35633–35648 (2006).
72. Kranz, T. M. *et al.* The chromosome 15q14 locus for bipolar disorder and schizophrenia: is C15orf53 a major candidate gene? *J. Psychiatr. Res.* **46**, 1414–1420 (2012).
73. Ebinu, J. O. *et al.* RasGRP links T-cell receptor signaling to Ras. *Blood* **95**, 3199–3203 (2000).
74. Roose, J. P., Mollenauer, M., Gupta, V. A., Stone, J. & Weiss, A. A diacylglycerol-protein kinase C-RasGRP1 pathway directs Ras activation upon antigen receptor stimulation of T cells. *Mol. Cell. Biol.* **25**, 4426–4441 (2005).
75. van der Velden, L. M. *et al.* Heteromeric interactions required for abundance and subcellular localization of human CDC50 proteins and class 1 P4-ATPases. *J. Biol. Chem.* **285**, 40088–40096 (2010).
76. Paulusma, C. C. & Oude Elferink, R. P. J. The type 4 subfamily of P-type ATPases, putative aminophospholipid translocases with a role in human disease. *Biochim. Biophys. Acta* **1741**, 11–24 (2005).
77. Gao, L. *et al.* Identification of Rare Variants in ATP8B4 as a Risk Factor for Systemic Sclerosis by Whole-Exome Sequencing. *Arthritis Rheumatol.* **68**, 191–200 (2016).
78. Hosford, D. *et al.* Candidate Single-Nucleotide Polymorphisms From a Genomewide Association Study of Alzheimer Disease. *JAMA Neurol.* **65**, 45–53 (2008).
79. Palfreyman, M. T. & Jorgensen, E. M. Unc13 Aligns SNAREs and Superprimes Synaptic

Vesicles. *Neuron* **95**, 473–475 (2017).
80. McRory, J. E. *et al.* Molecular and functional characterization of a family of rat brain T-type calcium channels. *J. Biol. Chem.* **276**, 3999–4011 (2001).
81. Cribbs, L. L. *et al.* Cloning and characterization of alpha1H from human heart, a member of the T-type Ca2+ channel gene family. *Circ. Res.* **83**, 103–109 (1998).
82. Daniil, G. *et al.* CACNA1H Mutations Are Associated With Different Forms of Primary Aldosteronism. *EBioMedicine* **13**, 225–236 (2016).
83. Vitko, I. *et al.* Functional Characterization and Neuronal Modeling of the Effects of Childhood Absence Epilepsy Variants of CACNA1H, a T-Type Calcium Channel. *J. Neurosci.* **25**, 4844–4855 (2005).
84. Van Steensel, B., Smogorzewska, A. & De Lange, T. TRF2 protects human telomeres from end-to-end fusions. *Cell* **92**, 401–413 (1998).
85. Tian, Y. *et al.* C. elegans Screen Identifies Autophagy Genes Specific to Multicellular Organisms. *Cell* **141**, 1042–1055 (2010).
86. Smogorzewska, A. *et al.* Control of human telomere length by TRF1 and TRF2. *Mol. Cell. Biol.* **20**, 1659–68 (2000).
87. Inano, S. *et al.* RFWD3-Mediated Ubiquitination Promotes Timely Removal of Both RPA and RAD51 from DNA Damage Sites to Facilitate Homologous Recombination. *Mol. Cell* **66**, 622-634.e8 (2017).
88. Fu, X. *et al.* RFWD3-Mdm2 ubiquitin ligase complex positively regulates p53 stability in response to DNA damage. *Proc. Natl. Acad. Sci. U. S. A.* **107**, 4579–4584 (2010).
89. Schilders, G., Raijmakers, R., Raats, J. M. H. & Pruijn, G. J. M. MPP6 is an exosome-associated RNA-binding protein involved in 5.8S rRNA maturation. *Nucleic Acids Res.* **33**, 6795–6804 (2005).
90. Lehner, B. & Sanderson, C. M. A protein interaction framework for human mRNA degradation. *Genome Res.* **14**, 1315–1323 (2004).
91. Moon, D. H. *et al.* Poly(A)-specific ribonuclease (PARN) mediates 3'-end maturation of the telomerase RNA component. *Nat. Genet.* **47**, 1482–1488 (2015).
92. Mutahir, Z. *et al.* Thymidine kinase 1 regulatory fine-tuning through tetramer formation. *FEBS J.* **280**, 1531–1541 (2013).
93. Shintani, M., Urano, M., Takakuwa, Y., Kuroda, M. & Kamoshida, S. Immunohistochemical characterization of pyrimidine synthetic enzymes, thymidine kinase-1 and thymidylate synthase, in various types of cancer. *Oncol. Rep.* **23**, 1345–1350 (2010).
94. Anderson, D. D., Quintero, C. M. & Stover, P. J. Identification of a de novo thymidylate biosynthesis pathway in mammalian mitochondria. *Proc. Natl. Acad. Sci.* **108**, 15163 LP – 15168 (2011).
95. Tempel, W. *et al.* Nicotinamide riboside kinase structures reveal new pathways to NAD+. *PLoS Biol.* **5**, e263 (2007).
96. Han, Z. G. *et al.* Molecular cloning of six novel Krüppel-like zinc finger genes from hematopoietic cells and identification of a novel transregulatory domain KRNB. *J. Biol. Chem.* **274**, 35741–8 (1999).
97. Kotenko, S. V *et al.* IFN-lambdas mediate antiviral protection through a distinct class II cytokine receptor complex. *Nat. Immunol.* **4**, 69–77 (2003).
98. Prosser, H. M. *et al.* Prokineticin receptor 2 (Prokr2) is essential for the regulation of circadian behavior by the suprachiasmatic nuclei. *Proc. Natl. Acad. Sci.* **104**, 648 LP – 653 (2007).

99. Dodé, C. & Rondard, P. PROK2/PROKR2 Signaling and Kallmann Syndrome. *Front. Endocrinol. (Lausanne).* **4**, 19 (2013).
100. Zhu, L. *et al.* Inhibition of cell proliferation by p107, a relative of the retinoblastoma protein. *Genes Dev.* **7**, 1111–1125 (1993).
101. Franzolin, E. *et al.* The deoxynucleotide triphosphohydrolase SAMHD1 is a major regulator of DNA precursor pools in mammalian cells. *Proc. Natl. Acad. Sci. U. S. A.* **110**, 14272–14277 (2013).
102. Ryoo, J. *et al.* The ribonuclease activity of SAMHD1 is required for HIV-1 restriction. *Nat. Med.* **20**, 936–941 (2014).
103. Laguette, N. *et al.* SAMHD1 is the dendritic- and myeloid-cell-specific HIV-1 restriction factor counteracted by Vpx. *Nature* **474**, 654–657 (2011).
104. Margalef, P. *et al.* Stabilization of Reversed Replication Forks by Telomerase Drives Telomere Catastrophe. *Cell* **172**, 439-453.e14 (2018).
105. Ballew, B. J. *et al.* A recessive founder mutation in regulator of telomere elongation helicase 1, RTEL1, underlies severe immunodeficiency and features of Hoyeraal Hreidarsson syndrome. *PLoS Genet.* **9**, e1003695 (2013).
106. Stuart, B. D. *et al.* Exome sequencing links mutations in PARN and RTEL1 with familial pulmonary fibrosis and telomere shortening. *Nat. Genet.* **47**, 512 (2015).
107. Zhang, Y. *et al.* Overexpression of SCLIP promotes growth and motility in glioblastoma cells. *Cancer Biol. Ther.* **16**, 97–105 (2015).
108. You, R. *et al.* Apoptosis of dendritic cells induced by decoy receptor 3 (DcR3). **111**, 1480–1489 (2019).
109. Pitti, R. M. *et al.* Genomic amplification of a decoy receptor for Fas ligand in lung and colon cancer. *Nature* **396**, 699–703 (1998).
110. Yang, C.-R. *et al.* Soluble decoy receptor 3 induces angiogenesis by neutralization of TL1A, a cytokine belonging to tumor necrosis factor superfamily and exhibiting angiostatic action. *Cancer Res.* **64**, 1122–1129 (2004).
111. Chevrier, S. & Corcoran, L. M. BTB-ZF transcription factors, a growing family of regulators of early and late B-cell development. *Immunol. Cell Biol.* **92**, 481–8 (2014).
112. Chen, W.-Y. *et al.* Inhibition of the androgen receptor induces a novel tumor promoter, ZBTB46, for prostate cancer metastasis. *Oncogene* **36**, 6213 (2017).
113. Li, J. S. Z. *et al.* TZAP: A telomere-associated protein involved in telomere length control. *Science (80-.).* **355**, 638–641 (2017).
114. Jahn, A. *et al.* ZBTB48 is both a vertebrate telomere-binding protein and a transcriptional activator. *EMBO Rep.* **18**, 929–946 (2017).
115. Adamson, B., Smogorzewska, A., Sigoillot, F. D., King, R. W. & Elledge, S. J. A genome-wide homologous recombination screen identifies the RNA-binding protein RBMX as a component of the DNA-damage response. *Nat. Cell Biol.* **14**, 318–328 (2012).
