# Supplementary material for: Genome-wide Association Analysis in Humans Links Nucleotide Metabolism to Leukocyte Telomere Length
Source: Am J Hum Genet. 2020 Feb 27;106(3):389–404. doi: 10.1016/j.ajhg.2020.02.006 (PMC7058826; doi:10.1016/j.ajhg.2020.02.006)
Supplement: Document S3. Article plus Supplemental Information [file mmc3.pdf]

Genome-wide Association Analysis in Humans Links Nucleotide Metabolism to Leukocyte Telomere Length

Chen Li,[1,3,85] Svetlana Stoma,[2,3,85] Luca A. Lotta,[1,85] Sophie Warner,[2,85] Eva Albrecht,[4] Alessandra Allione,[5,6] Pascal P. Arp,[7] Linda Broer,[7] Jessica L. Buxton,[8,9] Alexessander Da Silva Couto Alves,[10,11] Joris Deelen,[12,13] Iryna O. Fedko,[14] Scott D. Gordon,[15] Tao Jiang,[16] Robert Karlsson,[17] Nicola Kerrison,[1] Taylor K. Loe,[18] Massimo Mangino,[19,20] Yuri Milaneschi,[21] Benjamin Miraglio,[22] Natalia Pervjakova,[23] Alessia Russo,[5,6] Ida Surakka,[22,24] Ashley van der Spek,[25] Josine E. Verhoeven,[21] Najaf Amin,[25] Marian Beekman,[13] Alexandra I. Blakemore,[26,27] Federico Canzian,[28] Stephen E. Hamby,[2,3] Jouke-Jan Hottenga,[14] Peter D. Jones,[2] Pekka Jousilahti,[29] Reedik Mägi,[23] Sarah E. Medland,[15] Grant W. Montgomery,[30] Dale R. Nyholt,[15,31] Markus Perola,[29,32] Kirsi H. Pietiläinen,[33,34] Veikko Salomaa,[29] Elina Sillanpää,[22,35] H. Eka Suchiman,[13] Diana van Heemst,[36] Gonneke Willemsen,[14] Antonio Agudo,[37] Heiner Boeing,[38] Dorret I. Boomsma,[14] Maria-Dolores Chirlaque,[39,40] Guy Fagherazzi,[41,42] Pietro Ferrari,[43] Paul Franks,[44,45] Christian Gieger,[4,46,47] Johan Gunnar Eriksson,[48,49,50] Marc Gunter,[43] Sara Hägg,[17] Iiris Hovatta,[51,52] Liher Imaz,[53,54] Jaakko Kaprio,[22,55] Rudolf Kaaks,[56] Timothy Key,[57]

(Author list continued on next page)

Leukocyte telomere length (LTL) is a heritable biomarker of genomic aging. In this study, we perform a genome-wide meta-analysis of LTL by pooling densely genotyped and imputed association results across large-scale European-descent studies including up to 78,592 individuals. We identify 49 genomic regions at a false dicovery rate (FDR) < 0.05 threshold and prioritize genes at 31, with five highlighting nucleotide metabolism as an important regulator of LTL. We report six genome-wide significant loci in or near *SENP7*, *MOB1B*, *CARMIL1*, *PRRC2A*, *TERF2,* and *RFWD3*, and our results support recently identified *PARP1, POT1*, *ATM,* and *MPHOSPH6* loci. Phenome-wide analyses in >350,000 UK Biobank participants suggest that genetically shorter telomere length increases the risk of hypothyroidism and decreases the risk of thyroid cancer, lymphoma, and a range of proliferative conditions. Our results replicate previously reported associations with increased risk of coronary artery disease and lower risk for multiple cancer types. Our findings substantially expand current knowledge on genes that regulate LTL and their impact on human health and disease.

Introduction

Telomeres are DNA-protein complexes found at the ends of eukaryotic chromosomes, and they serve to maintain genomic stability and determine cellular lifespan.[1] Telomere length (TL) declines with cellular divisions; this is due to the inability of DNA polymerase to fully replicate the 3′ end of the DNA strand (the "end replication problem"), and once

[1]MRC Epidemiology Unit, University of Cambridge, CB2 0SL, United Kingdom; [2]Department of Cardiovascular Sciences, University of Leicester, LE3 9QP, United Kingdom; [3]NIHR Leicester Biomedical Research Centre, Glenfield Hospital, Leicester, LE3 9QP, United Kingdom; [4]Institute of Epidemiology, Helmholtz Zentrum München—German Research Centre for Environmental Health, D-85764 Neuherberg, Germany; [5]Department of Medical Science, Genomic Variation and Translational Research Unit, University of Turin, 10126 Turin, Italy; [6]Italian Institute for Genomic Medicine (IIGM), 10126 Turin, Italy; [7]Department of Internal Medicine, Erasmus Medical Centre, Postbus 2040, 3000 CA, Rotterdam, the Netherlands; [8]School of Life Sciences, Pharmacy, and Chemistry, Kingston University, Kingston upon Thames, KT1 2EE, United Kingdom; [9]Genetics and Genomic Medicine Programme, UCL Great Ormond Street Institute of Child Health, London, WC1N 1EH, United Kingdom; [10]School of Public Health, Imperial College London, St Mary's Hospital, London W2 1PG, United Kingdom; [11]School of Biosciences and Medicine, University of Surrey, Guildford, GU2 7XH, United Kingdom; [12]Max Planck Institute for Biology of Ageing, D-50931, Cologne, Germany; [13]Department of Biomedical Data Sciences, Section of Molecular Epidemiology, Leiden University Medical Centre, PO Box 9600, 2300 RC, Leiden, the Netherlands; [14]Department of Biological Psychology, Vrije Universteit, 1081 BT Amsterdam, the Netherlands; [15]Genetic Epidemiology, QIMR Berghofer Medical Research Institute, Queensland, 4006 Australia; [16]BHF Cardiovascular Epidemiology Unit, Department of Public Health and Primary Care, University of Cambridge, CB1 8RN, United Kingdom; [17]Department of Medical Epidemiology and Biostatistics, Karolinska Institutet, Stockholm 17177, Sweden; [18]Department of Molecular Medicine, The Scripps Research Institute, La Jolla, CA 92037, USA; [19]Department of Twin Research and Genetic Epidemiology, Kings College London, London SE1 7EH, United Kingdom; [20]NIHR Biomedical Research Centre at Guy's and St Thomas' Foundation Trust, London SE1 9RT, United Kingdom; [21]Department of Psychiatry, Amsterdam Public Health and Amsterdam Neuroscience, Amsterdam UMC/Vrije Universiteit, 1081HJ, Amsterdam, the Netherlands; [22]Institute for Molecular Medicine Finland (FIMM), PO Box 20, 00014 University of Helsinki, Finland; [23]Estonian Genome Centre, Institute of Genomics, University of Tartu, 51010, Tartu, Estonia; [24]Division of Cardiovascular Medicine, Department of Internal Medicine, University of Michigan, Ann Arbor, MI 48109, USA; [25]Department of Epidemiology, Erasmus Medical Centre, Postbus 2040, 3000 CA, Rotterdam, the Netherlands; [26]Department of Life Sciences, Brunel University London, Uxbridge UB8 3PH, United Kingdom; [27]Department of Medicine, Imperial College London, London, W12 0HS, United Kingdom; [28]Genomic Epidemiology Group, German Cancer Research Centre (DKFZ), 69120 Heidelberg, Germany; [29]Department of Public Health Solutions, Finnish Institute for Health and Welfare, PO Box 30, FI-00271 Helsinki, Finland; [30]Institute for Molecular Bioscience, The University of Queensland, 4072, Queensland, Australia; [31]School of Biomedical Sciences and Institute of Health and Biomedical Innovation, Queensland University of Technology, Queensland, 4059, Australia; [32]Research Program for Clinical and Molecular Metabolism, Faculty of Medicine, Biomedicum 1, PO Box 63, 00014 University of Helsinki, Finland; [33]Obesity Research

(Affiliations continued on next page)

© 2020 The Author(s). This is an open access article under the CC BY license (http://creativecommons.org/licenses/by/4.0/).

Vittorio Krogh,[58] Nicholas G. Martin,[15] Olle Melander,[59] Andres Metspalu,[23] Concha Moreno,[60] N. Charlotte Onland-Moret,[61] Peter Nilsson,[44] Ken K. Ong,[1,62] Kim Overvad,[63,64] Domenico Palli,[65] Salvatore Panico,[66] Nancy L. Pedersen,[17] Brenda W.J. H. Penninx,[21] J. Ramón Quirós,[67] Marjo Riitta Jarvelin,[10,68] Miguel Rodríguez-Barranco,[41,69,70] Robert A. Scott,[1] Gianluca Severi,[71,72,73] P. Eline Slagboom,[12,13] Tim D. Spector,[19] Anne Tjonneland,[74] Antonia Trichopoulou,[75] Rosario Tumino,[76,77] André G. Uitterlinden,[7] Yvonne T. van der Schouw,[61] Cornelia M. van Duijn,[25,78] Elisabete Weiderpass,[43] Eros Lazzerini Denchi,[18,79] Giuseppe Matullo,[5,6] Adam S. Butterworth,[16,80,81,83,84] John Danesh,[16,80,81,82,83,84] Nilesh J. Samani,[2,3] Nicholas J. Wareham,[1,85] Christopher P. Nelson,[2,3,85] Claudia Langenberg,[1,85,*] and Veryan Codd[2,3,85,*]

a critically short TL is reached, the cell enters replicative senescence.[2] Protein complexes, including the SHELTERIN complexes—which are comprised of TERF1 (MIM: 600951), TERF2 (MIM: 602027), POT1 (MIM: 606478), TERF2IP (MIM: 605061), TINF2 (MIM: 604319), ACD (MIM: 609377), and CST (CTC1 [MIM: 613129], STN1 [MIM: 613128], and TEN1 [MIM: 613130])—along with DNA helicases such as RTEL1 (MIM: 608833), bind telomeres and regulate TL and structure.[3] In some cell types, such as stem and germline progenitor cells, TL is maintained by the enzyme telomerase, a ribonucleoprotein containing the RNA template TERC (MIM: 602322), a reverse transcriptase (TERT [MIM: 187270]), and accessory proteins (DKC1 [MIM: 300126], NOP10 [MIM: 606471], GAR1 [MIM: 606468], and NHP2 [MIM: 606470]).[4]

Severe telomere loss, through loss-of-function mutations of core telomere and telomerase components, leads to several diseases which share features such as bone marrow failure and organ damage. These "telomere syndromes" include dyskeratosis congenita (MIM: 305000), aplastic anemia (MIM: 609135), and idiopathic pulmonary fibrosis (MIM:614742) among others.[5,6] While the prevalence of such syndromes varies, they are all relatively rare. One feature of these syndromes is premature aging.[5] Along with shorter TL observed at older ages in cross sectional population studies, this has led to TL (most commonly measured in human leukocytes as leucocyte telomere length [LTL]) to be proposed as a marker of biological age. LTL has been shown to be associated with the risk of common age-related diseases, including coronary artery

Unit, Research Program for Clinical and Molecular Metabolism, Haartmaninkatu 8, 00014 University of Helsinki, Helsinki, Finland; [34]Obesity Center, Abdominal Center, Endocrinology, Helsinki University Hospital and University of Helsinki, Haartmaninkatu 4, 00029 HUS, Helsinki, Finland; [35]Gerontology Research Center, Faculty of Sport and Health Sciences, PO Box 35, 40014 University of Jyväskylä, Finland; [36]Department of Internal Medicine, Section of Gerontology and Geriatrics, Leiden University Medical Centre, PO Box 9600, 2300 RC, Leiden, the Netherlands; [37]Unit of Nutrition, Environment, and Cancer, Cancer Epidemiology Research Program, Catalan Institute of Oncology—ICO, Group of Research on Nutrition and Cancer, Bellvitge Biomedical Research Institute—IDIBELL, L'Hospitalet of Llobregat, 08908 Barcelona, Spain; [38]German Institute of Human Nutrition Potsdam—Rehbruecke, 14558 Nuthetal, Germany; [39]Department of Epidemiology, Murcia Regional Health Council, IMIB—Arrixaca, 30008, Murcia, Spain; [40]CIBER of Epidemiology and Public Health (CIBERESP), 28029 Madrid, Spain; [41]Center of Research in Epidemiology and Population Health, UMR 1018 Inserm, Institut Gustave Roussy, Paris-Sud Paris-Saclay University, 94805 Villejuif, France; [42]Digital Epidemiology Research Hub, Department of Population Health, Luxembourg Institute of Health, L-1445 Strassen, Luxembourg; [43]International Agency for Research on Cancer, 69372 Lyon, France; [44]Department of Clinical Sciences, Clinical Research Center, Skåne University Hospital, Lund University, 20502 Malmö, Sweden; [45]Department of Public Health and Clinical Medicine, Umeå University, 90187 Umeå, Sweden; [46]Research Unit of Molecular Epidemiology, Helmholtz Zentrum München, German Research Center for Environmental Health, D 85764 Neuherberg, Germany; [47]German Center for Diabetes Research (DZD e.V.), D-85764 Neuherberg, Germany; [48]Department of General Practice and Primary Health Care, University of Helsinki and Helsinki University Hospital, PO Box 20, 00014 University of Helsinki, Finland; [49]Folkhälsan Research Centre, PO Box 20, 00014 University of Helsinki, Finland; [50]Obstetrics and Gynaecology, Yong Loo Lin School of Medicine, National University of Singapore, Singapore 117597; [51]SleepWell Research Program, Haartmaninkatu 3, 00014 University of Helsinki, Finland; [52]Department of Psychology and Logopedics, Haartmaninkatu 3, 00014 University of Helsinki, Finland; [53]Ministry of Health of the Basque Government, Public Health Division of Gipuzkoa, 20013 Donostia-San Sebastian, Spain; [54]Biodonostia Health Research Institute, 20014 Donostia-San Sebastian, Spain; [55]Department of Public Health, PO Box 20, 00014 University of Helsinki, Finland; [56]Division of Cancer Epidemiology, German Cancer Research Center (DKFZ), 69120 Heidelberg, Germany; [57]Cancer Epidemiology Unit, Nuffield Department of Population Health, University of Oxford, OX3 7LF, United Kingdom; [58]Epidemiology and Prevention Unit, Fondazione IRCCS—Istituto Nazionale dei Tumori, 20133 Milan, Italy; [59]Department of Clinical Sciences, Hypertension, and Cardiovascular Disease, Lund University, 21428 Malmö, Sweden; [60]Instituto de Salud Pública, 31003 Pamplona, Spain; [61]Julius Center for Health Sciences and Primary Care, University Medical Center Utrecht, Utrecht University, 3584 CG Utrecht, the Netherlands; [62]Department of Paediatrics, University of Cambridge, CB2 0QQ, United Kingdom; [63]Department of Public Health, Aarhus University, DK-8000 Aarhus, Denmark; [64]Department of Cardiology, Aalborg University Hospital, DK-9000 Aalborg, Denmark; [65]Cancer Risk Factors and Life-Style Epidemiology Unit, Institute for Cancer Research—ISPRO, 50139 Florence, Italy; [66]Dipartimento di Medicina Clinica e Chirurgia, Federico II University, 80131 Naples, Italy; [67]Consejería de Sanidad, Public Health Directorate, 33006 Asturias, Spain; [68]School of Epidemiology and Biostatistics, Imperial College London, SW7 2AZ, United Kingdom; [69]Andalusian School of Public Health (EASP), 18080 Granada, Spain; [70]Instituto de Investigación Biosanitaria ibs.GRANADA, 18012 Granada, Spain; [71]CESP, Facultés de médecine, Université Paris, 94805 Villejuif, France; [72]Gustave Roussy, 94805 Villejuif, France; [73]Department of Statistics, Computer Science, Applications "G. Parenti," University of Florence, 50134 Firenze, Italy; [74]Danish Cancer Society Research Center, 2100 Copenhagen, Denmark; [75]Hellenic Health Foundation, 11527 Athens, Greece; [76]Cancer Registry and Histopathology Department, Provincial Health Authority (ASP), 97100 Ragusa, Italy; [77]Hyblean Association for Research on Epidemiology, No Profit Organization, 97100 Ragusa, Italy; [78]Nuffield Department of Population Health, University of Oxford, OX3 7LF, United Kingdom; [79]Laboratory of Chromosome Instability, National Cancer Institute, NIH, Bethesda, MD 20892 USA; [80]Health Data Research UK Cambridge, Wellcome Genome Campus and University of Cambridge, CB10 1SA, United Kingdom; [81]NIHR Blood and Transplant Research Unit in Donor Health and Genomics, Department of Public Health and Primary Care, University of Cambridge, CB1 8RN, United Kingdom; [82]Department of Human Genetics, Wellcome Sanger Institute, Hinxton, CB10 1SA, United Kingdom; [83]BHF Cambridge Centre of Excellence, School of Clinical Medicine, Addenbrookes' Hospital, Cambridge, CB2 0QQ, United Kingdom; [84]NIHR Cambridge Biomedical Research Centre, School of Clinical Medicine, Addenbrooke's Hospital, Cambridge CB2 0QQ, United Kingdom
[85]These authors contributed equally to this work
*Correspondence: Claudia.Langenberg@mrc-epid.cam.ac.uk (C.L.), vc15@leicester.ac.uk (V.C.)
https://doi.org/10.1016/j.ajhg.2020.02.006.

disease (CAD) and some cancers.[7–12] However, whether LTL (reflecting TL across tissues) was causally associated with disease or whether the observed associations may have been due to reverse causation or confounding was unclear.

LTL is both variable among individuals, from birth and throughout the life course, and highly heritable, with heritability estimates from 44%–86%.[13,14] Identification of genetic determinants of LTL through a genome-wide association study (GWAS) has allowed further studies to suggest a causal role for LTL in several diseases, including CAD, abdominal aortic aneurysm, several cancers, interstitial lung disease, and celiac disease.[15–19] However, these studies are limited due to the small number of genetic variants that have been identified that replicate between studies.[15,20–25] To further our understanding of LTL regulation and its relationship with disease, we have conducted a genome-wide association (GWA) meta-analysis of 78,592 individuals from the European Network for Genetic and Genomic Epidemiology (ENGAGE) study and from the European Prospective Investigation into Cancer and Nutrition (EPIC) Cardiovascular Disease (CVD) and InterAct studies.

Subjects and Methods

Full descriptions of the EPIC-CVD and EPIC-InterAct cohorts, along with the participating cohorts within the ENGAGE consortium, are given in the Supplemental Information.

LTL Measurements and QC Analysis

Mean LTL measurements were conducted using an established quantitative PCR technique which expressed TL as a ratio of the telomere repeat number (T) to a single-copy gene (S).[26,27] The majority of the ENGAGE samples were included within our previous analysis.[15] LTL measurements were standardized either by using a calibrator sample or by quantifying against a standard curve, depending on the laboratory (Table S1 and Supplemental Methods). Full details of the methodology employed by each laboratory, along with quality control (QC) parameters, is given in the Supplemental Information or is given in detail elsewhere.[15] Because the use of different calibrator samples or of standard curves for quantification can lead to different ranges in the *T/S* ratios being observed between laboratories, we standardized LTL by using a z-transformation approach ($z = (\mu - \mu_0)/\sigma$, μ, *T/S* ratio, μ_0, the mean *T/S* ratio, σ, standard deviation [SD]).

Genotyping, GWAS Analysis, and Study-Level QC

Genotyping platforms and imputation methods and panels varied across participating study centers. Detailed information about these is provided in Figure S1 and Table S2. A GWAS was run within each study through the use of linear regression under an additive mode of inheritance with adjustment for age, sex, and any study-specific covariates, including batch, center, and genetic principle components. There are 21 studies contributing to ENGAGE. For the EPIC InterAct and CVD studies, association analyses were stratified based on genotyping platform and disease status, resulting in nine strata. Within each study or stratum, related samples ($k > 0.088$) were removed. Population stratification was estimated using the genomic control inflation factor λ and used to adjust the standard errors. Genetic variants were filtered on the basis of the published standards that included call rate >95%, Hardy–Weinberg equilibrium $p < 1 \times 10^{-6}$, imputation quality info-score >0.4 or $R^2 > 0.3$, minor allele count $\geqq 10$, and standard error of association estimates ranging from 0 to 10.[15,28,29] These data were taken forward to the meta-analysis.

Meta-analyses

GWAS summary statistics were combined via two steps of meta-analyses by using inverse variance weighting in GWAMA.[30] We first combined all 21 ENGAGE studies together and separately combined the nine EPIC-InterAct and EPIC-CVD strata, where a genetic variant was retained if it had >40% of the available sample size within these two cohorts. Fixed effects were used except for variants with significant heterogeneity (Cochrane's Q: $p < 1 \times 10^{-6}$), in which case random effects were used. Additional adjustment was made for genomic inflation (see Figure S2). In the second step, association estimates derived from the two separate meta-analyses estimated in the first step were combined using fixed effects inverse variance weighted meta-analyses. We estimated the FDR by estimating q-values[31] for these data.

Conditional Association Analysis

Conditionally independent signals were identified via an approximate genome-wide stepwise method, using GCTA (Version 1.25.2),[32,33] that allows for conditional analyses to be run on summary statistics without individual-level data. Summary statistics from the final meta-analysis were used as the input, with p value cut-offs at 5×10^{-8} (genome-wide significance) or 1.03×10^{-5} (equivalent to an FDR < 0.05). The model starts with the most significant SNP, adds in SNPs iteratively in a forward stepwise manner, and calculates conditional p values for all SNPs within the model. If the target SNP shows evidence of collinearity (correlation coefficients $r^2 > 0.9$, with linkage disequilibrium (LD) estimated based on a random subcohort of 50,000 UK Biobank samples) with any of the SNPs selected into the model, the conditional p value of the target SNP was set to 1. The selection process was repeated until no more SNPs could be fitted into the model, i.e., there were no more SNPs that could reach the conditional p value thresholds (5×10^{-8} or 1.03×10^{-5}, corresponding to the p value cut-offs in the input). Joint effects of all selected SNPs that fitted in the model were calculated and reported as independent variants' effects. Regional plots of a 1Mb window flanking the locus sentinel variants ($p < 5 \times 10^{-8}$) were generated using LocusZoom[34] with LD structure estimated in the UK Biobank subcohort (see Figure S3).

Gene Prioritization

Variant Annotation

Sentinel variants (conditional $p < 1.03 \times 10^{-5}$) and their proxies ($r^2 < 0.8$) were annotated on the human reference genome sequence hg19 using Annovar (v2017July16).[35] Their functional consequences on the protein sequences encoded by the nearest genes were cross-validated using definitions from RefGene,[36] Ensembl gene annotation,[37] GENCODE,[38] and the University of California, Santa Cruz (UCSC) human genome database.[39] These variants were also evaluated for features including evolutionary conservation (whether they reside in or specifically encode an conserved element based on multiple alignments across 46 vertebrate species), chromatin states predicted using Hidden

Markov Models trained by CHIP-seq data from ENCODE (15 classified states across nine cell types), histone modification markers (active promoter: H3K4Me3, H3K9Ac; active enhancer: H3K4me1, H3K27Ac; active elongation: H3K36me3; and repressed promoters and broad regions: H3K27me3), and CTCF transcription factor binding sites across nine cell lines, conserved putative TFBS, and DNaseI hypersensitive areas curated from the ENCODE database.[38] Variants within the exonic regions were further annotated with allele frequencies in seven ethnical groups (retrieved from the Exome Aggregation Consortium database) and functional effects prediction performed using a number of different algorithms. For non-coding variants, we performed integrated analysis with SNP Nexus IW scoring.[40]

Transcriptomic Data Integration

(1) With summary statistics, we performed a gene-level analysis, using S-PrediXcan, that links LTL to predicted gene expressions across 44 tissues (GTex v6p). It uses multivariate sparse regression models that integrate *cis*-SNPs within 2Mb windows around gene transcript boundaries in order to predict the corresponding gene expression levels. A detailed description of the method can be found elsewhere.[41,42] In brief, individual SNP-LTL associations were weighted by SNP-gene (w_{lg}) and SNP-SNP ($\sigma l/\sigma g$) association matrix, estimated from the PredictDB training set ($zg = \sum_{l \in g} wlg\ (\sigma l/\sigma g)\ zl$, for a gene ($g$); the set of SNPs ($l$) were selected from an elastic net model with a mixing parameter of 0.5). Protein-coding genes with qualified prediction model performance (average Pearson's correlation coefficients r^2 between predicted and observed gene expressions >0.01, FDR < 0.05) were included in our analysis. We considered a predicted gene expression to be significantly associated with LTL at a Bonferroni corrected p value threshold ($p < 2.61 \times 10^{-7}$), conservatively assuming association of each gene in each tissue as an independent test.

(2) For a given region significantly associated with LTL (FDR < 0.05), we tested whether the potential causal variants are shared between LTL and gene expressions by using COLOC Bayesian approach.[43] Regions for testing were determined as 2Mb windows surrounding the sentinel variants. Regional summary statistics were extracted from this GWA meta-analysis for associations with LTL and GTex v7[44] for *cis*-eGenes (genes with significant expression quantitative trait loci [eQTLs], FDR < 0.05) located within or on the boundaries of LTL regions defined. We selected the default priors for this analysis. We set $p1 = p2 = 10^{-4}$, meaning that 1 in 10,000 variants is associated with either trait (LTL or gene expression), as has been suggested by others.[43] We set $p12 = 10^{-5}$, meaning that 1 in 10 (p12/(p12 + p1)) variants that are associated with one trait is also associated with the other. This was chosen because sensitivity analyses have shown broadly consistent results between this setting and more stringent ($p12 = 10^{-5}$) settings, while allowing greater power.[45] Evidence for colocalization was assessed by comparing the posterior probability (PP) for two hypotheses: that the associations for both traits were driven by the same causal variants (hypothesis 4) and that they were driven by distinct ones (hypothesis 3). Strong evidence of a co-localized eQTL was defined as PP3 + PP4 ≥ 0.99 and PP4/PP3 ≥ 5, and suggestive evidence was defined as PP3 + PP4 ≥ 0.90 and PP4/PP3 ≥ 3, consistent with previous studies.[46,47]

Epigenomic (DNA Methylation) Data Integration

For genes whose expressions are modulated by epigenetic modifications, such as the methylation of transcriptional regulators in *cis*, linking genetic variants associated with *cis*-methylation probes (*cis*-meQTLs, FDR < 0.05) to LTL can help gene prioritization. For this: (1) We conducted a systematic search of LTL-associated sentinel variants and their proxies ($r^2 > 0.8$) in multiple publicly available meQTL databases.[48–50] (2) We also performed an epigenome-wide association analysis that integrated multiple variants' associations in a regularized linear regression model which was algorithmically similar to the transcriptome-wide association analyses.[51] A reference panel for meQTLs was constructed based on individuals in the EPIC-Norfolk cohort, with detailed description published elsewhere.[52] Bonferroni correction was applied, accounting for the total number of CpG markers tested ($p = 1.00 \times 10^{-7}$).

Pathway Enrichment Analysis

Using two different approaches, we sought to identify pathways that are responsible for regulating TL.

PANTHER

A list of our prioritized genes at each locus (or the nearest gene where no prioritization was possible) was submitted for statistical overrepresentation testing (Fisher's exact test) in Protein Analysis through Evolutionary Relationships (PANTHER).[53] Pathways (Gene Ontology [GO] molecular function complete annotation dataset) were considered over-represented where FDR p < 0.05.

DEPICT

We also used a hypothesis-free, data-driven approach using Data-driven Expression Prioritized Integration for Complex Traits (DEPICT)[54] to highlight reconstituted gene sets and tissue and/or cell types where LTL-associated loci were enriched. Summary statistics of uncorrelated SNPs (LD $r^2 \leqq 0.5$) significantly associated with LTL at a genome-wide level ($p < 5 \times 10^{-8}$) were used as the input, and the HLA region (chr6:29691116–33054976) was excluded. DEPICT first defined each locus around the uncorrelated variants and selected the genes within the region. It then characterized gene functions based on pairwise co-regulation of gene expressions, and these gene functions were quantified as membership probabilities across the 14,461 reconstituted gene sets. Then for each gene set, it assessed the enrichment by testing whether the sum of membership scores of all genes within each LTL-associated locus was higher than that for a gene-density-matched random locus. Detailed description of gene set construction was published elsewhere.[54] In brief, DEPICT leveraged a broad range of pre-defined pathway-oriented databases to construct gene sets (14,461), including GO terms,[55] KEGG,[56] REACTOME pathways,[57] the experimentally derived protein-protein interaction (PPI) subnetwork,[58] and the gene-phenotype matrix curated by Mouse Genetics Initiative.[59] Correlations ($r \geqq 0.3$) between significant gene sets were visualized using CytoScape.[60]

Clinical Relevance of LTL

Mendelian Randomization

Using two-sample Mendelian randomization (MR)[61] we investigated the potential effect of LTL on 122 diseases manually curated in the UK Biobank (Table S3).[62] Diseases were selected where there were sufficient case numbers to detect an odds ratio >1.1 (Table S4). LTL was genetically proxied based on 52 independently associated variants (FDR < 0.05). Individual SNP effects on disease were tested using logistic regression in SNPTEST,[63] adjusting for sex, age, the first five genetic principal components, and genotyping array within the UK Biobank. MR estimates were calculated using an inverse variance weighted MR approach. Sensitivity analyses were performed using median-based MR,[64] MR-RAPS,[65]

MR-Eggers,[66] and MR-Steigers[67] to identify inconsistency in the MR estimates, account for weak instrument bias, highlight any evidence of directional pleiotropy, and estimate direction of the MR relationship, repectively.

LD Score Regression

Cross-trait linkage disequilibrium score regression (LDSC) analysis was used to measure genetic correlations between LTL and selected traits through the use of the LD Hub database (version 1.4.1).[68] From the 832 available traits in LD Hub, we *a priori* selected traits of interest in order to remove redundancy and/or duplication within the analysis. We removed poorly defined traits and diseases, those without prior evidence of a genetic basis, and medications. We also removed lipid sub-fractions because we thought these unlikely to be relevant. We excluded studies with a sample size <1,000. Where multiple datasets for the same trait existed, we first prioritized datasets from large specialist consortia (where relevant factors would have been accounted for within the GWAS analysis) over the UK Biobank analyses conducted by the Neale group (where the GWAS was acknowledged to be a "quick and dirty" analysis). We then prioritized larger sample size, more recent studies, and diagnosed conditions over self-reported ones. We also removed traits with low heritability estimates within LD Hub, leaving us with 320 traits (information, including PMIDs of the selected studies, is given in the Results section).

Genome-wide summary statistics were used as the input, and standardized quality control was implemented within the software, including minor allele frequency (MAF) (>1% for HapMap3 and >5% for 1000 Genomes EUR-imputed SNPs), effective sample size (>0.67 times the 90th percentile of sample size), removal of insertions or deletions or structural variants, allelic alignment to 1000 Genomes, and removal of SNPs within the major histocompatibility complex (MHC) region.

Variants-based Cross-database Query

Independent variants and their strong proxies ($r^2 \geq 0.8$) were queried against publicly available GWAS databases; for this, we used PhenoScanner[69] for computational efficiency. A list of GWAS results implemented in the software was previously published. Results were filtered to include associations with $p < 1 \times 10^{-6}$, in high LD (r^2>0.8) with the most significant SNPs within the region, and manually curated to retain only the most recent and largest study per trait.

Results

Discovery of Genetic Determinants of LTL

Mean LTL was measured within each cohort by using a quantitative polymerase chain reaction (qPCR)-based method, which expresses TL as a ratio of telomere repeat content (T) to single-copy gene (S) within each sample (see Subjects and Methods, Supplemental Information, and Table S1). T/S ratios were z-standardized to harmonize differences in the quantification and calibration protocols between cohorts. Associations of shorter LTL with increasing age and male gender were observed as expected (Table S1).

Variants were assessed for association with mean LTL within each cohort through the use of additive models adjusted for age, gender, and cohort-specific covariates and then combined using inverse-variance-weighted meta-analysis (Table S2).

In total, 20 sentinel variants at 17 genomic loci were independently associated with LTL at a level of genome-wide statistical significance ($p < 5 \times 10^{-8}$, Table 1, Figure S1), including six loci that had not previously been associated with LTL (*SENP7* [MIM: 612846], *MOB1B* [MIM:609282], *CARMIL1* [MIM: 609593], *PRRC2A* [MIM: 142580], *TERF2*, and *RFWD3* [MIM: 614151]). We also identified genome-wide significant variants in four recently reported loci from a Singaporean Chinese population (*POT1*, *PARP1* [MIM: 173870], *ATM* [MIM:607585], and *MPHOSPH6* [MIM:605500])[70] and confirmed association at seven previously reported loci in European ancestry studies (*TERC*, *NAF1* [MIM: 617868], *TERT*, *STN1(OBFC1)*, *DCAF4* [MIM: 616372], *ZNF208* [MIM: 603977], and *RTEL1*).[15,23] Two and three conditionally independent signals were detected within the *TERT* and *RTEL1* loci, respectively (Table 1). Within the known loci, three variants within the *DCAF4* ($r^2 = 0.05$) and *TERT* ($r^2 < 0.5$) loci were distinct from the previously reported sentinel variants, while five (*TERC*, *NAF1*, *STN1*, *ZNF208*, and *RTEL1*; $r^2 > 0.8$; Table S5) were in high LD with the previously reported ones from European studies. For the loci identified in a Chinese ancestry population, we observed the same sentinel variant for *PARP1* and high LD variants for *ATM* and *MPHOSPH6* ($r^2 > 0.8$) but a distinct sentinel for *POT1* ($r^2 < 0.5$, Table S5). While we observed a distinct sentinel for *POT1*, we cannot rule out the possibility that the association signal observed in this region could be shared. In that case, the sentinels identified in each population would be reflective of a third, as yet unidentified, variant that is the true causal variant in this region. For the *RTEL1* locus, there are significant differences in LD structure between ancestral populations. All of the *RTEL1* variants we report at genome-wide statistical significance are in low LD with those reported in Singaporean Chinese and in South Asians.[25,70] Our novel variants are of lower frequency (MAF < 0.1) and either are reported as being monoallelic (monomorphic) or fall below the MAF threshold for analysis in the Southern Han Chinese (CHS) population (MAF < 0.01). This suggests that genetic variation in this region may be, in part, population specific or that the MAF is so low that we currently are unable to detect any association.

It has been shown that many loci that fall just below the conventional threshold of genome-wide significance are genuinely associated with the trait of interest and do subsequently reach the conventional threshold when sample size is increased.[71] In an attempt to gain additional insight into the genetic determination of LTL in humans, we applied a less stringent FDR threshold to the data. An additional 32 variants met an FDR threshold of <0.05, totaling 52 variants that estimate ~2.93% of the variance in TL (Table S6).[71] Within this FDR list, 5% of variants (2–3) are estimated to be false positives, although we are not able to determine which they are. While we believe that this FDR is acceptable, we advise that individual loci

Table 1. Independent Variants Associated with LTL at Genome-Wide Significance ($5x10^{-8}$)

SNP	Gene	Chr	Position (hg19)	EA	EAF	Beta	SE	p Value
**Previously Reported Loci**								
rs3219104	*PARP1*	1	226562621	C	0.83	0.042	0.006	9.60×10^{-11}
rs10936600	*TERC*	3	169514585	T	0.24	−0.086	0.006	7.18×10^{-51}
rs4691895	*NAF1*	4	164048199	C	0.78	0.058	0.006	1.58×10^{-21}
rs7705526	*TERT*	5	1285974	A	0.33	0.082	0.006	5.34×10^{-45}
rs2853677*	*TERT*	5	1287194	A	0.59	−0.064	0.006	3.35×10^{-31}
rs59294613	*POT1*	7	124554267	A	0.29	−0.041	0.006	1.17×10^{-13}
rs9419958	*STN1 (OBFC1)*	10	105675946	C	0.86	−0.064	0.007	5.05×10^{-19}
rs228595	*ATM*	11	108105593	A	0.42	−0.029	0.005	1.43×10^{-8}
rs2302588	*DCAF4*	14	73404752	C	0.10	0.048	0.008	1.68×10^{-8}
rs7194734	*MPHOSPH6*	16	82199980	T	0.78	−0.037	0.006	6.94×10^{-10}
rs8105767	*ZNF208*	19	22215441	G	0.30	0.039	0.005	5.42×10^{-13}
rs75691080	*RTEL1/STMN3*	20	62269750	T	0.09	−0.067	0.009	5.99×10^{-14}
rs34978822*	*RTEL1*	20	62291599	G	0.02	−0.140	0.023	7.26×10^{-10}
rs73624724*	*RTEL1/ZBTB46*	20	62436398	C	0.13	0.051	0.007	6.33×10^{-12}
**Additional Loci**								
rs55749605	*SENP7*	3	101232093	A	0.58	−0.037	0.007	2.45×10^{-8}
rs13137667	*MOB1B*	4	71774347	C	0.96	0.077	0.014	2.43×10^{-8}
rs34991172	*CARMIL1*	6	25480328	G	0.07	−0.061	0.011	6.19×10^{-9}
rs2736176	*PRRC2A*	6	31587561	C	0.31	0.035	0.006	3.53×10^{-10}
rs3785074	*TERF2*	16	69406986	G	0.26	0.035	0.006	4.64×10^{-10}
rs62053580	*RFWD3*	16	74680074	G	0.17	−0.039	0.007	4.08×10^{-8}

Gene—the closest or candidate gene (known telomere-related function) within the region. EA—effect allele. EAF—effect allele frequency within the study. Beta—the per-allele effect on z-scored LTL. SE—standard error.
*Additional, independent signals detected using conditional analysis are included.

should be interpreted with some caution. These variants were located within separate loci from those reported above, with the exception of a fourth, independent signal in the *RTEL1* locus. Although we did not replicate the previously reported *ACYP2* (MIM: 102595) locus, this did remain within the variants identified at the FDR < 0.05 threshold. *TYMS* (MIM: 188350), identified as genome-wide significant in a trans-ethnic meta-analysis of Singaporean Chinese[67] and in the previously reported ENGAGE analysis,[15] is within our FDR < 0.05 identified loci. This was to be expected considering the substantial sample overlap of the ENGAGE data; however, our sentinel variant is distinct and not reported in the Dorajoo et al. study. Aligning our data with available summary statistics from the Dorajoo et al. study (Singaporean Chinese samples only), we see at least nominal support for the vast majority of our genome-wide significant loci, with the exception of *STN1(OBFC1)* and *SENP7* (Table S7). Although *SENP7* has not previously been reported, variants in high LD ($r^2 > 0.6$) with our *STN1* sentinel have been reported in other European populations.[21,22] There is also support for many variants in our extended FDR list. However, it should be noted that data are not available for around half of our FDR < 0.05 loci, with most of these being either monoallelic or too low frequency to have been included within the analysis in the CHS population, again suggesting that several may be specific to the European population.

Prioritization of Likely Candidate Genes

We applied *in silico* prediction tools, leveraging large-scale human genomic data integrated with multi-tissue gene expression, transcriptional regulation, and DNA methylation data, coupled with knowledge-driven manual curation, to prioritisze the genes that are most likely influenced by the genetic variants within each locus. All 52 sentinel variants identified at GWS and FDR < 0.05 (listed in Table S6) plus their high LD proxies ($r^2 > 0.8$) were taken forward into our *in silico* analyses. First, we annotated all variants for genomic location and location with respect to regulatory chromatin marks (Tables S8 and S9). This also identified variants that led to non-synonymous changes in nine loci. Of these, five loci contained variants with predicted

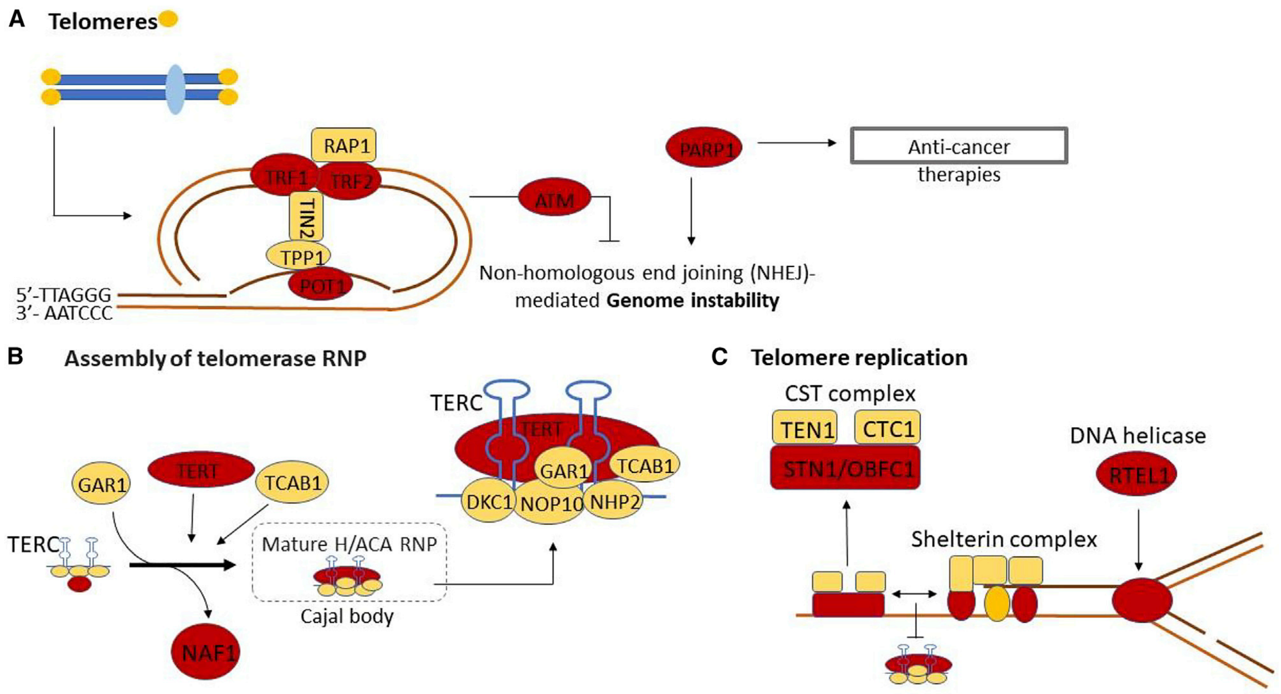

Figure 1. Loci with Established Roles in Telomere Biology
Candidate genes found in this study are shown in red. These include genes that encode components of the SHELTERIN complex (A), regulate the formation and activity of telomerase (B), and regulate telomere structure (C).

damaging effects on protein function (Table S10). We also found evidence that variants were associated with changes in gene expression in multiple loci (Table S11), with several showing co-localization and evidence from two approaches. This data, along with prediction of functional non-coding variants (Table S12), methylation QTL data (Table S13), and curation of gene functions within the region (Supplemental Methods), are summarized in Table S14. The summary data were utilized to prioritize genes that are most likely influenced at each locus. Where the prioritization methods suggested multiple genes for a given locus, we prioritized based on the amount of evidence across all considered lines of enquiry stated above. We were able to prioritize genes at 15 of the 17 genome-wide significant loci and 16 at of the 32 FDR loci (Table S14).

Four of the prioritized genes for newly identified loci have known roles in telomere regulation (*PARP1*, *POT1*, *ATM*, and *TERF2*; Figure 1). PARP1 (poly(ADP-ribose) polymerase 1), a variant in high LD ($r^2 = 1.0$) with our identified sentinel variant, causes a Val762Ala substitution (Table S10) which is known to reduce PARP1 activity.[72] This variant was associated with shorter LTL, in agreement with studies showing that knockdown of *PARP1* leads to telomere shortening.[73] PARP1 catalyzes the poly(ADP-ribosyl)ation of proteins in several cellular pathways, including DNA repair.[73] It interacts with TERF2 and it regulates the binding of TERF2 to telomeric DNA through this post-translational modification.[74]

Three genes, *DCAF4*, *SENP7*, and *RFWD3*, prioritized based on deleterious protein coding changes (*DCAF4*, *SENP7*) or strong evidence linking to gene expression levels (*RFWD3*), are all involved in DNA damage repair.[75–77] SENP7 has previously been demonstrated to bind damaged telomeres.[78] Components of DNA damage response and repair pathways (such as ATM) have been shown to also play roles in telomere regulation.[79] Mutations in *RFWD3* cause Fanconi anemia (MIM: 617784), a disease linked to telomere shortening and/or abnormalities.[80]

The *PRRC2A* locus contains 11 genetically linked SNPs located across the MHC class III region, which is a highly polymorphic and gene-dense region with complex LD structure. *BAG6* (MIM: 142590) and *CSNK2B* (MIM: 115441) were suggested as gene candidates for this region, supported by gene expression data (see Supplemental Information and Tables S11 and S14). BAG6 is linked to DNA damage signaling and apoptosis,[81] while CSNK2B, a subunit of casein kinase 2, interacts with TERF1 and regulates TERF1 binding at telomeres.[82]

Pathway Enrichment

To investigate context-specific functional connections between prioritized genes of the identified loci and to suggest plausible biological roles of these genes in the TL regulation, we performed enrichment analyses for pathways and tissues through the use of DEPICT[54] and PANTHER.[53] DEPICT is a hypothesis-free, data-driven approach for which we used summary statistics of all uncorrelated SNPs (LD $r^2 \leqq 0.5$) associated at $p < 5 \times 10^{-8}$ as input. For PANTHER, we assessed overrepresentation of genes within our loci within known pathways. To

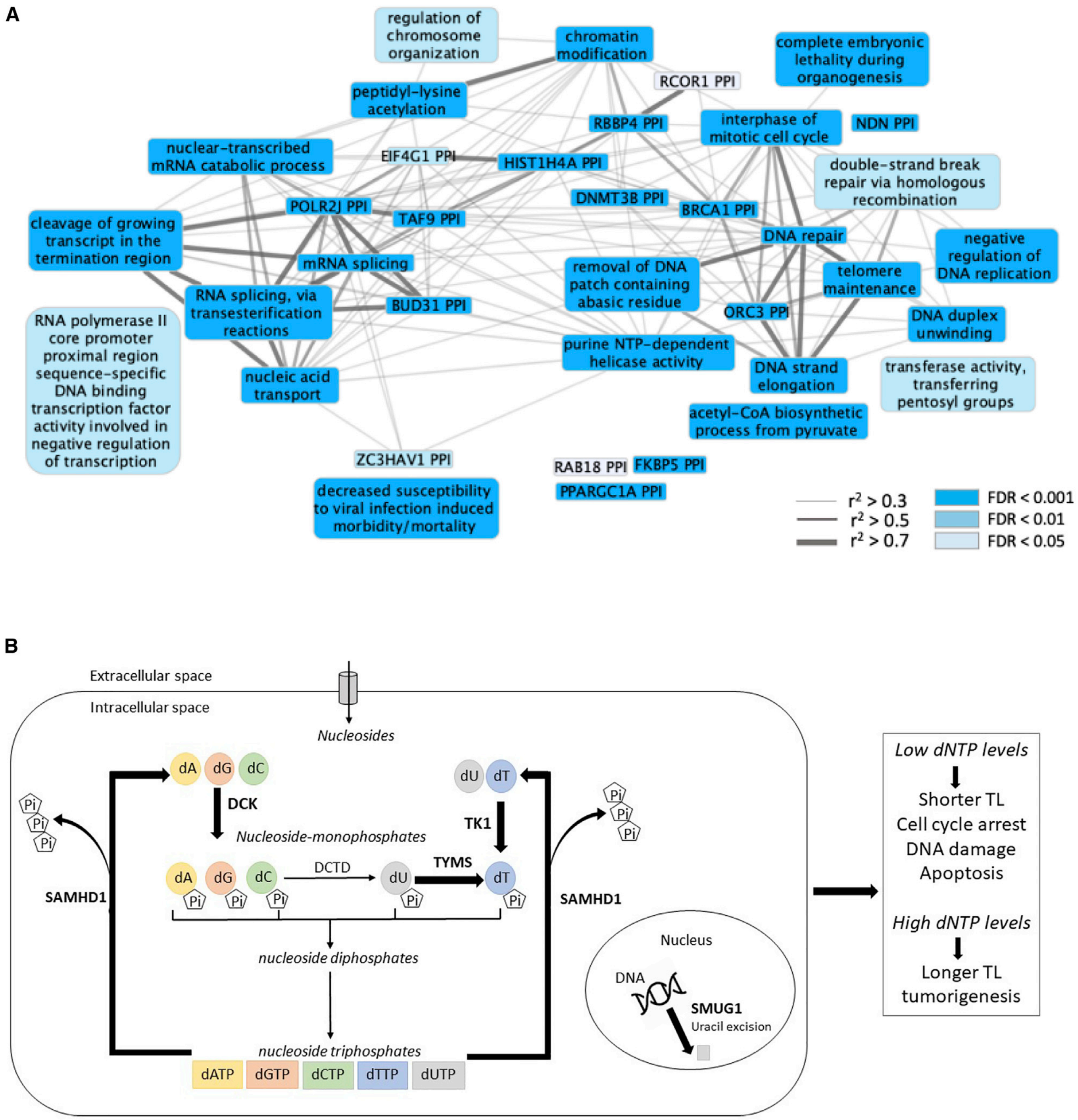

Figure 2. Pathways Enriched for Telomere-Associated Genes

(A) Gene sets significantly (false discovery rate [FDR] < 0.05) enriched for prioritised LTL-associated genes. Color intensity of the nodes (gene sets), classified into three levels, reflects enrichment strengths (FDR). Edge width indicates Pearson correlation coefficient (r^2) between each pair of the gene sets. Some of the most significantly associated gene sets include telomere maintenance along with DNA replication and repair pathways as may be expected. How other enriched pathways may influence LTL is unclear.

(B) Role of LTL-associated genes in nucleotide metabolism. Five enzymatic reactions and genes encoding the corresponding enzymes prioritized from this GWAS are highlighted in bold.

minimize noise, we used our prioritized genes as input, along with the closest gene to the sentinel SNP, where no prioritization was possible. In total, 55 genes were submitted to PANTHER, of which six were not available within PANTHER, leaving 49 within the analysis.

Over 300 reconstituted gene sets (DEPICT) were significantly enriched for the LTL loci (FDR < 0.05); these could be further clustered into 34 meta-gene sets, highlighting pathways that are involved in several major cellular activities, including DNA replication, transcription, and repair; cell cycle regulation; immune response; and intracellular trafficking (Figure 2A).

The PANTHER analysis identified a number of telomere-related pathways, including regulation of telomeric loop

disassembly, t-circle formation, protein binding at telomeres, and single-strand break repair, as being the mostly highly overrepresented (Table S15). Among other expected pathways, cellular aging and senescence were also highlighted. Of note, nucleotide metabolism pathways were overrepresented (2′-deoxyribonucleotide metabolic process, deoxyribose phosphate metabolic process, and deoxyribonucleotide metabolic process; Figure 2B; Table S15). The genes matched to these pathways were *TYMS*, *SAMHD1* (MIM: 606754), and *SMUG1* (MIM: 607753). While TYMS is critical for deoxythymidine monophosphate (dTMP) biosynthesis, SAMHD1 controls deoxynucleoside triphosphate (dNTP) catabolism and SMUG1 removes misincorporated uracil from DNA.[83–85] Although not highlighted in the pathway analysis, two further genes within other identified loci (*TK1* [MIM: 188300] and *DCK* [MIM:125450]) are key regulators of deoxynucleoside monophosphate (dNMP) biosynthesis;[85] this adds further support to the possibility that nucleotide metabolism is a key pathway in regulating LTL. dNTPs constitute the fundamental building blocks required for DNA replication and repair.[86] Genetic perturbations that disrupt dNTP homeostasis have been shown to result in increased replication error, cell cycle arrest, and DNA-damage-induced apoptosis.[85,87]

Relationship between Genetically Determined TL and Disease

To further understand the clinical relevance of TL, we used the 52 independent variants identified at FDR < 0.05 as genetic instruments for TL, and we applied a two-sample MR approach using UK Biobank data.[62] We manually curated 122 diseases available in the UK Biobank and examined their relationships with shorter TL (Tables S3 and S16). We observed nine associations which passed a Bonferroni corrected threshold ($p < 4.1\text{x}10^{-4}$). These included novel findings of an increased risk of hypothyroidism, and decreased risk of thyroid cancer, lymphoma, and diseases of excessive growth (uterine fibroids, uterine polyps, and benign prostatic hyperplasia). We also confirmed findings for decreased risk of lung and skin cancer and leukemia for subjects with shorter TL (Figure 3, Table S16).[16,18,88] We observed a further 30 nominally significant associations ($p < 0.05$), confirming previous MR findings of an increased risk of CAD, within the UK Biobank population (Figure 3, Table S16). Our results also provide genetic evidence for associations of shorter LTL with increased risk of rheumatoid arthritis, aortic valve stenosis, chronic obstructive pulmonary disease, and heart failure, all of which have previously been observationally associated with shorter LTL.[89–92] We also ran the MR analyses using only the genome-wide significant variants (Figure S4), and we did not lose any Bonferroni-significant hits, with only small differences in those diseases that are nominally associated. In our sensitivity analyses, effect estimates were consistent across MR methods. The MR-Steigers analysis indicated that the direction of the relationship is that TL influences disease risk. This analysis also indicated that this direction was estimated correctly for the majority of diseases (Table S16).

We next sought to explore human diseases and traits that share common genetic etiologies with LTL. We did this by performing LD score regression analyses to test for genetic correlations between TL and 320 curated traits and diseases (Table S17) within LD Hub.[15,16] In comparison to the MR approach, these analyses utilize genome-wide genetic information rather than selected SNPs with the most significant associations. In agreement with our MR analyses, TL was negatively correlated with CAD ($r = -0.17$, $p = 0.01$, Table S17). Dyslipidaemia risk factors for CAD also showed concordant associations with shorter TL, including higher LDL and total cholesterol and lower HDL cholesterol (Table S17). These results are suggestive of a shared genetic architecture underlying TL, CAD, and CAD risk factors. However, these results would not survive correction for multiple testing.

We also examined individual locus-driven genetic correlations between TL and a variety of human phenotypes and diseases by using PhenoScanner[69] to query 52 FDR sentinel variants and their closely related SNPs in LD ($r^2 \geqq 0.8$) against publicly available GWAS databases. While some morbidities showed specific correlations to a single locus, others showed correlations to a broader spectrum of loci. For example, self-reported hypothyroidism or myxoedema exhibited a strong association particularly at the *TERT* locus, which was also exclusively responsible for several subtypes of ovarian cancers (Table S18). In contrast, blood cell traits and hematological diseases were implicated with a wider range of loci, including *TERC*, *TERT*, *SENP7*, *ATM*, *BBOF1*, and *MROH8*; this result is similar to those for the respiratory function and lung cancers that also involved multiple TL loci (Table S18).

Discussion

We identify 20 lead variants at a level of genome-wide significance and a further 32 at FDR < 0.05. Within established loci, we report a second, independent, association signal within the *TERT* locus and redefine the *RTEL1* locus into three independent signals. By applying a range of *in silico* tools that integrate multiple lines of evidence, we were able to pinpoint likely influenced genes for the majority of independent lead variants (34 of 52), several of which represent key telomere-regulating pathways (including components of the telomerase complex, the telomere-binding SHELTERIN and CST complexes, and the DNA damage response [DDR] pathway).

Telomeres function to prevent the 3′ single-stranded overhang at the end of the chromosome from being detected as a double-stranded DNA break. This is achieved through binding of the SHELTERIN complex (TERF1, TERF2, TERF2IP, TINF2, ACD, and POT1), which acts to

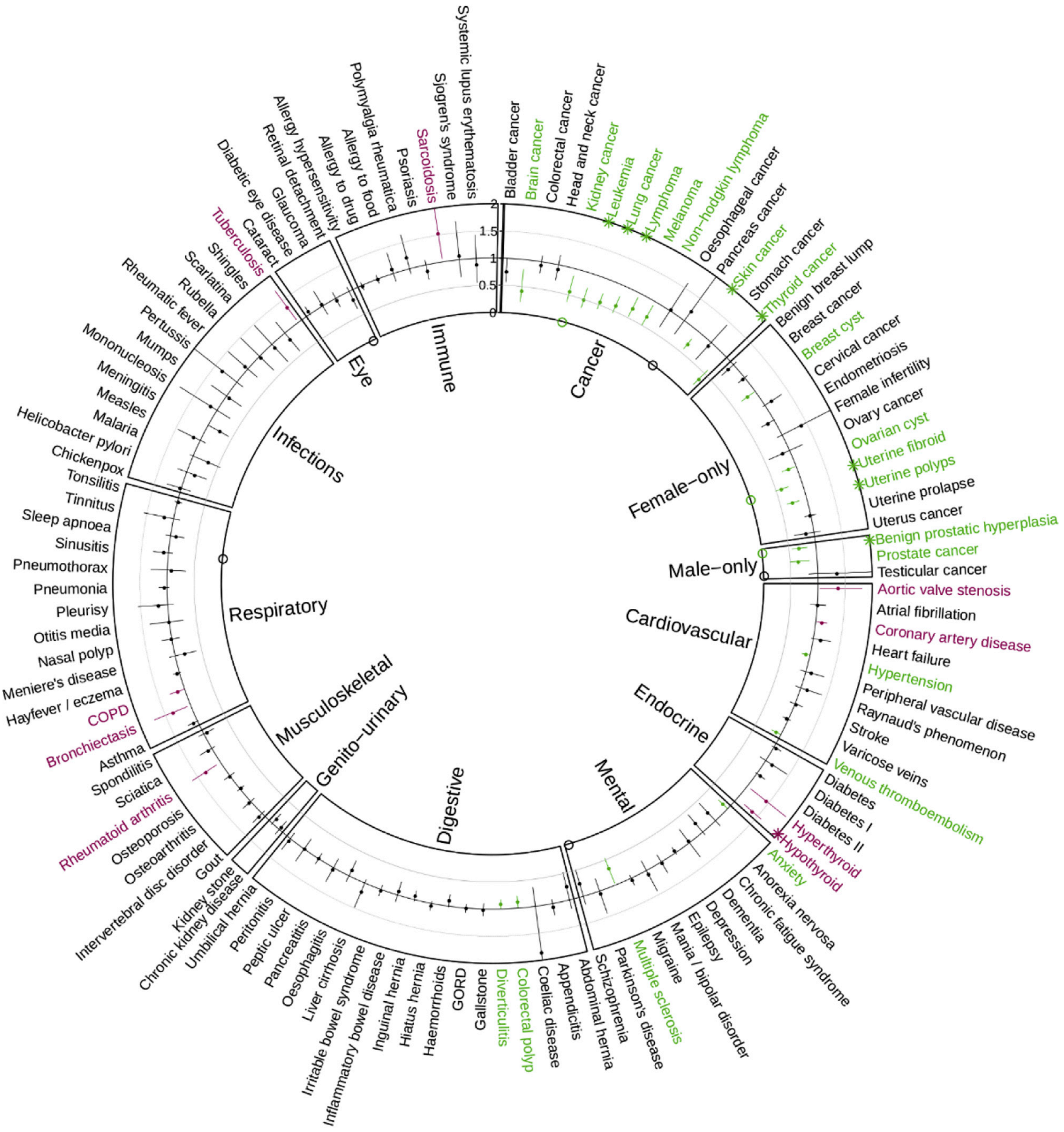

Figure 3. Mendelian Randomization Results for the Effect of Shorter LTL on the Risk of 122 Diseases in the UK Biobank
Data shown are odds ratios and 95% confidence intervals for a 1 standard deviation shorter LTL. Diseases are classified into groups, as indicated by the boxing, and sorted alphabetically within disease group. Nominally significant ($p < 0.05$) associations estimated via inverse-variance-weighted Mendelian randomization are shown in green for a reduction in risk and purple for an increase in risk due to shorter LTL. ^{O} indicates nominal ($p < 0.05$) evidence of pleiotropy estimated by MR-Eggers intercept. Full results are also shown in Table S16 along with the full MR sensitivity analysis.

block activation of DDR pathways via several mechanisms.[3] SHELTERIN also binds a number of accessory factors that facilitate processing and replication of the telomere, including the DNA helicase RTEL1.[3] SHELTERIN also interacts with the CST complex that regulates telomerase access to the telomeric DNA (Figure 1C).[3] The associated loci contain two of the SHELTERIN components (TERF2 and POT1), a regulator of TERF1, CSNK2B (*PRRC2A* locus),[82] the helicase RTEL1, and the CST component STN1.

Although telomere-binding proteins and structure aim to inhibit activation of DDR pathways, there is also evidence of a paradoxical involvement of a number of DDR factors in TL maintenance; these factors include both of the prioritized genes, *ATM* and *PARP1*.[73,93] TERF2 inhibits

ATM activation and the classical non-homologous end joining (c-NHEJ) at telomeres, thus preventing synapsis of chromosome ends (Figure 1A).[94] However, ATM activation is required for telomere elongation, potentially by regulating access of telomerase to the telomere end through ATM-mediated phosphorylation of TERF1.[93] It is possible that other DDR regulators can impact TL maintenance by regulating telomeric chromatin states, T-loop dynamics, and single-stranded telomere overhang processing.[79] Other prioritised genes (*SENP7* and *RFWD3*) also function within DDR pathways; this suggests a plausible mechanism through which they may influence LTL.

The telomerase enzyme is capable of extending telomeres and/or compensating sequence loss due to the end replication problem in stem and reproductive cells.[4] Associated loci include genes encoding the core telomerase components TERT and TERC along with the chaperone protein NAF1. NAF1 is required for *TERC* accumulation and its incorporation into the telomerase complex.[95] After transcription, *TERC* undergoes complex 3′ processing to produce the mature 451bp template.[96] This involves components of the RNA exosome complex, PARN (MIM: 604212) and TENT4B (MIM: 605540), among others; this process is not fully understood.[97] In addition to variants within regions containing *TERT*, *TERC*, and *NAF1*, a prioritiszed gene from another locus (*MPHOSPH6*) is a component of the RNA exosome.[98]

Comparing our findings to those reported in a non-European study,[70] we find support for our most significantly associated loci. For many of our FDR < 0.05 loci, we were unable to look for support from this study because our sentinel variants were either monoallelic or rare (MAF < 0.01) in the CHS population. Different LD structures in regions such as RTEL1, coupled with the reported absence of some of the variants in other ancestral populations, suggest that some of our reported variants may specific to Europeans. Adding additional support for the existence of population-specific rare variants regulating LTL is the discovery of two loci in the Singaporean Chinese study that are monoallelic in Europeans.[70] Because both of these replicate within CHS subjects and are located within regions containing telomere-related genes, they are unlikely to be false positive findings. Future large-scale trans-ethnic meta-analyses will be critical in determining shared causal variants from population-specific rare variants. This is of key importance to downstream analyses using genetically determined LTL to investigate disease risk in different populations. However, the current lack of large-scale data on LTL in non-European cohorts is limiting.

Utilizing the prioritized gene list as well as the closest genes to the sentinel variants, we showed a number of pathways to be enriched for telomere-associated loci. Of note, we observed significant overrepresentation of genes in several nucleotide metabolism pathways (Table S15, Figure 2B). Key genes were highlighted by this function in both the biosynthesis (*TYMS*, *TK1*, and *DCK*) and catabolism (*SAMHD1*) of dNTPs. Biosynthesis of dNTPs occurs via two routes: de-novo synthesis and the nucleotide salvage pathway. Thymidine kinase (TK1) and deoxycytidine kinase (DCK) are the rate-limiting enzymes that catalyze the first step of the salvage pathway of nucleotide biosynthesis, converting deoxynucleosides to their monophosphate forms (dNMPs) before other enzymes facilitate further phosphorylation into deoxynucleodie diphophates (dNDPs) and dNTPs (Figure 2B).[85] Thymidylate synthetase (TYMS) is considered to be a component of the *de novo* pathway, and is the key regulator of dTMP biosynthesis, converting deoxyuridine monophosphate (dUMP) to dTMP.[85] However, because the dUMP substrates can be derived from either *de novo* synthesis or deamination of deoxycytidine monophosphate (dCMP) produced from the salvage pathway, it could be considered to function within both pathways (Figure 2B).[85] Besides controlling biosynthetic pathways, the equilibrium of cellular dNTP levels is also achieved by regulating degradation of dNTPs, a key regulator of which is SAMHD1. It catalyzes the hydrolysis of dNTPs to deoxynucleosides and triphosphates, thereby preventing the accumulation of excess dNTPs (Figure 2B).[81] Although the finely tuned dNTP supply system inhibits incorrect insertions of bases into DNA synthesis, potential errors are monitored by the product of another prioritized gene, the base excision repair enzyme, SMUG1, which removes uracil and oxidized derivatives from DNA molecules.[84]

A balanced cellular pool of dNTPs is required for DNA replication and repair and for maintaining proliferative capacity and genome stability. Low levels of dNTPs can induce replication stress, subsequently leading to increased mutation rates.[99] A surplus of dNTPs, on the other hand, reduces replication fidelity, thus also causing higher levels of spontaneous mutagenesis.[100] A dynamic balance between biosynthesis and catabolism is required to maintain an equilibrium. Because maintaining the balance of the intracellular dNTP pool is also fundamental to other pathways that are implicated in telomere homeostasis, including cellular proliferation and DNA repair, disruption of dNTP homeostasis may trigger a sequence of cellular events that interplay synergistically, leading to abnormalities of TL and genome instability.

By clustering our prioritized genes via their functional connections, we highlighted a number of pathways that were enriched for TL regulation, which included DNA replication, transcription, and repair; cell cycle regulation; immune response; and intracellular trafficking. However, we noted that because the gene prioritization was based on integration of bioinformatic evidence from a number of publicly available databases, which also laid the foundation for establishing the pathways used in the enrichment analyses, this approach may suffer from self-fulfilling circular arguments.

While supporting previous evidence linking shorter TL to an increased risk of CAD and lower risk of several cancers, we demonstrated additional associations between TL and thyroid disease, thyroid cancer, lymphoma, and

several non-malignant neoplasms. Shorter TL was protective against all of these proliferative disorders, potentially through limiting cell proliferative capacity, which in turn reduces the occurrence of potential oncogenic mutations that can occur during DNA replication. Furthermore, we also provide evidence suggesting that shorter TL is potentially causally associated with increased risk of several cardiovascular, inflammatory, and respiratory disorders that have previously been linked to TL in observational studies. Our findings linking nucleotide metabolism to TL regulation could in part explain the link between TL and cancer and proliferative disorders. This would suggest that cells with longer TL have higher dNTP levels that lead to higher proliferation rates and reduced DNA replication fidelity leading to higher mutation rates.

In summary, our findings substantially expand current knowledge on the genetic determinants of LTL, and they elucidate genes and pathways that regulate telomere homeostasis and their potential impact on human diseases and cancer development.

Supplemental Data

Supplemental Data can be found online at https://doi.org/10.1016/j.ajhg.2020.02.006.

Acknowledgments

The ENGAGE Project was funded under the European Union Framework 7—Health Theme (HEALTH-F4-2007- 201413). The InterAct project received funding from the European Union (Integrated Project LSHM-CT-2006-037197 in the Framework Programme 6 of the European Community). The EPIC-CVD study was supported by core funding from the UK Medical Research Council (MR/L003120/1), the British Heart Foundation (RG/13/13/30194; RG/18/13/33946), the European Commission Framework Programme 7 (HEALTH-F2-2012-279233), and the National Institute for Health Research (Cambridge Biomedical Research Centre at the Cambridge University Hospitals National Health Service (NHS) Foundation Trust)[*]. C.P.N is funded by the British Heart Foundation (BHF). V.C., C.P.N., and N.J.S. are supported by the National Institute for Health Research (NIHR) Leicester Cardiovascular Biomedical Research Centre and N.J.S. holds an NIHR Senior Investigator award. Chen Li is support by a four-year Wellcome Trust PhD Studentship; C.L., L.A.L., and N.J.W. are funded by the Medical Research Council (MC_UU_12015/1). N.J.W. is an NIHR Senior Investigator. J.D. is funded by the NIHR (Senior Investigator Award).[*]. *The views expressed are those of the authors and not necessarily those of the NHS, the NIHR, or the Department of Health and Social Care. Cohort-specific and further acknowledgments are given in the Supplemental Information.

Declaration of Interests

A.S.B. holds grants unrelated to this work from AstraZeneca, Merck, Novartis, Biogen, and Bioverativ/Sanofi.

J.D. reports personal fees and non-financial support from Merck Sharpe and Dohme UK Atherosclerosis; personal fees and non-financial support from Novartis Cardiovascular and Metabolic Advisory Board; personal fees and non-financial support from Pfizer Population Research Advisory Panel; and grants from the British Heart Foundation, the European Research Council, Merck, the NIHR, NHS Blood and Transplant, Novartis, Pfizer, the UK Medical Research Council, Health Data Research UK, and the Wellcome Trust outside the submitted work.

Received: October 22, 2019
Accepted: February 10, 2020
Published: February 27, 2020

Web Resources

Ensembl Genome Browser, https://useast.ensembl.org/index.html
GENCODE, https://www.gencodegenes.org/
Online Mendelian Inheritance in Man, https://www.omim.org/
UCSC Genome Browser, https://genome.ucsc.edu/

References

1. O'Sullivan, R.J., and Karlseder, J. (2010). Telomeres: protecting chromosomes against genome instability. Nat. Rev. Mol. Cell Biol. *11*, 171–181.
2. Allsopp, R.C., Vaziri, H., Patterson, C., Goldstein, S., Younglai, E.V., Futcher, A.B., Greider, C.W., and Harley, C.B. (1992). Telomere length predicts replicative capacity of human fibroblasts. Proc. Natl. Acad. Sci. USA *89*, 10114–10118.
3. de Lange, T. (2018). Shelterin-Mediated Telomere Protection. Annu. Rev. Genet. *52*, 223–247.
4. Blackburn, E.H., and Collins, K. (2011). Telomerase: an RNP enzyme synthesizes DNA. Cold Spring Harb. Perspect. Biol. *3*, a003558.
5. Armanios, M., and Blackburn, E.H. (2012). The telomere syndromes. Nat. Rev. Genet. *13*, 693–704.
6. Holohan, B., Wright, W.E., and Shay, J.W. (2014). Cell biology of disease: Telomeropathies: an emerging spectrum disorder. J. Cell Biol. *205*, 289–299.
7. Brouilette, S., Singh, R.K., Thompson, J.R., Goodall, A.H., and Samani, N.J. (2003). White cell telomere length and risk of premature myocardial infarction. Arterioscler. Thromb. Vasc. Biol. *23*, 842–846.
8. Brouilette, S.W., Moore, J.S., McMahon, A.D., Thompson, J.R., Ford, I., Shepherd, J., Packard, C.J., Samani, N.J.; and West of Scotland Coronary Prevention Study Group (2007). Telomere length, risk of coronary heart disease, and statin treatment in the West of Scotland Primary Prevention Study: a nested case-control study. Lancet *369*, 107–114.
9. Benetos, A., Gardner, J.P., Zureik, M., Labat, C., Xiaobin, L., Adamopoulos, C., Temmar, M., Bean, K.E., Thomas, F., and Aviv, A. (2004). Short telomeres are associated with increased carotid atherosclerosis in hypertensive subjects. Hypertension *43*, 182–185.
10. Fitzpatrick, A.L., Kronmal, R.A., Gardner, J.P., Psaty, B.M., Jenny, N.S., Tracy, R.P., Walston, J., Kimura, M., and Aviv, A. (2007). Leukocyte telomere length and cardiovascular disease in the cardiovascular health study. Am. J. Epidemiol. *165*, 14–21.
11. Wentzensen, I.M., Mirabello, L., Pfeiffer, R.M., and Savage, S.A. (2011). The association of telomere length and cancer: a meta-analysis. Cancer Epidemiol. Biomarkers Prev. *20*, 1238–1250.

12. Zhu, X., Han, W., Xue, W., Zou, Y., Xie, C., Du, J., and Jin, G. (2016). The association between telomere length and cancer risk in population studies. Sci. Rep. *6*, 22243.
13. Njajou, O.T., Cawthon, R.M., Damcott, C.M., Wu, S.H., Ott, S., Garant, M.J., Blackburn, E.H., Mitchell, B.D., Shuldiner, A.R., and Hsueh, W.C. (2007). Telomere length is paternally inherited and is associated with parental lifespan. Proc. Natl. Acad. Sci. USA *104*, 12135–12139.
14. Broer, L., Codd, V., Nyholt, D.R., Deelen, J., Mangino, M., Willemsen, G., Albrecht, E., Amin, N., Beekman, M., de Geus, E.J., et al. (2013). Meta-analysis of telomere length in 19,713 subjects reveals high heritability, stronger maternal inheritance and a paternal age effect. Eur. J. Hum. Genet. *21*, 1163–1168.
15. Codd, V., Nelson, C.P., Albrecht, E., Mangino, M., Deelen, J., Buxton, J.L., Hottenga, J.J., Fischer, K., Esko, T., Surakka, I., et al.; CARDIoGRAM consortium (2013). Identification of seven loci affecting mean telomere length and their association with disease. Nat. Genet. *45*, 422–427, e1–e2.
16. Haycock, P.C., Burgess, S., Nounu, A., Zheng, J., Okoli, G.N., Bowden, J., Wade, K.H., Timpson, N.J., Evans, D.M., Willeit, P., et al.; Telomeres Mendelian Randomization Collaboration (2017). Association Between Telomere Length and Risk of Cancer and Non-Neoplastic Diseases: A Mendelian Randomization Study. JAMA Oncol. *3*, 636–651.
17. Zhan, Y., Song, C., Karlsson, R., Tillander, A., Reynolds, C.A., Pedersen, N.L., and Hägg, S. (2015). Telomere Length Shortening and Alzheimer Disease–A Mendelian Randomization Study. JAMA Neurol. *72*, 1202–1203.
18. Zhang, C., Doherty, J.A., Burgess, S., Hung, R.J., Lindström, S., Kraft, P., Gong, J., Amos, C.I., Sellers, T.A., Monteiro, A.N., et al.; GECCO and GAME-ON Network: CORECT, DRIVE, ELLIPSE, FOCI, and TRICL (2015). Genetic determinants of telomere length and risk of common cancers: a Mendelian randomization study. Hum. Mol. Genet. *24*, 5356–5366.
19. Iles, M.M., Bishop, D.T., Taylor, J.C., Hayward, N.K., Brossard, M., Cust, A.E., Dunning, A.M., Lee, J.E., Moses, E.K., Akslen, L.A., et al.; AMFS Investigators; IBD investigators; QMEGA and QTWIN Investigators; SDH Study Group; and GenoMEL Consortium (2014). The effect on melanoma risk of genes previously associated with telomere length. J. Natl. Cancer Inst. *106*, dju267.
20. Codd, V., Mangino, M., van der Harst, P., Braund, P.S., Kaiser, M., Beveridge, A.J., Rafelt, S., Moore, J., Nelson, C., Soranzo, N., et al.; Wellcome Trust Case Control Consortium (2010). Common variants near TERC are associated with mean telomere length. Nat. Genet. *42*, 197–199.
21. Levy, D., Neuhausen, S.L., Hunt, S.C., Kimura, M., Hwang, S.J., Chen, W., Bis, J.C., Fitzpatrick, A.L., Smith, E., Johnson, A.D., et al. (2010). Genome-wide association identifies OBFC1 as a locus involved in human leukocyte telomere biology. Proc. Natl. Acad. Sci. USA *107*, 9293–9298.
22. Pooley, K.A., Bojesen, S.E., Weischer, M., Nielsen, S.F., Thompson, D., Amin Al Olama, A., Michailidou, K., Tyrer, J.P., Benlloch, S., Brown, J., et al. (2013). A genome-wide association scan (GWAS) for mean telomere length within the COGS project: identified loci show little association with hormone-related cancer risk. Hum. Mol. Genet. *22*, 5056–5064.
23. Mangino, M., Christiansen, L., Stone, R., Hunt, S.C., Horvath, K., Eisenberg, D.T., Kimura, M., Petersen, I., Kark, J.D., Herbig, U., et al. (2015). DCAF4, a novel gene associated with leucocyte telomere length. J. Med. Genet. *52*, 157–162.
24. Mangino, M., Hwang, S.J., Spector, T.D., Hunt, S.C., Kimura, M., Fitzpatrick, A.L., Christiansen, L., Petersen, I., Elbers, C.C., Harris, T., et al. (2012). Genome-wide meta-analysis points to CTC1 and ZNF676 as genes regulating telomere homeostasis in humans. Hum. Mol. Genet. *21*, 5385–5394.
25. Delgado, D.A., Zhang, C., Chen, L.S., Gao, J., Roy, S., Shinkle, J., Sabarinathan, M., Argos, M., Tong, L., Ahmed, A., et al. (2018). Genome-wide association study of telomere length among South Asians identifies a second RTEL1 association signal. J. Med. Genet. *55*, 64–71.
26. Cawthon, R.M. (2002). Telomere measurement by quantitative PCR. Nucleic Acids Res. *30*, e47.
27. Cawthon, R.M. (2009). Telomere length measurement by a novel monochrome multiplex quantitative PCR method. Nucleic Acids Res. *37*, e21–e21.
28. Danesh, J., Saracci, R., Berglund, G., Feskens, E., Overvad, K., Panico, S., Thompson, S., Fournier, A., Clavel-Chapelon, F., Canonico, M., et al.; EPIC-Heart (2007). EPIC-Heart: the cardiovascular component of a prospective study of nutritional, lifestyle and biological factors in 520,000 middle-aged participants from 10 European countries. Eur. J. Epidemiol. *22*, 129–141.
29. Langenberg, C., Sharp, S.J., Franks, P.W., Scott, R.A., Deloukas, P., Forouhi, N.G., Froguel, P., Groop, L.C., Hansen, T., Palla, L., et al. (2014). Gene-lifestyle interaction and type 2 diabetes: the EPIC interact case-cohort study. PLoS Med. *11*, e1001647.
30. Mägi, R., and Morris, A.P. (2010). GWAMA: software for genome-wide association meta-analysis. BMC Bioinformatics *11*, 288.
31. Storey, J.D. (2002). A direct approach to false discovery rates. J. R. Statist. Soc. B *64*, 479–498.
32. Yang, J., Lee, S.H., Goddard, M.E., and Visscher, P.M. (2011). GCTA: a tool for genome-wide complex trait analysis. Am. J. Hum. Genet. *88*, 76–82.
33. Yang, J., Ferreira, T., Morris, A.P., Medland, S.E., Madden, P.A., Heath, A.C., Martin, N.G., Montgomery, G.W., Weedon, M.N., Loos, R.J., et al.; Genetic Investigation of ANthropometric Traits (GIANT) Consortium; and DIAbetes Genetics Replication And Meta-analysis (DIAGRAM) Consortium (2012). Conditional and joint multiple-SNP analysis of GWAS summary statistics identifies additional variants influencing complex traits. Nat. Genet. *44*, 369–375, S1–S3.
34. Pruim, R.J., Welch, R.P., Sanna, S., Teslovich, T.M., Chines, P.S., Gliedt, T.P., Boehnke, M., Abecasis, G.R., and Willer, C.J. (2010). LocusZoom: regional visualization of genome-wide association scan results. Bioinformatics *26*, 2336–2337.
35. Wang, K., Li, M., and Hakonarson, H. (2010). ANNOVAR: functional annotation of genetic variants from high-throughput sequencing data. Nucleic Acids Res. *38*, e164.
36. O'Leary, N.A., Wright, M.W., Brister, J.R., Ciufo, S., Haddad, D., McVeigh, R., Rajput, B., Robbertse, B., Smith-White, B., Ako-Adjei, D., et al. (2016). Reference sequence (RefSeq) database at NCBI: current status, taxonomic expansion, and functional annotation. Nucleic Acids Res. *44* (D1), D733–D745.
37. Zerbino, D.R., Achuthan, P., Akanni, W., Amode, M.R., Barrell, D., Bhai, J., Billis, K., Cummins, C., Gall, A., Girón,

C.G., et al. (2018). Ensembl 2018. Nucleic Acids Res. *46* (D1), D754–D761.
38. Harrow, J., Frankish, A., Gonzalez, J.M., Tapanari, E., Diekhans, M., Kokocinski, F., Aken, B.L., Barrell, D., Zadissa, A., Searle, S., et al. (2012). GENCODE: the reference human genome annotation for The ENCODE Project. Genome Res. *22*, 1760–1774.
39. Karolchik, D., Baertsch, R., Diekhans, M., Furey, T.S., Hinrichs, A., Lu, Y.T., Roskin, K.M., Schwartz, M., Sugnet, C.W., Thomas, D.J., et al.; University of California Santa Cruz (2003). The UCSC Genome Browser Database. Nucleic Acids Res. *31*, 51–54.
40. Wang, J., Dayem Ullah, A.Z., and Chelala, C. (2018). IW-Scoring: an Integrative Weighted Scoring framework for annotating and prioritizing genetic variations in the noncoding genome. Nucleic Acids Res. *46*, e47.
41. Barbeira, A.N., Dickinson, S.P., Bonazzola, R., Zheng, J., Wheeler, H.E., Torres, J.M., Torstenson, E.S., Shah, K.P., Garcia, T., Edwards, T.L., et al.; GTEx Consortium (2018). Exploring the phenotypic consequences of tissue specific gene expression variation inferred from GWAS summary statistics. Nat. Commun. *9*, 1825.
42. Gamazon, E.R., Wheeler, H.E., Shah, K.P., Mozaffari, S.V., Aquino-Michaels, K., Carroll, R.J., Eyler, A.E., Denny, J.C., Nicolae, D.L., Cox, N.J., Im, H.K.; and GTEx Consortium (2015). A gene-based association method for mapping traits using reference transcriptome data. Nat. Genet. *47*, 1091–1098.
43. Fortune, M.D., Guo, H., Burren, O., Schofield, E., Walker, N.M., Ban, M., Sawcer, S.J., Bowes, J., Worthington, J., Barton, A., et al. (2015). Statistical colocalization of genetic risk variants for related autoimmune diseases in the context of common controls. Nat. Genet. *47*, 839–846.
44. Battle, A., Brown, C.D., Engelhardt, B.E., Montgomery, S.B.; GTEx Consortium; Laboratory, Data Analysis &Coordinating Center (LDACC)—Analysis Working Group; Statistical Methods groups—Analysis Working Group; Enhancing GTEx (eGTEx) groups; NIH Common Fund; NIH/NCI; NIH/NHGRI; NIH/NIMH; NIH/NIDA; Biospecimen Collection Source Site—NDRI; Biospecimen Collection Source Site—RPCI-Biospecimen Core Resource—VARI; Brain Bank Repository—University of Miami Brain Endowment Bank; Leidos Biomedical—Project Management; ELSI Study; Genome Browser Data Integration &Visualization—EBI; Genome Browser Data Integration &Visualization—UCSC Genomics Institute, University of California Santa Cruz; Lead analysts; Laboratory, Data Analysis &Coordinating Center (LDACC); NIH program management; Biospecimen collection; Pathology; and eQTL manuscript working group (2017). Genetic effects on gene expression across human tissues. Nature *550*, 204–213.
45. Giambartolomei, C., Vukcevic, D., Schadt, E.E., Franke, L., Hingorani, A.D., Wallace, C., and Plagnol, V. (2014). Bayesian test for colocalisation between pairs of genetic association studies using summary statistics. PLoS Genet. *10*, e1004383.
46. Guo, H., Fortune, M.D., Burren, O.S., Schofield, E., Todd, J.A., and Wallace, C. (2015). Integration of disease association and eQTL data using a Bayesian colocalisation approach highlights six candidate causal genes in immune-mediated diseases. Hum. Mol. Genet. *24*, 3305–3313.
47. Jin, Y., Andersen, G., Yorgov, D., Ferrara, T.M., Ben, S., Brownson, K.M., Holland, P.J., Birlea, S.A., Siebert, J., Hartmann, A., et al. (2016). Genome-wide association studies of autoimmune vitiligo identify 23 new risk loci and highlight key pathways and regulatory variants. Nat. Genet. *48*, 1418–1424.
48. Bonder, M.J., Luijk, R., Zhernakova, D.V., Moed, M., Deelen, P., Vermaat, M., van Iterson, M., van Dijk, F., van Galen, M., Bot, J., et al.; BIOS Consortium (2017). Disease variants alter transcription factor levels and methylation of their binding sites. Nat. Genet. *49*, 131–138.
49. Chen, L., Ge, B., Casale, F.P., Vasquez, L., Kwan, T., Garrido-Martín, D., Watt, S., Yan, Y., Kundu, K., Ecker, S., et al. (2016). Genetic Drivers of Epigenetic and Transcriptional Variation in Human Immune Cells. Cell *167*, 1398–1414.e24.
50. Gaunt, T.R., Shihab, H.A., Hemani, G., Min, J.L., Woodward, G., Lyttleton, O., Zheng, J., Duggirala, A., McArdle, W.L., Ho, K., et al. (2016). Systematic identification of genetic influences on methylation across the human life course. Genome Biol. *17*, 61.
51. Gusev, A., Ko, A., Shi, H., Bhatia, G., Chung, W., Penninx, B.W.J.H., Jansen, R., de Geus, E.J.C., Boomsma, D.I., Wright, F.A., et al. (2016). Integrative approaches for large-scale transcriptome-wide association studies. Nat. Genet. *48*, 245–252.
52. Wright, D.J., Day, F.R., Kerrison, N.D., Zink, F., Cardona, A., Sulem, P., Thompson, D.J., Sigurjonsdottir, S., Gudbjartsson, D.F., Helgason, A., et al. (2017). Genetic variants associated with mosaic Y chromosome loss highlight cell cycle genes and overlap with cancer susceptibility. Nat. Genet. *49*, 674–679.
53. Mi, H., Muruganujan, A., Ebert, D., Huang, X., and Thomas, P.D. (2019). PANTHER version 14: more genomes, a new PANTHER GO-slim and improvements in enrichment analysis tools. Nucleic Acids Res. *47* (D1), D419–D426.
54. Pers, T.H., Karjalainen, J.M., Chan, Y., Westra, H.J., Wood, A.R., Yang, J., Lui, J.C., Vedantam, S., Gustafsson, S., Esko, T., et al.; Genetic Investigation of ANthropometric Traits (GIANT) Consortium (2015). Biological interpretation of genome-wide association studies using predicted gene functions. Nat. Commun. *6*, 5890.
55. Ashburner, M., Ball, C.A., Blake, J.A., Botstein, D., Butler, H., Cherry, J.M., Davis, A.P., Dolinski, K., Dwight, S.S., Eppig, J.T., et al.; The Gene Ontology Consortium (2000). Gene ontology: tool for the unification of biology. Nat. Genet. *25*, 25–29.
56. Kanehisa, M., Goto, S., Sato, Y., Furumichi, M., and Tanabe, M. (2012). KEGG for integration and interpretation of large-scale molecular data sets. Nucleic Acids Res. *40*, D109–D114.
57. Croft, D., O'Kelly, G., Wu, G., Haw, R., Gillespie, M., Matthews, L., Caudy, M., Garapati, P., Gopinath, G., Jassal, B., et al. (2011). Reactome: a database of reactions, pathways and biological processes. Nucleic Acids Res. *39*, D691–D697.
58. Lage, K., Karlberg, E.O., Størling, Z.M., Olason, P.I., Pedersen, A.G., Rigina, O., Hinsby, A.M., Tümer, Z., Pociot, F., Tommerup, N., et al. (2007). A human phenome-interactome network of protein complexes implicated in genetic disorders. Nat. Biotechnol. *25*, 309–316.
59. Blake, J.A., Eppig, J.T., Kadin, J.A., Richardson, J.E., Smith, C.L., Bult, C.J.; and the Mouse Genome Database Group (2017). Mouse Genome Database (MGD)-2017: community knowledge resource for the laboratory mouse. Nucleic Acids Res. *45* (D1), D723–D729.

60. Shannon, P., Markiel, A., Ozier, O., Baliga, N.S., Wang, J.T., Ramage, D., Amin, N., Schwikowski, B., and Ideker, T. (2003). Cytoscape: a software environment for integrated models of biomolecular interaction networks. Genome Res. *13*, 2498–2504.
61. Burgess, S., Butterworth, A., and Thompson, S.G. (2013). Mendelian randomization analysis with multiple genetic variants using summarized data. Genet. Epidemiol. *37*, 658–665.
62. Sudlow, C., Gallacher, J., Allen, N., Beral, V., Burton, P., Danesh, J., Downey, P., Elliott, P., Green, J., Landray, M., et al. (2015). UK biobank: an open access resource for identifying the causes of a wide range of complex diseases of middle and old age. PLoS Med. *12*, e1001779.
63. Marchini, J., Howie, B., Myers, S., McVean, G., and Donnelly, P. (2007). A new multipoint method for genome-wide association studies by imputation of genotypes. Nat. Genet. *39*, 906–913.
64. Bowden, J., Davey Smith, G., Haycock, P.C., and Burgess, S. (2016). Consistent Estimation in Mendelian Randomization with Some Invalid Instruments Using a Weighted Median Estimator. Genet. Epidemiol. *40*, 304–314.
65. Zhao, Q., Wang, J., Hemani, G., Bowden, J., and Small, D.S. (2018). Statistical inference in two-sample summary-data Mendelian randomization using robust adjusted profile score. arXiv:1801.09652.
66. Bowden, J., Davey Smith, G., and Burgess, S. (2015). Mendelian randomization with invalid instruments: effect estimation and bias detection through Egger regression. Int. J. Epidemiol. *44*, 512–525.
67. Hemani, G., Tilling, K., and Davey Smith, G. (2017). Orienting the causal relationship between imprecisely measured traits using GWAS summary data. PLoS Genet. *13*, e1007081.
68. Zheng, J., Erzurumluoglu, A.M., Elsworth, B.L., Kemp, J.P., Howe, L., Haycock, P.C., Hemani, G., Tansey, K., Laurin, C., Pourcain, B.S., et al.; Early Genetics and Lifecourse Epidemiology (EAGLE) Eczema Consortium (2017). LD Hub: a centralized database and web interface to perform LD score regression that maximizes the potential of summary level GWAS data for SNP heritability and genetic correlation analysis. Bioinformatics *33*, 272–279.
69. Staley, J.R., Blackshaw, J., Kamat, M.A., Ellis, S., Surendran, P., Sun, B.B., Paul, D.S., Freitag, D., Burgess, S., Danesh, J., et al. (2016). PhenoScanner: a database of human genotype-phenotype associations. Bioinformatics *32*, 3207–3209.
70. Dorajoo, R., Chang, X., Gurung, R.L., Li, Z., Wang, L., Wang, R., Beckman, K.B., Adams-Haduch, J., M, Y., Liu, S., et al. (2019). Loci for human leukocyte telomere length in the Singaporean Chinese population and trans-ethnic genetic studies. Nat. Commun. *10*, 2491.
71. Nelson, C.P., Goel, A., Butterworth, A.S., Kanoni, S., Webb, T.R., Marouli, E., Zeng, L., Ntalla, I., Lai, F.Y., Hopewell, J.C., et al.; EPIC-CVD Consortium; CARDIoGRAMplusC4D; and UK Biobank CardioMetabolic Consortium CHD working group (2017). Association analyses based on false discovery rate implicate new loci for coronary artery disease. Nat. Genet. *49*, 1385–1391.
72. Wang, X.G., Wang, Z.Q., Tong, W.M., and Shen, Y. (2007). PARP1 Val762Ala polymorphism reduces enzymatic activity. Biochem. Biophys. Res. Commun. *354*, 122–126.
73. Beneke, S., Cohausz, O., Malanga, M., Boukamp, P., Althaus, F., and Bürkle, A. (2008). Rapid regulation of telomere length is mediated by poly(ADP-ribose) polymerase-1. Nucleic Acids Res. *36*, 6309–6317.
74. Gomez, M., Wu, J., Schreiber, V., Dunlap, J., Dantzer, F., Wang, Y., and Liu, Y. (2006). PARP1 Is a TRF2-associated poly(ADP-ribose)polymerase and protects eroded telomeres. Mol. Biol. Cell *17*, 1686–1696.
75. Lee, J., and Zhou, P. (2007). DCAFs, the missing link of the CUL4-DDB1 ubiquitin ligase. Mol. Cell *26*, 775–780.
76. Garvin, A.J., Densham, R.M., Blair-Reid, S.A., Pratt, K.M., Stone, H.R., Weekes, D., Lawrence, K.J., and Morris, J.R. (2013). The deSUMOylase SENP7 promotes chromatin relaxation for homologous recombination DNA repair. EMBO Rep. *14*, 975–983.
77. Liu, S., Chu, J., Yucer, N., Leng, M., Wang, S.Y., Chen, B.P., Hittelman, W.N., and Wang, Y. (2011). RING finger and WD repeat domain 3 (RFWD3) associates with replication protein A (RPA) and facilitates RPA-mediated DNA damage response. J. Biol. Chem. *286*, 22314–22322.
78. Bartocci, C., Diedrich, J.K., Ouzounov, I., Li, J., Piunti, A., Pasini, D., Yates, J.R., 3rd, and Lazzerini Denchi, E. (2014). Isolation of chromatin from dysfunctional telomeres reveals an important role for Ring1b in NHEJ-mediated chromosome fusions. Cell Rep. *7*, 1320–1332.
79. Arnoult, N., and Karlseder, J. (2015). Complex interactions between the DNA-damage response and mammalian telomeres. Nat. Struct. Mol. Biol. *22*, 859–866.
80. Knies, K., Inano, S., Ramírez, M.J., Ishiai, M., Surrallés, J., Takata, M., and Schindler, D. (2017). Biallelic mutations in the ubiquitin ligase RFWD3 cause Fanconi anemia. J. Clin. Invest. *127*, 3013–3027.
81. Krenciute, G., Liu, S., Yucer, N., Shi, Y., Ortiz, P., Liu, Q., Kim, B.J., Odejimi, A.O., Leng, M., Qin, J., and Wang, Y. (2013). Nuclear BAG6-UBL4A-GET4 complex mediates DNA damage signaling and cell death. J. Biol. Chem. *288*, 20547–20557.
82. Kim, M.K., Kang, M.R., Nam, H.W., Bae, Y.S., Kim, Y.S., and Chung, I.K. (2008). Regulation of telomeric repeat binding factor 1 binding to telomeres by casein kinase 2-mediated phosphorylation. J. Biol. Chem. *283*, 14144–14152.
83. Franzolin, E., Pontarin, G., Rampazzo, C., Miazzi, C., Ferraro, P., Palumbo, E., Reichard, P., and Bianchi, V. (2013). The deoxynucleotide triphosphohydrolase SAMHD1 is a major regulator of DNA precursor pools in mammalian cells. Proc. Natl. Acad. Sci. USA *110*, 14272–14277.
84. Jobert, L., Skjeldam, H.K., Dalhus, B., Galashevskaya, A., Vågbø, C.B., Bjørås, M., and Nilsen, H. (2013). The human base excision repair enzyme SMUG1 directly interacts with DKC1 and contributes to RNA quality control. Mol. Cell *49*, 339–345.
85. Irwin, C.R., Hitt, M.M., and Evans, D.H. (2017). Targeting Nucleotide Biosynthesis: A Strategy for Improving the Oncolytic Potential of DNA Viruses. Front. Oncol. *7*, 229.
86. Reichard, P. (1988). Interactions between deoxyribonucleotide and DNA synthesis. Annu. Rev. Biochem. *57*, 349–374.
87. Bebenek, K., Roberts, J.D., and Kunkel, T.A. (1992). The effects of dNTP pool imbalances on frameshift fidelity during DNA replication. J. Biol. Chem. *267*, 3589–3596.
88. Ojha, J., Codd, V., Nelson, C.P., Samani, N.J., Smirnov, I.V., Madsen, N.R., Hansen, H.M., de Smith, A.J., Bracci, P.M., Wiencke, J.K., et al.; ENGAGE Consortium Telomere Group (2016). Genetic Variation Associated with Longer Telomere Length Increases Risk of Chronic Lymphocytic Leukemia. Cancer Epidemiol. Biomarkers Prev. *25*, 1043–1049.

89. Córdoba-Lanús, E., Cazorla-Rivero, S., Espinoza-Jiménez, A., de-Torres, J.P., Pajares, M.J., Aguirre-Jaime, A., Celli, B., and Casanova, C. (2017). Telomere shortening and accelerated aging in COPD: findings from the BODE cohort. Respir. Res. *18*, 59.
90. Kurz, D.J., Kloeckener-Gruissem, B., Akhmedov, A., Eberli, F.R., Bühler, I., Berger, W., Bertel, O., and Lüscher, T.F. (2006). Degenerative aortic valve stenosis, but not coronary disease, is associated with shorter telomere length in the elderly. Arterioscler. Thromb. Vasc. Biol. *26*, e114–e117.
91. Steer, S.E., Williams, F.M., Kato, B., Gardner, J.P., Norman, P.J., Hall, M.A., Kimura, M., Vaughan, R., Aviv, A., and Spector, T.D. (2007). Reduced telomere length in rheumatoid arthritis is independent of disease activity and duration. Ann. Rheum. Dis. *66*, 476–480.
92. van der Harst, P., van der Steege, G., de Boer, R.A., Voors, A.A., Hall, A.S., Mulder, M.J., van Gilst, W.H., van Veldhuisen, D.J.; and MERIT-HF Study Group (2007). Telomere length of circulating leukocytes is decreased in patients with chronic heart failure. J. Am. Coll. Cardiol. *49*, 1459–1464.
93. Tong, A.S., Stern, J.L., Sfeir, A., Kartawinata, M., de Lange, T., Zhu, X.D., and Bryan, T.M. (2015). ATM and ATR Signaling Regulate the Recruitment of Human Telomerase to Telomeres. Cell Rep. *13*, 1633–1646.
94. Denchi, E.L., and de Lange, T. (2007). Protection of telomeres through independent control of ATM and ATR by TRF2 and POT1. Nature *448*, 1068–1071.
95. Egan, E.D., and Collins, K. (2012). Biogenesis of telomerase ribonucleoproteins. RNA *18*, 1747–1759.
96. Nguyen, D., Grenier St-Sauveur, V., Bergeron, D., Dupuis-Sandoval, F., Scott, M.S., and Bachand, F. (2015). A Polyadenylation-Dependent 3′ End Maturation Pathway Is Required for the Synthesis of the Human Telomerase RNA. Cell Rep. *13*, 2244–2257.
97. Boyraz, B., Moon, D.H., Segal, M., Muosieyiri, M.Z., Aykanat, A., Tai, A.K., Cahan, P., and Agarwal, S. (2016). Posttranscriptional manipulation of TERC reverses molecular hallmarks of telomere disease. J. Clin. Invest. *126*, 3377–3382.
98. Schilders, G., Raijmakers, R., Raats, J.M.H., and Pruijn, G.J.M. (2005). MPP6 is an exosome-associated RNA-binding protein involved in 5.8S rRNA maturation. Nucleic Acids Res. *33*, 6795–6804.
99. Austin, W.R., Armijo, A.L., Campbell, D.O., Singh, A.S., Hsieh, T., Nathanson, D., Herschman, H.R., Phelps, M.E., Witte, O.N., Czernin, J., and Radu, C.G. (2012). Nucleoside salvage pathway kinases regulate hematopoiesis by linking nucleotide metabolism with replication stress. J. Exp. Med. *209*, 2215–2228.
100. Davidson, M.B., Katou, Y., Keszthelyi, A., Sing, T.L., Xia, T., Ou, J., Vaisica, J.A., Thevakumaran, N., Marjavaara, L., Myers, C.L., et al. (2012). Endogenous DNA replication stress results in expansion of dNTP pools and a mutator phenotype. EMBO J. *31*, 895–907.

The American Journal of Human Genetics, Volume *106*

Supplemental Data

Genome-wide Association Analysis in Humans Links Nucleotide Metabolism to Leukocyte Telomere Length

Chen Li, Svetlana Stoma, Luca A. Lotta, Sophie Warner, Eva Albrecht, Alessandra Allione, Pascal P. Arp, Linda Broer, Jessica L. Buxton, Alexessander Da Silva Couto Alves, Joris Deelen, Iryna O. Fedko, Scott D. Gordon, Tao Jiang, Robert Karlsson, Nicola Kerrison, Taylor K. Loe, Massimo Mangino, Yuri Milaneschi, Benjamin Miraglio, Natalia Pervjakova, Alessia Russo, Ida Surakka, Ashley van der Spek, Josine E. Verhoeven, Najaf Amin, Marian Beekman, Alexandra I. Blakemore, Federico Canzian, Stephen E. Hamby, Jouke-Jan Hottenga, Peter D. Jones, Pekka Jousilahti, Reedik Mägi, Sarah E. Medland, Grant W. Montgomery, Dale R. Nyholt, Markus Perola, Kirsi H. Pietiläinen, Veikko Salomaa, Elina Sillanpää, H. Eka Suchiman, Diana van Heemst, Gonneke Willemsen, Antonio Agudo, Heiner Boeing, Dorret I. Boomsma, Maria-Dolores Chirlaque, Guy Fagherazzi, Pietro Ferrari, Paul Franks, Christian Gieger, Johan Gunnar Eriksson, Marc Gunter, Sara Hägg, Iiris Hovatta, Liher Imaz, Jaakko Kaprio, Rudolf Kaaks, Timothy Key, Vittorio Krogh, Nicholas G. Martin, Olle Melander, Andres Metspalu, Concha Moreno, N. Charlotte Onland-Moret, Peter Nilsson, Ken K. Ong, Kim Overvad, Domenico Palli, Salvatore Panico, Nancy L. Pedersen, Brenda W.J. H. Penninx, J. Ramón Quirós, Marjo Riitta Jarvelin, Miguel Rodríguez-Barranco, Robert A. Scott, Gianluca Severi, P. Eline Slagboom, Tim D. Spector, Anne Tjonneland, Antonia Trichopoulou, Rosario Tumino, André G. Uitterlinden, Yvonne T. van der Schouw, Cornelia M. van Duijn, Elisabete Weiderpass, Eros Lazzerini Denchi, Giuseppe Matullo, Adam S. Butterworth, John Danesh, Nilesh J. Samani, Nicholas J. Wareham, Christopher P. Nelson, Claudia Langenberg, and Veryan Codd

Supplemental Data

Supplemental Figure 1. Study design. Schematic graph to illustrate study design of the LTL GWAS meta-analysis. GWAS was conducted in each individual study cohort, stratified by genotyping platform and disease status. SNP genotyping, GWAS and meta-analyses as well as the corresponding QC procedures are described within the methods.

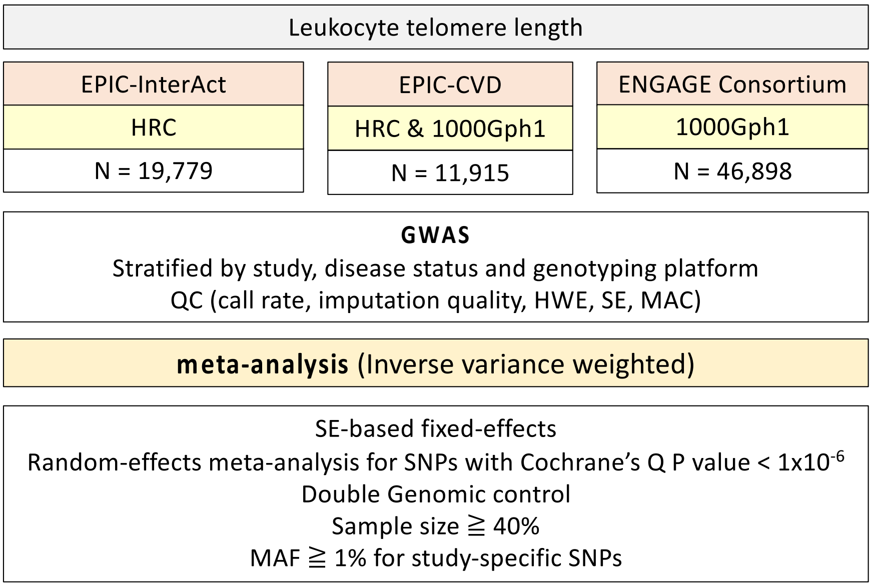

Supplemental Figure 2. Manhattan Plot of GWAS results. Manhattan plot with quantile-quantile plot inlay. Known loci were labelled in blue, novel loci associated with LTL at genome-wide significance (p-value<5x10^{-8}, red line) in red, and at FDR threshold of 5% (blue line) in orange.

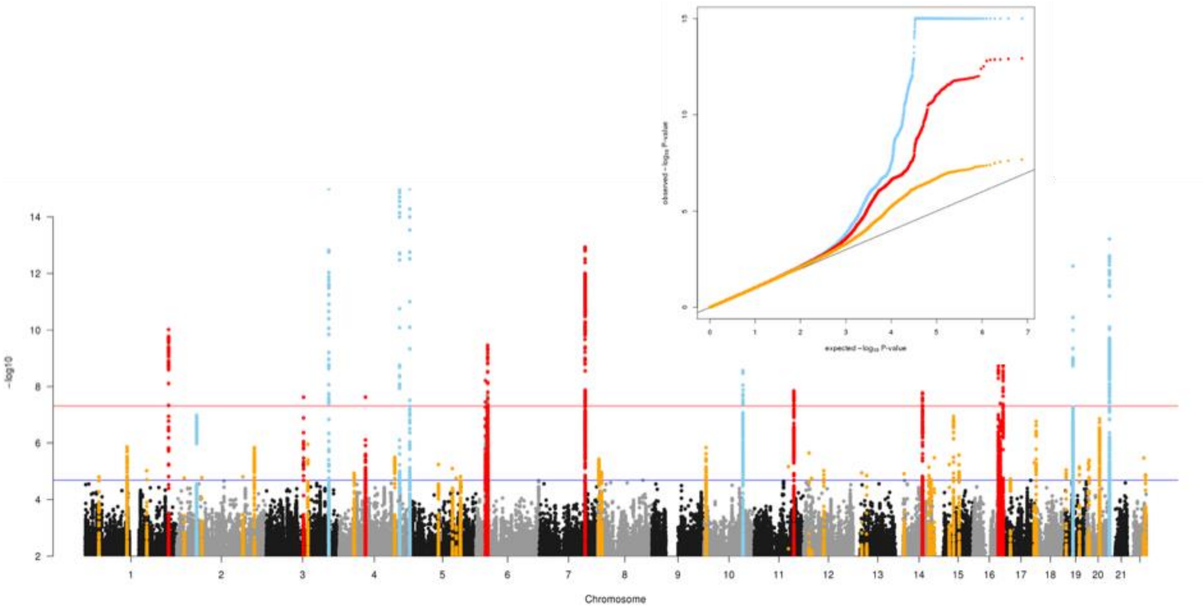

Supplementary Figure 3. Regional plots of genome-wide significant loci ($p<5x10^{-8}$). For all loci 400kb windows encompassing conditionally independent variants, except the TERT locus which is illustrated as a 200kb window.

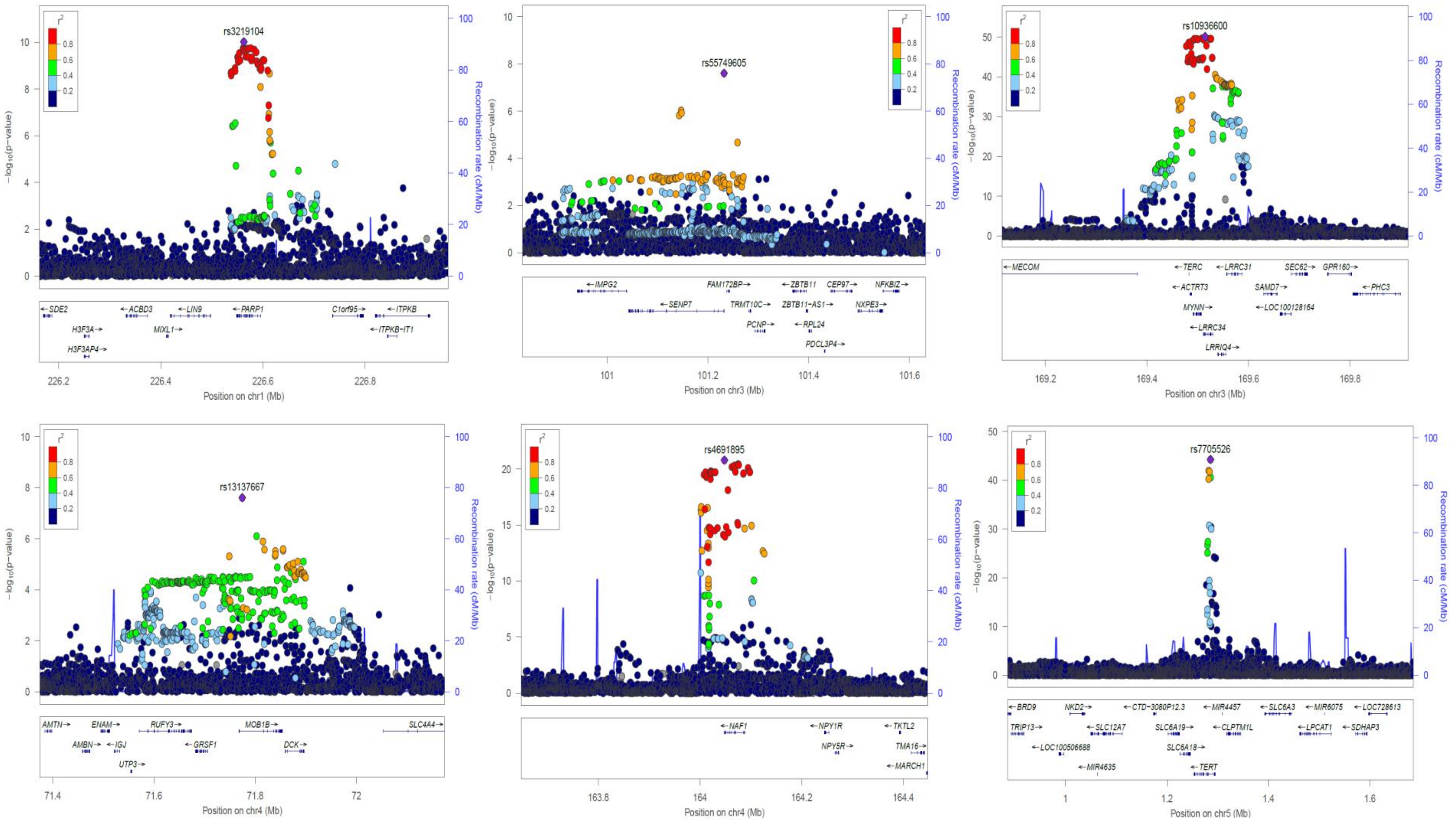

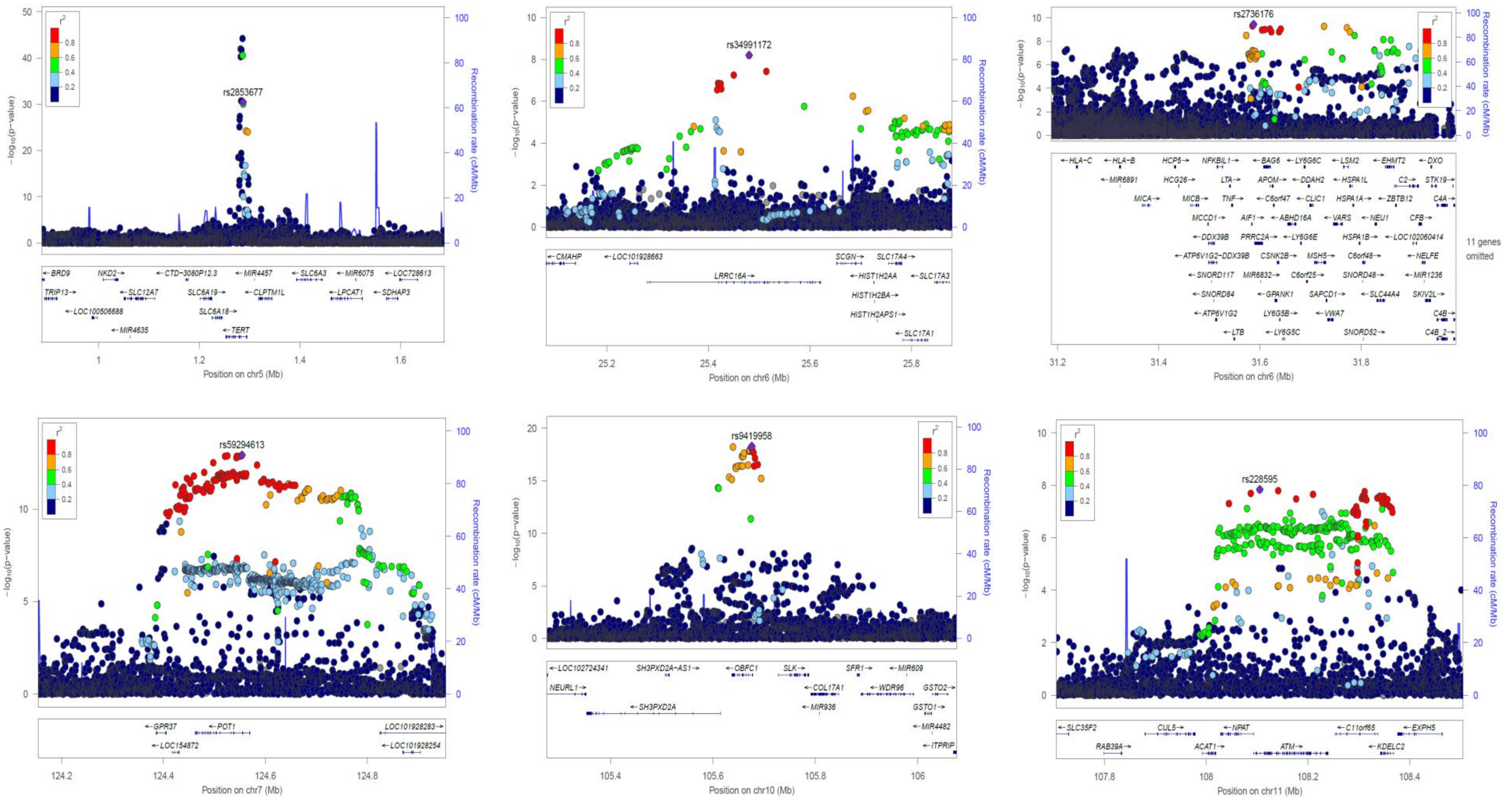

rs2853677
Position on chr5 (Mb)
rs34991172
Position on chr6 (Mb)
rs2736176
Position on chr6 (Mb)
11 genes omitted
rs59294613
Position on chr7 (Mb)
rs9419958
Position on chr10 (Mb)
rs228595
Position on chr11 (Mb)
$-\log_{10}$(p-value)
Recombination rate (cM/Mb)
r^2
TERT
CLPTM1L
LRRC16A
POT1
OBFC1
ATM
NPAT

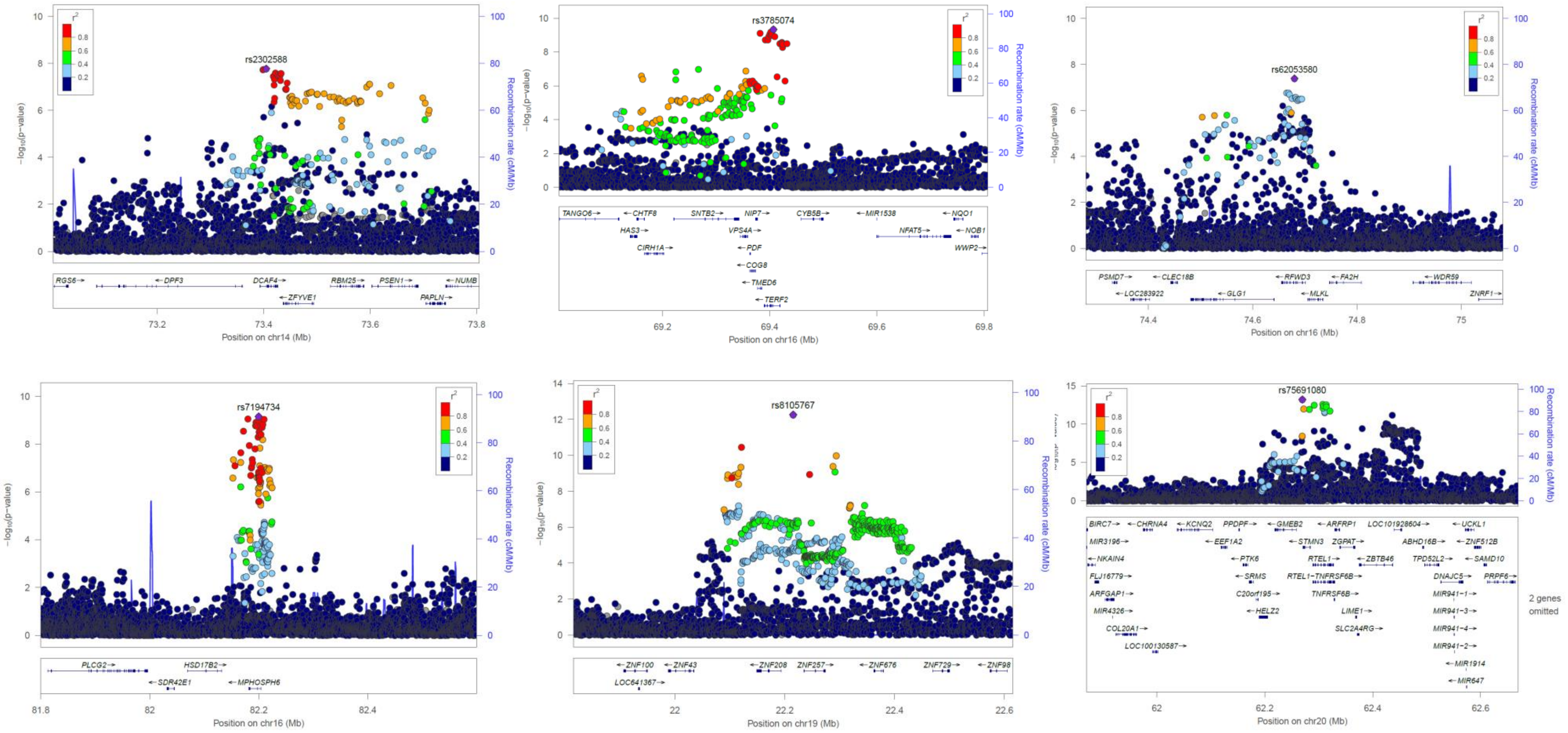

rs2302588
−log10(p−value)
Recombination rate (cM/Mb)
RGS6
DPF3
DCAF4
ZFYVE1
RBM25
PSEN1
NUMB
PAPLN
Position on chr14 (Mb)
rs3785074
TANGO6
CHTF8
HAS3
CIRH1A
SNTB2
NIP7
VPS4A
PDF
COG8
TMED6
TERF2
CYB5B
MIR1538
NFAT5
NQO1
NOB1
WWP2
Position on chr16 (Mb)
rs62053580
PSMD7
LOC283922
CLEC18B
GLG1
RFWD3
MLKL
FA2H
WDR59
ZNRF1
Position on chr16 (Mb)
rs7194734
PLCG2
SDR42E1
HSD17B2
MPHOSPH6
Position on chr16 (Mb)
rs8105767
ZNF100
LOC641367
ZNF43
ZNF208
ZNF257
ZNF676
ZNF729
ZNF98
Position on chr19 (Mb)
rs75691080
BIRC7
MIR3196
NKAIN4
FLJ16779
ARFGAP1
MIR4326
COL20A1
LOC100130587
CHRNA4
KCNQ2
EEF1A2
PPDPF
PTK6
SRMS
C20orf195
HELZ2
GMEB2
STMN3
RTEL1
RTEL1−TNFRSF6B
TNFRSF6B
ARFRP1
ZGPAT
LIME1
SLC2A4RG
ZBTB46
LOC101928604
ABHD16B
TPD52L2
DNAJC5
MIR941−1
MIR941−3
MIR941−4
MIR941−2
MIR1914
MIR647
UCKL1
ZNF512B
SAMD10
PRPF6
2 genes omitted
Position on chr20 (Mb)

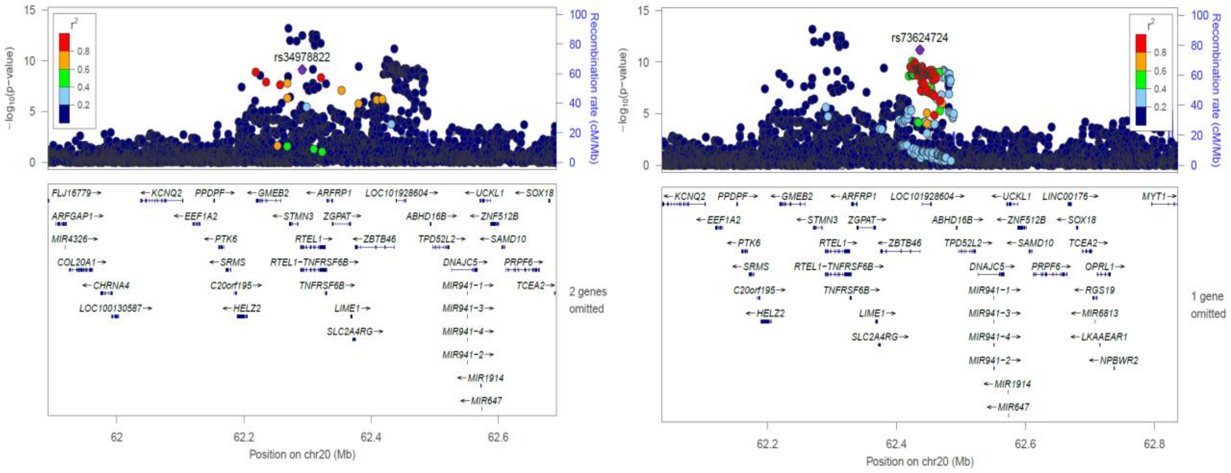

rs34978822
rs73624724
−log10(p−value)
Recombination rate (cM/Mb)
r2
Position on chr20 (Mb)
2 genes omitted
1 gene omitted
FLJ16779
KCNQ2
PPDPF
GMEB2
ARFRP1
LOC101928604
UCKL1
SOX18
ARFGAP1
EEF1A2
STMN3
ZGPAT
ABHD16B
ZNF512B
MIR4326
PTK6
RTEL1
ZBTB46
TPD52L2
SAMD10
COL20A1
SRMS
RTEL1−TNFRSF6B
DNAJC5
PRPF6
CHRNA4
C20orf195
TNFRSF6B
MIR941−1
TCEA2
LOC100130587
HELZ2
LIME1
MIR941−3
SLC2A4RG
MIR941−4
MIR941−2
MIR1914
MIR647
LINC00176
MYT1
OPRL1
RGS19
MIR6813
LKAAEAR1
NPBWR2

Supplementary Figure 4. Mendelian randomisation results for the effect of shorter LTL on the risk of 122 diseases in UK Biobank. MR analysis was repeating using only SNPs that reached genome-wide significance ($P<5x10^{-8}$). Data shown are odds ratios and 95% confidence intervals for a 1 standard deviation shorter LTL. Diseases are classified into groups as indicated by the boxing and sorted alphabetically within disease group. Nominally significant (P<0.05) associations estimated via inverse-variance weighted Mendelian randomisation are shown in green for a reduction in risk and purple for an increase in risk due to shorter LTL. Where ^{O} indicates nominal (P<0.05) evidence of pleiotropy estimated by MR-Eggers intercept. Full results are also shown in Table S16 along with the full MR sensitivity analysis.

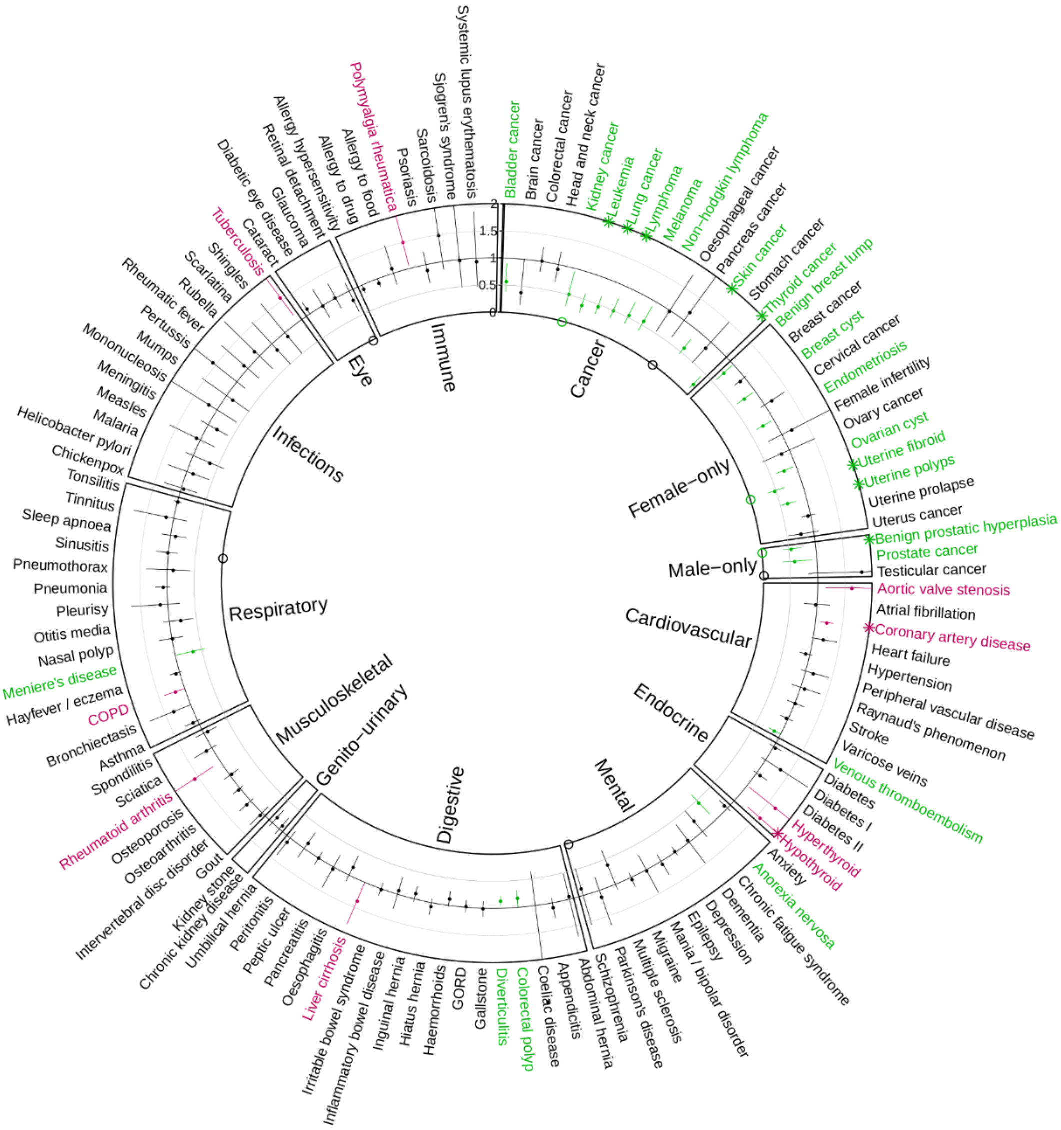

Supplemental Methods

Information on study cohorts

The demographic characteristics of all study cohorts, for both discovery and replication phases are shown in Table S1. All individuals included in the analysis are of European descent.

ENGAGE

The majority of the studies included have previously been described[1]. In addition to these the following studies were included in this analysis.

GENMETS

GENMETS is a subcohort of the Finnish population-based Health 2000 study, comprising of metablic syndrome cases and controls. This cohort is described in more detail elsewhere[2].

NESDA

The Netherlands Study of Depression and Anxiety (NESDA) is an ongoing cohort study into the long- term course and consequences of depressive and anxiety disorders. A description of the study rationale, design, and methods is given elsewhere[3]. Briefly, in 2004 to 2007, participants aged 18 to 65 years were recruited from the community (19%), general practice (54%), and secondary mental health care (27%), therefore reflecting various settings and developmental stages of psychopathology to obtain a full and generalizable picture of the course of psychiatric disorders. A total of 2981 participants were included, consisting of persons with a current or past depressive and/or anxiety disorder and healthy control subjects. Exclusion criteria were a clinically overt primary diagnosis of psychotic, obsessive compulsive, bipolar, or severe addiction disorder and not being fluent in Dutch. The research protocol was approved by the ethical committee of participating universities, and all respondents provided written informed consent.

ROTTERDAM

The Rotterdam Study is a population-based cohort study that investigates the occurrence and determinants of diseases in the elderly, which has been ongoing since 1990[4]. As of 2008, detailed phenotypic and genetic data has been collected on ~15,000 subjects aged 45 years or over. For this study the RS-I and RS-III cohorts were used. The Medical Ethics Committee at Erasmus Medical Center approved the study protocol.

EPIC-InterAct case-cohort study

The EPIC-InterAct study aimed to investigate the independent and interactive effects of genetic and behavioural risk factors on type 2 diabetes risk[5,6]. EPIC-InterAct is a case-cohort study nested within 8 of the 10 countries participating in the EPIC-Europe cohort study. EPIC-InterAct ascertained 12,403 cases of type 2 diabetes from a total cohort of 340,234 participants who provided blood samples at baseline and were followed-up for an average of 7 years (~4 million years of follow-up. Cases were ascertained from multiple data sources including self-report of a physician diagnosis of diabetes, linkage to primary/secondary care records, medication use, hospital admission data and death registration data. We also established a random sub-cohort of 16,154 participants who were representative of participants within each country. By design there is an overlap with the set of incident diabetes cases (n=778). Participant characteristics have been previously reported in detail[5,6]. Observational statistics of LTL, genotyping and imputation are summarised in the Table S1 and Table S2.

EPIC-CVD case-cohort study

EPIC-CVD was designed as a case-cohort study that uses the same random sub-cohort as InterAct, with a focus on incident coronary heart disease and stroke events[7]. The participants included in this analysis are thus incident cases only (7722 coronary heart disease cases and 3451 cerebrovascular disease cases). We also included an additional 752 participants as a random sub-cohort from the two countries not included in EPIC-InterAct (Greece and Norway). Detailed characteristics of the EPIC-CVD participants has been previously reported[8].

Telomere length measurements

Telomere length measurements were performed using an established quantitative PCR technique[9] across 6 laboratories. Laboratory specific information is given below and in Table S1. Details of the techniques used within Helsinki, Leicester and London have been given elsewhere[1].

<u>NESDA</u>: Fasting blood was drawn from participants in the morning between 8:30 and 9:30 am and blood samples were stored in a -80°C freezer afterwards. Leukocyte TL was determined at the laboratory of Telomere Diagnostics, Inc. (Menlo Park, CA, USA), using quantitative polymerase chain reaction (qPCR), adapted from the published original method[9]. Telomere sequence copy number in each patient's sample (T) was compared to a single-copy gene copy number (S), relative to a reference sample. The detailed method is described elsewhere[10].

<u>Rotterdam</u>: Telomere length was measured using a qPCR assay based on the method described elsewhere[9] with minor modifications. For each sample the telomere and 36B4 assay were run in separate wells but in the same 384 wells PCR plate. Each reaction contained 5 ng DNA, 1 uM of each of the telomere primers (tel1b-forward: GGTTTGTTTGGGTTTGGGTTTGGGTTTGGGTTTGGGTT, tel2b-reverse: GGCTTGCCTTACCCTTACCCTTACCCTTACCCTTACCCT) or 250 nM of the 34B4 primers (36B4u-forward: CAGCAAGTGGGAAGGTGTAATCC, 36B4d-reverse:

CCCATTCTATCATCAACGGGTACAA) and 1x Quantifast SYBR green PCR Mastermix (Qiagen). The reactions for both assays were performed in duplicate for each sample in a 7900HT machine (Applied Biosystems). Ct values and PCR efficiencies were calculated per plate using the MINER algorithm[11]. Duplicate Ct values that had a Coefficient of Variance (CV) of more than 1% were excluded from further analysis. Using the average Ct value per sample and the average PCR efficiency per plate the samples were quantified using the formula Q=1/(1+PCR eff)^Ct. The relative telomere length was calculated by dividing the Q of the telomere assay by the Q of the 34B4 assay. To validate the assay 96 random samples were run twice and the CV of that experiment was 4.5%.

Cambridge: Relative mean LTL was measured using a ViiATM Real-Time quantitative PCR system (ThermoFisher Sicentific, Inc), and expressed as a ratio (T/S) of the relative quantities of the telomeric TTAGGG repeat (T) and the single copy of a housekeeping gene, *Albumin* (S). The denominator determines total genome copies per sample, controlling the technical errors during quantification. The measurement was validated by the Terminal Restriction Fragment (TRF) analysis (the “gold standard” measurement of TL) using separate DNA samples extracted from peripheral blood mononuclear cells in 30 individuals (Pearson’s r =0.69). Batch effect was corrected by normalising all the other batches to the fourth batch. Each sample was measured repetitively for three times within one batch, when the same sample was measured in more than one batch, measurement from the last batch was kept for the sample. Samples with coefficients of variation greater than 10% were excluded.

Description of Individual loci associated with LTL

Chr1p13.2. The lead SNP (rs12065882) and three high LD variants are all located within introns of *MAGI3* (*membrane associated guanylate kinase, WW and PDZ domain containing 3*). *MAGI3* has been proposed to act as a tumour suppressor; it regulates cell proliferation in glioma via wnt/β-catenin signalling and interacts with PTEN[12,13]. Both S-PrediXcan and COLOC analyses give evidence to support expression of *AP4B1* (*adaptor related protein complex 4 subunit beta 1*) being influenced by the associated variants. This gene encodes a subunit of a heterotetrametric adapter-like complex 4 that involves in Golgi-associated and lysosomal vesicle biogenesis and membrane trafficking, transporting proteins from the trans-Golgi network to the endosomal-lysosomal system[14,15]. Mutations in this gene are associated with an autosomal recessively inherited disease, spastic paraplegia type 47[16]. There is also evidence of a colocalised eQTL signal for *PTPN22* (*protein tyrosine phosphatase, non-receptor type 22*) in three tissues. PTPN22 interacts with the proto-oncogene CBL, a member of the E3 ubiquitin ligase family that has been implicated in several cancers.

Chr1q24.2. rs35675808 is located downstream of the 3' UTR of *CD247 (CD247 molecule),* which encodes T-cell receptor zeta that constitutes the T-cell receptor-CD3 complex, coupling antigen recognition to several signalling transduction pathways, essential in adaptive immune response[17,18]. Pathways that have been shown to be implicated with this gene include HIV life cycle and translocation of ZAP-70 to immunological synapse (Reactome). Mutations in this gene are associated with autosomal recessive immunodeficiency 25 (IMD25 [MIM: 610163]), characterised by T-cells impaired response to alloantigens, tetanus toxoid and mitogens. Another gene, the *POU2F1* (*POU class 2 homeobox 1*), located 3kb upstream of this variant, might be biologically relevant. This gene, also known as the *OCT1*, belongs to the first identified members of the POU transcription factor family[19,20]. Members of this family contain the POU domain, a 160-amino acid region necessary for DNA binding to the octameric motif (5'-ATGCAAAT-3')[19]. POU2F1, as a transcriptional factor, is involved in cell cycle regulation and transcription of histone H2B and other cellular housekeeping genes[20,21]. It has also been suggested that the expression of histone H2B was downregulated in response to double-stranded DNA breaks via a mechanism that modulates transcriptional regulatory potential of POU2F1 by site-specific phosphorylation[22]. POU2F1 is implicated with various pathways, including the RNA Polymerase III transcription initiation, cytokine signalling in immune system, BRCA1 pathway and glucocorticoid receptor signalling (Reactome). This gene also facilitates human herpes simplex virus (HSV) infection by forming a multiprotein-DNA complex with the virion proteins, activating transcription of the viral immediate early genes[23].

Chr1q42.12. Variants at this locus are focused across the *PARP1* gene, which encodes the first protein member of the poly(ADP-ribosyl)transferases family, also termed as the ADP-ribosyltransferases with diphtheria toxin homology (ARTDs). It plays an essential role in various pathways of DNA repair and chromatin remodelling, including single- and double-strand break repair, nucleotide excision repair, stabilization of replication forks, and modulation of chromatin structure, thereby maintaining genomic integrity and stability[24]. Because the DNA double-strand breaks structurally resemble telomeres, regulators and components of DNA repair machinery have been shown to be implicated in telomere homeostasis[25]. Of note, rs1136410 (r^2=1.0 to the lead) causes a known V762A substitution in

PARP1 (poly(ADP-ribose) polymerase 1), which has been shown to reduce PARP1 activity. The allele that reduces activity is associated with shorter LTL, consistent with previous studies where knockdown of PARP1 leads to telomere shortening. PARP1 was identified as a telomeric double-stranded repeats binding factor in a proteomic study of telomeres using DNA *in situ* hybridization in conjugation with mass spectrometry[26]. In addition to the coding change there is also eQTL evidence for *PARP1* (S-PrediXcan and COLOC, online methods, Table S7) in pancreas, with the shorter LTL allele associating with reduced *PARP1* expression. Another SNP, rs907187, is highlighted in the integrated analysis of non-coding variants and is located within the 5' UTR of *PARP1*, which could mediate the effect on gene expression.

Chr2p16.2. rs754017156 is located within intron 3 of *ACYP2* (*acylphosphatase 2*) and also causes an in-frame insertion of two amino acids into *TSPYL6* (*TSPY like 6*). This gene encodes a nuclear protein, the Testis-Specific Y-Encoded-Like Protein 6, that involves in the nucleosome assembly. Biological function of this protein is largely unexplored. Studies have associated genetic polymorphisms of this gene region with increased risk of ischemic stroke[27], and breast cancer in the Han Chinese population[28]. There are no high LD SNPs, but an evidence of an eQTL in testis for *TSPYL6*.

Chr2q34. rs56810761 is located within intron 7 of *UNC80* (*unc-80 homolog, NALCN channel complex subunit, A*) gene. There are no high LD SNPs, but an evidence of an eQTL for *SNAI1P1* (*snail family zinc finger 1 pseudogene 1*) in testis in the co-localisation analysis. *SNAIP1* is a processed pseudogene of *SNAI1*, which encodes the human ortholog of a zinc finger protein of the snail family, first cloned in *Drosophila*, which was demonstrated to be essential in the formation of mesoderm during gastrulation and embryonic development[29].

Chr3q12.3. This locus consists of a 77 SNPs located predominantly across *SENP7* (*SUMO1/sentrin specific peptidase 7*) gene. The lead SNP is located 53bp upstream of *SENP7* within a proximal promoter. It is associated with a DNaseI sensitivity QTL and with *SENP7* expression in one tissue (co-localisation). Lower expression of *SENP7* associates with shorter LTL. Although it has no known role in telomere regulation, the small ubiquitin-like modifier (SUMO) functions as a post-translational modification, regulating various biological events, especially in DNA repair, chromatin organization, transcription, and RNA metabolism[30], which are essential biological events pertinent to telomere homeostasis.

Chr3q13.2. The variants in this region are all located within intron 2 of a predicted mRNA, *RP11-572M11.4* and downstream of a non-coding RNA *RP11-572M11.3* (also named *LINC02044*). There is no supporting evidence to suggest which gene is potentially influenced at this locus.

Chr3q26.2. This locus contains 47 SNPs in high LD ($r^2 < 0.8$) with the lead SNP (rs1093660). The *telomerase RNA component* (*TERC*) is the functional candidate in this locus. One SNP (rs2293607, r^2=0.81 to rs1093660) is located 63bp downstream of the *TERC* sequence, which potentially leads to altered *TERC* expression[31]. However, the lead variant, rs10936600, encodes a L241I substitution within *LRRC34* (*Leucine rich repeat containing 34*), which is predicted to be deleterious (Table S6). The CADD score (19.81) places this SNP just outside of the 1% most deleterious mutations. LRRC34 is a member of the leucine rich repeat containing protein family. Although little is known about its biological function, it has been suggested to

be implicated in the maintenance and regulation of pluripotency[32]. Knock down of LRRC34 results in reduced expression of some, but not all, pluripotency genes[32]. As genes encoding the telomerase enzyme share the same expression patterns as those of the pluripotency genes, thereby they are potentially subjected to the LRRC34-mediated transcriptional regulation. Another highly linked variant, rs10936599 (r^2=1.0) is predicted to have a functional effect in the integrated analysis of non-coding variants (Table S7). It is located on the edge of the active promoter region of *MYNN*, just inside the coding sequence. An eQTL is observed for *MYNN* in testis (shorter TL associated with higher expression), suggesting that this SNP may alter *MYNN* expression. MYNN protein is a member of the BTB/POZ and zinc finger containing family that is involved in transcriptional regulation. It has also been shown to interact with CUL3, a core component of the E3 Ubiquitin ligase complex, which functions in many cellular processes including DNA repair. LTL variants at this locus have been associated with idiopathic pulmonary fibrosis, of which telomere dysregulation is attributed to the disease aetiology[33]. Despite the obvious involvement of *TERC* in telomere length regulation, little bioinformatic evidence is available to support it to be the only likely-causal gene in this region, i.e. other candidate genes might also explain the locus association, such as *LRRC34* and *MYNN*. However, it is also possible that with *TERC* being a processed non-coding RNA, the relevant information is limited in standard datasets. There are no eQTLs for *TERC* in the GTex dataset, but a study has shown that variants in the regulatory region can affect its expression level, possibly by facilitating the maturation of *TERC* via 3' processing[31].

Chr4q13.3. The lead variant rs13137667 is located within the first intron of *MOB1B* (*MOB kinase activator 1B*). There are 49 variants in high LD, the majority of which are located intronically within *MOB1B* or *DCK* (*deoxycytidine kinase*). No high LD non-synonymous variants or co-localised eQTLs were found at this locus. MOB (Mps one binder) was originally identified as an Mps1 binding protein in yeast, regulating mitotic checkpoint and cytokinesis, and is evolutionarily conserved across all major kingdoms[34]. Human MOB1B homolog activates LATS1/2 (Large tumour suppressor 1/2) through protein-protein interaction in the Hippo signalling pathway, resulting in the inhibition of cell proliferation, apoptosis, and thus tumour suppression[35]. DCK is a key component of the deoxyribonucleoside salvage pathway and phosphorylates deoxycytidine, deoxyguanosine and deoxyadenosine to dCMP, dGMP and dAMP respectively.

Chr4q31.23. There are 65 associated variants clustered towards the 5' end of *DCLK2* (*doublecortin like kinase 2*). There is an eQTL co-localised with *DCLK2* in one tissue (Table S7). *DCLK2* encodes a protein that contains four independent functional domains: two doublecortin domains at the N-terminus, essential for microtubule binding and regulating microtubule polymerisation, a serine/threonine protein kinase domain at the C-terminus, sharing substantial homology to Ca^{2+}/calmodulin-dependent protein kinase, and a serine/proline-rich domain in between the two termini, which mediates multiple protein-protein interactions. Mouse models with single or double copies of *Dclk2* gene ablated are viable and fertile, however, a simultaneous deletion of *Dcx* gene, encoding another protein member of the doublecortin family, results in spontaneous seizures, hippocampal disorganisation and poor survival[36], phenotypically mimicking human lissencephaly, X-linked, 1 disease (LISX1 [MIM: 300067]).

Chr4q32.2. This locus contains 70 closely related (r^2>0.8) SNPs spanning *NAF1* (*nuclear assembly factor 1 ribonucleoprotein*), a gene encoding an RNA-binding protein, required for the synthesis of box H/ACA RNAs and sequential assembly with proteins to form ribonucleoprotein (RNP) complex. The box H/ACA RNPs regulates three fundamental cellular processes: protein synthesis, mRNA splicing via site-specific pseudouridylation of ribosomal RNAs and small nuclear RNAs and telomere maintenance by facilitating the maturation of *TERC* in telomerase[37]. Expression evidence was found for *NAF1* (S-PrediXcan and COLOC) and an antisense transcript *RP11-563E2.2* (COLOC, online methods, Table S7). The lead SNP, rs4691895, is a non-synonymous variant in *NAF1* (L368V) along with another high LD variant (rs4691896, r^2=1, I162V). Individually both are predicted to be benign; however, it is unclear what effects they may have in combination.

Chr5p15.33. There are two independently associated SNPs at this locus, neither of which have any high LD variants. Both SNPs are located within intron 2 of *TERT*, but little functional evidence was found to support their involvements in regulating *TERT* levels, which might be due to the transcriptional repression of *TERT* in most somatic tissues.

Chr5q14.1. The lead variant, rs62365174, is located in intron 4 of *TENT2* (*terminal nucleotidyltransferase 2*, previously named *PAPD4 and GLD2*). There are 137 SNPs in high LD (r^2<0.8), which fall across the region of *TENT2* and include upstream, intronic and 3' UTR variants. There is strong evidence that these variants can affect the expression of *TENT2*, with eQTLs co-localised in 9 tissues, exhibiting consistent positive correlations, i.e. reduced expression associates with decreased LTL. TENT2 functions as the cytoplasmic poly(A) RNA polymerase that adds successive AMP monomers to the 3'-end of specific RNAs, forming a poly(A) tail, exhibiting strict substrate specificity, that, different from the canonical nuclear poly(A) RNA polymerase, only functions on cytoplasmic RNAs[38]. Previous studies have suggested its role in the polyadenylation and stability of p53 mRNA[39] and several miRNAs[40].

Chr5q31.2. The associated variant, rs112347796, has no further variants in high LD (r^2>0.8). It is located within intron 1 of *UBE2D2* (*ubiquitin conjugating enzyme E2 D2*), which is involved in the DNA damage repair[41]. There is no evidence to suggest the potential function of this variant.

Chr6p22.2. This locus contains 10 SNPs in high LD (r^2>0.8) with the lead SNP, all located around *CARMIL1* (*capping protein regulator and myosin 1 linker 1*, previously named *LRR16A*). One SNP, rs913455, causes a synonymous change within exon 3 and has scored to have possible regulatory function (Table S8), which may be driven in part by its high conservation and location within the coding region. There is no supporting literature evidence to identify which gene(s) may be influenced at this locus.

Chr6p21.33. There are 11 SNPs in high LD (r^2>0.8) with the lead SNP, which are located across the major histocompatibility complex (MHC) class III region. MHC is a highly polymorphic and gene-dense region with complex linkage disequilibrium structure, and thus characterisation of potential causal genes within this region is difficult. A number of genes can potentially serve as causal gene candidates, including *PRRC2A*, *CSNK2B* and *BAG6*. There is evidence that the expression of both *BAG6* and *CSNK2B* (S-PrediXcan and COLOC, Table S7) is affected. The lead variant is located upstream of *PRRC2A*, which was previously known as the *BAT2* (*HLA-B*

associated transcript 2) gene, encoding a large protein (2157 amino acids). PRRC2A has been shown to be involved in the pre-mRNA editing, as spliceosome and splicing regulators were found to be able to bind to the PRRC2A in protein-protein interaction assays, including the heterogeneous nuclear RNPs and the cleavage and polyadenylation specific factor 1[42]. As maturation of the telomerase RNA subunit involves a spliceosome-mediated single cleavage reaction[43], PRRC2A may regulate telomere length via involvement in the biogenesis of *TERC*. Of note, another variant, rs805299 (r^2=1), located within intron 1 of *BAG6* (*BCL2 associated athanogene 6*), shows a high probability for promoter activity and is predicted to have regulatory function in the integrated analysis of non-coding variants (Table S8). *BAG6* was part of a cluster of genes that encode a multifunctional protein, involved in various pathways, including intracellular protein quality controls by promoting proteasomal degradation of misfolded and mislocalised proteins, and DNA damage-induced apoptosis. Another variant, rs5872 (r^2=1), is located within the 3'UTR of *CSNK2B* (*casein kinase 2 beta*). CSNK2B is a subunit of CSNK2 that is involved in multiple pathways but of note has been shown to interact with TRF1. CSNK2-mediated phosphorylation of TRF1 is required for the binding of TRF1 to telomeres, which has been proposed to be essential for telomere length homeostasis[44].

Chr7q31.33. The associated variants cover the *POT1* (*protection of telomeres 1*) gene, which encodes the most conserved protein component of the shelterin complex among all eukaryotes[45]. It is tethered to the TERF1 and TERF2 homodimers via a TIN2-mediated linkage, and specifically bound to the single-stranded telomeric repeats, protecting it from nucleolytic degradation[46]. Moreover, POT1 controls the sequence precision at the 5' ends, which are identical among nearly all human chromosomes, and regulates telomere length by restricting telomerase binding[47]. Rare nonsense mutations within this gene, which blocked physical interactions of POT1 with telomeric single-stranded repeats and other components of the shelterin protein complex, were identified by whole-exome sequencing in families with strong histories of chronic lymphocytic leukaemia[48]. The integrated analysis of non-coding variants highlights rs2239532 (r^2=0.85), located within the 5'UTR of *GPR37* (*G protein-coupled receptor 37*), as having regulatory function (Table S8). Although no direct eQTL evidence is available to support POT1, there is evidence to link the expression of an uncharacterised *POT1-AS* transcript (*RP11-3B12.1*) to LTL via co-localisation in two tissues (Table S8).

Chr8p23.2. This region contains 52 SNPs in high LD (r^2<0.8) and is located within 3 introns towards the 3' end of *CSMD1* (*CUB and Sushi multiple domains 1*) gene. *CSMD1* was potentially associated with a rare neurological disease, the benign adult familial myoclonic epilepsy[49]. It may also act as a suppressor of squamous cell carcinomas, yet unequivocal evidence is lacking[50,51]. The gene-knockout mouse was used as a schizophrenia human disease model, exhibiting increased levels of exploratory activity, behavioural despair anxiety-related response, and decreased startle reflex (MGI: 3528558). However, no direct supporting evidence is available to suggest CSMD1 or other genes as causal gene candidates in this region.

Chr8q22.2. Four SNPs are located upstream of *COX6C* (*cytochrome c oxidase subunit 6C*). COX6C is a subunit of complex IV that catalyses the final step of the mitochondrial respiratory chain[52]. No functional data is available to pinpoint causal genes for this locus.

Chr10p15.1. The 6 associated variants (in LD, r^2>0.8) at this locus are clustered within the first intron of *ASB13* (*ankyrin repeat and SOCS box containing 13*), a member of the suppressor of cytokine signalling box protein superfamily. Members of this protein family can also be components of E3 ubiquitin ligase complexes[53]. No causal gene candidates can be prioritised for this locus.

Chr10q24.33. This region contains *STN1* (*STN1, CST complex subunit*, also termed *OBFC1* in humans), a component of the telomere binding CST complex. There is strong evidence that the variants affect *STN1*(*OBFC1*) expressions across multiple tissues (S-PrediXcan and COLOC, Table S7). The CST complex regulates telomere maintenance by mediating the access to telomeres for telomerase and DNA polymerase α[54].

Chr11q21. The lead variant, rs117037102, is located within intron 5 of *CEP295* (*centrosomal protein 295*, also termed *KIAA1731*). There is a potentially damaging protein coding variant (rs117405490, r^2=1), which results in a P to A substitution at position 783 of CEP295. CEP295 is a centriole-enriched microtubule-binding protein, highly conserved across species and involved in centriole biogenesis, essential for cell cycle regulation and mitotic progression[55].

Chr11q22.3. The associated variants fall across a ~321kb region which includes several genes, including *ATM* (*ATM serine/threonine kinase*), encoding a protein kinase that phosphorylates many checkpoint-determining and regulatory proteins, such as p53, Chk2 and BRCA1, and thus playing an essential role in cell cycle control and DNA-damage-activated signalling pathways[56]. ATM is responsible for the human genetic disorder ataxia telangiectasia (AT [MIM: 208900]), manifested with genome instability, cerebellar and thymic degeneration, immunodeficiency, premature ageing, sensitivity to ionizing radiation and predisposition to cancer[57]. There are eQTLs supporting *ATM* and another gene, *ACAT1* (*acetyl-CoA acetyltransferase 1*), within the region. ACAT1 is a mitochondrial protein, expression levels of which have been linked to some cancers[58]. Defects in this gene are associated with 3-ketothiolase deficiency, an inborn error of isoleucine catabolism[59].

Chr12p13.1. The lead variant and 2 in high LD (r^2<0.8) are located upstream of *ATF7IP* (*activating transcription factor 7 interacting protein*), also named *MCAF1*, actively involved in histone modification, chromatin organisation, and Sp1-dependent maintenance of telomerase activity in cancer cells[60]. It was previously shown to regulate expression of both *TERT* and *TERC* and consequently telomerase activity[61].

Chr12q13.13. There are 7 variants in high LD (r^2 <0.8), located within a 3kb region upstream of *SMUG1* (*single-strand-selective monofunctional uracil-DNA glycosylase 1*), a gene involved in base-excision repair. Although there is no bioinformatic evidence to show that these variants affect SMUG1 expression levels, previous functional studies have suggested that SMUG1 might influence telomere length by interacting with the telomerase component Dyskerin (DKC1) with which it controls rRNA processing[62].

Chr14q24.2. The lead variant is a non-synonymous (W22C) variant in *DCAF4* (*DDB1 and CUL4 associated factor 4*). Another variant in high LD (rs3815460, r^2=1) also causes a protein coding change (S345C). Both variants are predicted to be damaging individually. DCAF4 interacts with the Cul4-Ddb1 E3 ubiquitin ligase macromolecular complex, which regulates processes

including DNA repair and cellular proliferation[63]. *DDB* (*DNA damage binding protein*) is highly expressed in multipotent hematopoietic progenitors, conditional ablation of which in hematopoietic stem and progenitor cells led to a complete loss of pluripotency and self-renewal of progenitors and stem cells, suggesting its role in cell differentiation, apoptosis and death[64]. An intronic G-to-A variant (rs2535913) has been associated with shorter LTL[65]. A further SNP, rs2286838 (r^2=0.9) causes a coding change in *ZFYVE1* (*zinc finger FYVE-type containing 1*, S408R), which also has a predicted damaging effect. This protein, also known as the *double FYVE-containing protein 1* (*DFCP1*), contains two zinc-binding FYVE domains in tandem, which has been shown to be localised on endoplasmic reticulum and Golgi apparatus via binding to phosphatidylinositol 3-phosphate containing membranes, essential for the regulation of autophagy[66].

Chr14q24.3. The lead variant, rs59192843, is located within intron 6 of *BBOF1* (*basal body orientation factor 1*, also termed as *CCDC176*). There are no coding variants or eQTLs associated with the lead variant. Two variants in high LD (r^2<0.8), rs73301475 and rs17094157 scored highly in the integrated analysis of non-coding variants (Table S8). These are located within an enhancer of *ENTPD5* (*ectonucleoside triphosphate diphosphohydrolase 5*) and the 3' UTR of *COQ6* (*coenzyme Q6, monooxygenase*), respectively. ENTPD5 hydrolyses UDP to UMP to promote protein N-glycosylation and folding. It has been shown that ENTPD5 was upregulated in cell lines and primary human tumour samples with active AKT, promoting cell growth and survival[67]. AKT activation also contributes to the elevation of aerobic glycolysis seen in tumour cells, known as the Warburg effect. Of note, ENTPD5 was also involved in stimulating glycolysis by providing substrates for cytidine monophosphate kinase-1 that converts UMP to UDP using a phosphate molecule generated during the ATP hydrolysis cycle[68]. COQ6 is an evolutionarily conserved monooxygenase, belonging to the ubiH/COQ6 family, which is required for the biosynthesis of coenzyme Q10 (or ubiquinone), an essential component of the mitochondrial electron transport chain and one of the most potent lipophilic antioxidants implicated in the protection of cell damage by reactive oxygen species. Gene-ablated mouse model showed abnormal embryo size and growth retardation (MGI: 5548683). Mutations in this gene are associated with autosomal recessive coenzyme Q10 deficiency-6, which manifests as nephrotic syndrome with sensorineural deafness[69].

Chr14q32.11. In this locus the variants are focused across *CALM1* (*calmodulin 1*). There is an eQTL co-localised with *CALM1* expression in testis. Two SNPs (rs12885713 and rs2300496) are within the *CALM1* promoter/enhancer region and predicted to have regulatory function. *CALM1* encodes a member of the EF-hand calcium-binding protein family, regulating a number of protein kinases and phosphatases, among which CP110, by interacting with CALM1 and centrin, regulates centrosome function and cytokinesis[70].

Chr14q32.33. The lead SNP, rs117536281, is located upstream of *CDCA4* (*cell division cycle associated 4*). *CDCA4* encodes a member of the E2F family of transcription factors, regulating spindle organization, cytokinesis and cell proliferation, which may be also involved in differentiation of hematopoietic stem cells and progenitor cell lineage[71]. There are no coding variants or eQTL data for this locus.

Chr15q14. This locus consists of two associated SNPs, rs9972513 and rs12324579, which are located in an intergenic region upstream of both *c15orf53* and *RASGRP1* (*RAS guanyl releasing*

protein 1). There are no coding variants or eQTL data for this locus. *C15orf53* is a protein coding gene with uncharacterised functions, with disputable evidence suggesting its implication with schizophrenia and bipolar disorder[72]. *RASGRP1* encodes a protein that functions as a calcium- and diacylglycerol (DAG)-regulated nucleotide exchange factor specifically activating Ras through the exchange of bound GDP for GTP. RASGRP1 contains a pair of calcium-binding EF hands and a DAG-binding domain[73]. The RASGRP1-mediated Ras activation regulates T cell proliferation, development and homeostasis[74].

Chr15q21.2. There are 17 SNPs clustered around the 5' end of *ATP8B4* (*ATPase phospholipid transporting 8B4 (putative)*). There are no coding variants or eQTL data for this locus. *ATP8B4* encodes a member of the cation transport ATPase (P-type) family and type IV subfamily, which consists of a P4-ATPase flippase complex that catalyses the hydrolysis of ATP coupled to phospholipid translocation across various membranes, playing a role in vesicle biosynthesis and lipid signalling transduction[75,76]. Deleterious rare variants within this gene have been associated with systemic sclerosis, for which the principal cause of death was pulmonary diseases, including interstitial lung disease and pulmonary arterial hypertension[77]. An intronic common variant at the distal promoter region of this gene has been reported to be associated with Alzheimer's Disease[78].

Chr15q21.3. This single variant, rs117610974 is located in an intergenic region, ~220kb downstream of the closest gene, *UNC13C* (*unc-13 homolog C*), which might be implicated with vesicle formation during exocytosis, with potential capabilities of diacylglycerol and calcium binding[79]. However, there is no evidence to suggest what role this lead variant may have.

Chr15q22.31. The lead variant, rs55710439, is located within intron 6 of *ANKDD1A* (*ankyrin repeat and death domain containing 1A*). There is an eQTL for this gene co-localised in one tissue. Little is known about the ANKDD1A protein, except that it contains an ankyrin repeat domain and a death domain, both of which function in the protein-protein interaction. A closely-related SNP (in LD, $r^2<0.8$), rs57438358, predicted to have potential functional effects, is located within the 3'UTR of *SPG21* (*SPG21, maspardin*), a gene which is mutated in mast syndrome.

Chr16p13.3. This is a single variant, rs11640926, located within intron 5 on *CACNA1H*. There is no supporting evidence to suggest the effects of this variant. *CACNA1H* encodes a protein component of the voltage-dependent calcium channel complex, a T-type calcium channel that belongs to the "low-voltage activated" group, which plays an essential role in both central neurons and cardiac nodal cells and supports calcium signalling in secretory cells and vascular smooth muscle[80,81]. It is associated with a form of familial hyperaldosteronism, clinically characterised by hypertension, elevated aldosterone levels and abnormal adrenal steroid production[82]; and another genetic rare disease, the Childhood Absence Epilepsy 6[83].

Chr16q22.1. The most significantly associated variants in this region are located within and around *TERF2* (*telomeric repeat binding factor 2*), a component of the shelterin complex. TERF2 protein directly and specifically binds to the telomeric double-stranded repeats, and by interacting with other telomeric factors forming a T-loop configuration that protects chromosome ends from disruptive end-to-end joining and ligation to exogenous DNA. Mutant

forms of this gene induced DNA fusion, such as formation of anaphase bridges and lagging or ring-like chromosomes[84,85].

There is evidence that the variants affect expression of several genes in this region, with the strongest evidence for *TERF2* (S-PrediXcan and COLOC, Table S7). Longer LTL is associated with reduced expression of *TERF2*, consistent with TERF2 being a negative regulator of telomere length[86]. One variant predicted to have a functional effect, rs9939705, is located within an enhancer region upstream of *TERF2*. There is also evidence to suggest that expression of two other genes (*COG8*, and *PDF*) are also affected by the associated variants.

Chr16q23.1. Variants at this locus show co-localisation with eQTLs for *RFWD3* (*ring finger and WD repeat domain 3*) in multiple tissues. RFWD3 is a ubiquitin ligase that interacts with and ubiquitinates replication protein A (RPA), which has been shown to be essential for DNA replication and repair. Upon replication stress, RPA was recruited to stalled replication folks and ubiquitinated by the RFWD3, an essential process for recovery and homologous recombination-mediated DNA repair[87]. RFWD3 also ubiquitinates and stabilises p53/TP53 in response to DNA damage, thereby regulating the cell cycle checkpoint[88]. This gene was also clinically attributable to the Fanconi anaemia (FA [MIM: 227650]), an autosomal recessive inheritance disease manifested with chromosomal instability, bone marrow failure, dermal pigmentary changes and predisposition to malignancies.

Chr16q23.3. The association signal at this locus is across *MPHOSPH6* (*M-phase phosphoprotein 6*). There is strong eQTL evidence (S-PrediXcan and COLOC) in multiple tissues to support the associated variants influencing MPHOSPH6 expression. MPHOSPH6 is a component of the RNA exosome, a protein complex required for the degradation of RNA molecules and is required for the 3’ processing of the 5.8S rRNA[89]. There is also evidence that MPHOSPH6 interacts with PARN (poly(A)-specific ribonuclease)[90], an important regulator of mRNA catabolism which is also required for the formation of mature *TERC* RNA[91].

Chr17q25.3. The lead variant (rs144204502) is situated within the 5’ UTR of *TK* (*thymidine kinase 1*), with evidence of regulatory functions (Table S8). There are co-localised eQTLs for *TK1* in three tissues. *TK1* encodes a cytosolic enzyme that catalyses the conversion of thymidine to dTMP, which is the first step of the salvage pathway of dTTP biosynthesis, essential for DNA replication. There are two forms of the TK enzyme, besides the TK1, TK2 catalyses the same reaction but in the mitochondria. The activity of TK1 is delicately regulated by a configurational transition, changing from dimer to tetramer upon increases in ATP and enzyme concentrations, with a consequently accompanied upregulation of catalytic efficiency[92]. This regulatory fine-tuning of TK1 activity ensured a balanced pool of nucleic acid precursors. High TK1 expression was detected in numerous types of cancers, including gastrointestinal adenocarcinomas and oesophageal and uterine squamous cell carcinomas[93].

Chr18p11.32. All variants within the locus are located within the *TYMS* (*thymidylate synthetase*) gene, either within the intronic or the 3’UTR regions. There is an eQTL for *TYMS* co-localised in one tissue. TYMS is involved in the *de novo* biosynthesis of dTMP, catalysing the methylation of dUMP to dTMP using a serine-derived one-carbon donor, the 5,10-methyleneTHF[94]. *TYMS* has been targeted for cancer chemotherapeutics, as high expression of which has been detected in various types of cancers, including gastrointestinal adenocarcinomas and squamous cell uterine carcinomas[93].

Chr19p13.3. The lead variant is located within intron 5 of *NMRK2* (*Nicotinamide Riboside kinase 2*), with 6 SNPs in high LD (r^2<0.8) located around this gene. NMRK2 enzyme catalyses the phosphorylation of nicotinamide riboside (NR) and nicotinic acid riboside (NaR) to form nicotinamide mononucleotide (NMN) and nicotinic acid mononucleotide (NaMN), the vitamin precursors of NAD^+, which is required for the function of Sirtuins, a key player in lifespan extension and energy metabolism[95]. It has been demonstrated that increased NAD^+ biosynthesis elevated the Sirtuin 2 function, which improved the subtelomeric gene silencing effects and elongated replicative lifespan in eukaryotic cell models[95]. One further variant in high LD, located upstream of *DAPK3* (*death associated protein kinase 3*), is a regulator of apoptosis. There is no functional data supporting any gene candidates at this locus.

Chr19p12. The lead variant is intergenic, located between *ZNF257* and *ZNF208*, with closer proximity to the former. There is eQTL evidence for both *ZNF257* and *ZNF265*, yet stronger for the *ZNF257* (Table S7). *ZNF257* encodes a member of a zinc finger protein family, the Krüppel-like zinc finger subfamily, signified by a consensus sequence of TGEKPYX (X denotes any amino acids) between concatenated zinc finger motifs[96]. The proteins have the KRAB domain at their amino terminus, which determines the specificity of binding to DNA and other transcriptional co-regulators.

Chr19q13.2. The single associated variant, rs11665818, is located within an intergenic region, downstream of *INFL2* (*interferon lambda 2*, also termed *IL28a*) and within a cytokine gene cluster that consists of three closely related *INFL* genes. *INFL2* encodes a protein with antiviral activities, predominantly in the epithelial tissues[97]. There is no supporting functional evidence at this locus.

Chr20p12.3a. The lead and one variant in high LD (r^2<0.8) are located upstream of *PROKR2* (*Prokineticin receptor 2*), a G protein-coupled receptor for the prokineticin 2, which is a secreted protein expressed in gut and brain, and has been shown to oscillate on a circadian basis[98]. Homozygous gene-knockout mice showed impaired circadian behaviour and thermoregulation (MGI:2181363). Mutations in this gene led to gonadotropin-releasing hormone deficiency and hypogonadism[99]. There are no coding variants or eQTLs associated with this locus.

Chr20p12.3b All variants of this locus are located within an intergenic region, with the closest gene being LINC01706 (long intergenic non-coding RNA 1706), an uncharacterised non-coding transcript.

Chr20q11.23. The association signal spans two genes *MROH8* (*maestro heat like repeat family member 8*) and *RBL1* (*RB transcriptional corepressor like 1*). There is eQTL evidence to support changes in both *RBL1* and *SAMHD1* (*SAM and HD domain containing deoxynucleoside triphosphate triphosphohydrolase 1*) expression. RBL1 functions as a transcriptional repressor for E2F binding sites-containing genes[100], which shares similarity in amino acid sequence and biochemical features to the *retinoblastoma 1* (*RB1*) gene product that functions as a tumour suppressor implicated in cell cycle regulation. *SAMHD1* encodes a dNTP triphosphohydrolase (dNTPase) that converts deoxynucleoside triphosphates (dNTPs) to deoxynucleosides. The gene expression was regulated during cell cycle to maintain a homeostatic pool of dNTP,

required for DNA replication [101]. Studies have suggested an antiretroviral role of SAMHD1 in dendritic and myeloid cells by depleting the intracellular pool of dNTPs[102,103].

Chr20q13.33. There are four independent signals within this locus, which harbours several genes, including the DNA helicase *RTEL1* (*regulator of telomere elongation helicase 1*). There are non-synonymous coding variants in *RTEL1* and *ZBTB46* (*zinc finger and BTB domain containing 46*) although neither are predicted to be deleterious. There are eQTLs for *RTEL1*, *STMN3*(*stathmin 3*) and *TNFRSF6B* (*TNF receptor superfamily member 6b*, also termed *decoy receptor 3*). *RTEL1* encodes an ATP-dependent DNA helicase that functions in the regulation of telomeres, DNA repair and genomic integrity. RTEL1 facilitates access of telomerase to the 3' ends of telomeres by transiently dismantling the T-loop configuration, a lariat-like structure that protects telomeres from degradation and deleterious DNA damage response[104]. Mutations of this gene led to Hoyeraal Hreidarsson syndrome, a clinically severe form of dyskeratosis congenita, of which half of the inherited families carry germline mutations of telomere-related genes[105]. Loss-of-function missense variants of this gene was found to be associated with idiopathic pulmonary fibrosis and shortened telomere lengths[106]. *STMN3* gene encodes a member of the stathmin protein family, which shows microtubule-destabilizing activity and is known to be involved in the development of central nervous system and glioma pathology[107]. *TNFRSF6B* is a regulator of apoptosis and has been linked to angiogenesis[108–110]. *ZBTB46* gene encodes a member of a large BTB zinc-finger protein family, characterised by a DNA binding motif that consists of a tandem array of C2H2 krüppel-like zinc fingers at the carboxyl terminus, with each finger containing a consensus sequence of ~30 amino acids and an embedded zinc ion[111]. In contrast, the BTB domains at the amino termini are more divergent across the family, mainly contributing to the hetero- or homo-dimerization. The BTB domain determines DNA binding specificity and recruitment of co-regulators to form higher chromosomal structures[111]. ZBTB46 has been shown to function as a transcriptional repressor involved in prostate cancer malignancy and cell cycle regulation[112]. Recently, studies have identified another member of the BTB zinc-finger protein family, ZBTB48, also termed as the telomeric zinc finger–associated protein, to be specifically associated with telomeres via the zinc finger domain. Further investigation demonstrated that it was preferentially bound to longer telomeres where protein components of the shelterin complex are rather sparse[113]. Experimental studies suggested that the ZBTB48 might compete with the TERF2 for binding to the telomeric DNA repeats, thereby setting an upper limit of the telomere length, which can further influence lifespan and cancer susceptibility[113,114]. Because the zinc finger domain is conserved among all members of the family, we speculated that the ZBTB46 was also capable of binding to the telomeric DNA, regulating telomere homeostasis via similar mechanisms. However, further experiments are required to validate this hypothesis.

Chr21q22.3. The lead variant is a loss-of-stop mutation in *KRTAP10-4* (*keratin associated protein 10-4*), which was located within a cluster of related genes, encoding proteins that form disulfide bonds between cysteine residues in hair keratins. A genome-wide siRNA-based screen implicated this gene with the homologous recombination DNA double-strand break repair[115]. Although transcripts lacking stop codons would be targeted for degradation, there is no eQTL evidence to suggest loss of expression with this allele, possibly due to poor detection of this transcript in GTex (Median transcripts per million=0). There is one variant in

high LD, located within intron 2 of *TSPEAR* (*thrombospondin type laminin G domain and EAR repeats*), a regulator of the NOTCH signalling.

Chr22q13.31. This is a single variant located within intron 1 of *KIA1644* (Also termed *SHISAL1*). There is no supporting functional data for gene prioritisation at this locus.

Supplemental Acknowledgements

ENGAGE cohorts

BHF-FHS. The British Heart Foundation Family Heart Study (BHF-FHS) was funded by the British Heart Foundation (BHF). Genotyping of the BHF-FHS study was undertaken as part of the WTCCC and funded by the Wellcome Trust.

EGCUT. EGCUT received financing from Horizon2020 programs (ePerMed), targeted financing from Estonian Government IUT 20-60 Estonian Research Roadmap through Estonian Ministry of Education and Research, Centre of Excellence in Genomics and Translational Medicine (GenTransMed). The work of Krista Fischer and Reedik Mägi has also been supported by Estonian Science Foundation grant EstSF ETF9353. We acknowledge EGCUT technical personnel, especially Mr V. Soo and S. Smit. Data analyzes were carried out in part in the High Performance Computing Centre of University of Tartu.

ERF. The ERF study as a part of EUROSPAN (European Special Populations Research Network) was supported by European Commission FP6 STRP grant number 018947 (LSHG-CT-2006-01947) and also received funding from the European Community's Seventh Framework Programme (FP7/2007-2013)/grant agreement HEALTH-F4-2007-201413 by the European Commission under the programme "Quality of Life and Management of the Living Resources" of 5th Framework Programme (no. QLG2-CT-2002-01254). High-throughput analysis of the ERF data was supported by joint grant from Netherlands Organization for Scientific Research and the Russian Foundation for Basic Research (NWO-RFBR 047.017.043). We are grateful to all study participants and their relatives, general practitioners and neurologists for their contributions and to P. Veraart for her help in genealogy, J. Vergeer for the supervision of the laboratory work and P. Snijders for his help in data collection.

Finnish Twin Cohort/Nicotine Addiction Genetics-Finland study. This work was supported for data collection by Academy of Finland grants (J Kaprio) and a NIH grant DA12854 (PAFM). Genome-wide genotyping in the Finnish sample was funded by Global Research Award for Nicotine Dependence / Pfizer Inc. (JK), and Wellcome Trust Sanger Institute, UK. This work was further supported by the Academy of Finland Centre of Excellence in Complex Disease Genetics (grant numbers: 213506, 129680, J Kaprio) and the European Community's Seventh Framework Programme ENGAGE Consortium, (grant agreement HEALTH-F4-2007-201413). We thank Sirli Raud and Anne Vikman for help in TL measurement.

Finrisk. The FINRISK 2007/ DILGOM-study was supported by the Academy of Finland, grant # 118065. S.M. was supported by grants #136895 and #141005 and V.S. by grants #139635 and 129494 from the Academy of Finland.

GRAPHIC. The GRAPHIC study was funded by the BHF.

HBCS. The study was supported by grants to J.G.E. from the Academy of Finland (JGE: 135072, 129255, 126775), Finska Läkaresällskapet, Samfundet Folkhälsan, Juho Vainio Foundation, Päivikki and Sakari Sohlberg Foundation, Signe and Ane Gyllenberg Foundation, and Yrjö Jahnsson Foundation.

KORA. The KORA Augsburg studies were financed by the Helmholtz Zentrum München, German Research Centre for Environmental Health, Neuherberg, Germany and supported by grants from the German Federal Ministry of Education and Research (BMBF). Part of this work was financed by the German National Genome Research Network (NGFN). Our research was supported within the Munich Centre of Health Sciences (MC Health) as part of LMUinnovativ.

LLS The Leiden Longevity Study has received funding from the European Union's Seventh Framework Programme (FP7/2007-2011) under grant agreement n° 259679. This study was supported by a grant from the Innovation-Oriented Research Program on Genomics (SenterNovem IGE05007), the Centre for Medical Systems Biology, and the Netherlands Consortium for Healthy Ageing (grant 050-060-810), all in the framework of the Netherlands Genomics Initiative, Netherlands Organization for Scientific Research (NWO).

NESDA: Funding was obtained from the Netherlands Organization for Scientific Research (Geestkracht program grant 10-000-1002); the Centre for Medical Systems Biology (CSMB, NWO Genomics), Biobanking and Biomolecular Resources Research Infrastructure (BBMRI-NL), VU University's Institutes for Health and Care Research (EMGO+) and Neuroscience Campus Amsterdam, University Medical Centre Groningen, Leiden University Medical Centre, National Institutes of Health (NIH, R01D0042157-01A, MH081802, Grand Opportunity grants 1RC2 MH089951 and 1RC2 MH089995). Part of the genotyping and analyses were funded by the Genetic Association Information Network (GAIN) of the Foundation for the National Institutes of Health. Computing was supported by BiG Grid, the Dutch e-Science Grid, which is financially supported by NWO.

NFBC Northern Finland Birth Cohort 1966 (NFBC1966): NFBC1966 received financial support from the Academy of Finland (project grants 104781, 120315, 129269, 1114194,

139900/24300796, Centre of Excellence in Complex Disease Genetics and SALVE), University Hospital Oulu, Biocentre, University of Oulu, Finland (75617), the European Commission (EURO-BLCS, Framework 5 award QLG1-CT-2000-01643), NHLBI grant 5R01HL087679-02 through the STAMPEED program (1RL1MH083268-01), NIH/NIMH (5R01MH63706:02), ENGAGE project and grant agreement HEALTH-F4-2007-201413, the Medical Research Council, UK (G0500539, G0600705, G0600331, PrevMetSyn/SALVE, PS0476) and the Wellcome Trust (project grant GR069224, WT089549), UK.Replication genotyping was supported in part by MRC grant G0601261, Wellcome Trust grants 085301, 090532 and 083270, and Diabetes UK grants RD08/0003704 and BDA 08/0003775.The DNA extractions, sample quality controls, biobank up-keeping and aliquotting was performed in the National Public Health Institute, Biomedicum Helsinki, Finland and supported financially by the Academy of Finland and Biocentrum Helsinki. We thank Professor (emerita) Paula Rantakallio (launch of NFBC1966 and 1986), and Ms Outi Tornwall and Ms Minttu Jussila (DNA biobanking). The authors would like to acknowledge the contribution of the late Academian of Science Leena Peltonen. JLB was supported by a Wellcome Trust fellowship grant (WT088431MA).

<u>NTR.</u> We thank the Netherlands Organization for Scientific Research (NWO: MagW/ZonMW): Genotype/phenotype database for behaviour genetic and genetic epidemiological studies (NWO 911-09-032); Spinozapremie (SPI 56-464-14192); NWO-Groot 480-15-001/674: Netherlands Twin Registry Repository; BBMRI –NL: Biobanking and Biomolecular Resources Research Infrastructure (184.021.007 and 184.033.111). Amsterdam Public Health (APH) (Elucidating factors influencing the genetic background estimation in family members). European Science Foundation (ESF): Genomewide analyses of European twin and population cohorts (EU/QLRT-2001-01254); European Community's Seventh Framework Program (FP7/2007-2013): ENGAGE (HEALTH-F4-2007-201413); the European Science Council ERC- 230374; Rutgers University Cell and DNA Repository cooperative agreement (NIMH U24 MH068457-06); Collaborative study of the genetics of DZ twinning (NIH R01D0042157-01A); the Genetic Association Information Network, the Avera Institute for Human Genetics, Sioux Falls, South Dakota (USA), and the National Institutes of Mental Health (NIMH): GODOT: Integration of Genomics & Transcriptomics in Normal Twins & Major Depression (1RC2 MH089951-01) and DETECT: Developmental TrajECtories in Twins (1RC2MH089995-01). DIB acknowledges KNAW Academy Professor Award (PAH/6635).

<u>QIMR.</u> We thank Marlene Grace, Ann Eldridge, and Kerrie McAloney for sample collection; Anjali Henders, Megan Campbell, Lisa Bardsley, Lisa Bowdler, Steven Crooks, and staff of the Molecular Epidemiology Laboratory for sample processing and preparation; Harry Beeby, David Smyth, and Daniel Park for IT/database support; Scott Gordon for his substantial efforts involving the QC and preparation of the GWA data; and the twins and their families for their participation. We acknowledge support from the Australian Research Council (A7960034, A79906588, A79801419, DP0212016, and DP0343921). Telomere length

assessment was co-funded by the European Community's Seventh Framework Programme (FP7/2007-2013), ENGAGE project, grant agreement HEALTH-F4-2007-201413 and National Health and Medical Research Council (NHMRC)-European Union Collaborative Research Grant 496739. GWM was supported by an NHMRC Fellowship (619667), DRN (FT0991022) and SEM (FT110100548) were supported by an ARC Future Fellowship. Genotyping was funded by the NHMRC (Medical Bioinformatics Genomics Proteomics Program, 389891). Genotype imputation was carried out on the Genetic Cluster Computer (http://www.geneticcluster.org), which is financially supported by the Netherlands Scientific Organization (NWO 480-05-003).

TWINGENE. The Ministry for Higher Education, the Swedish Research Council (M-2005-1112), GenomEUtwin (EU/QLRT-2001-01254; QLG2-CT-2002-01254), NIH DK U01-066134, The Swedish Foundation for Strategic Research (SSF) Heart and Lung foundation no. 20070481

TwinsUK. TwinsUK is funded by the Wellcome Trust, Medical Research Council, European Union, the National Institute for Health Research - funded BioResource, Clinical Research Facility and Biomedical Research Centre based at Guy's and St Thomas' NHS Foundation Trust in partnership with King's College London.

UKBS: Recruitment of the United Kingdom Blood Service donors was funded by the Wellcome Trust as part of the WTCCC.

EPIC

EPIC-InterAct: We thank all EPIC participants and staff and the InterAct Consortium members for their contributions to the study. We thank staff from the technical, field epidemiology and data teams of the Medical Research Council Epidemiology Unit in Cambridge, UK, for carrying out sample preparation, DNA provision and quality control, genotyping and data handling work.

EPIC-CVD. this study was supported by core funding from the UK Medical Research Council (MR/L003120/1), the British Heart Foundation (RG/13/13/30194; RG/18/13/33946), the European Commission Framework Programme 7 (HEALTH-F2-2012-279233), and the National Institute for Health Research [Cambridge Biomedical Research Centre at the Cambridge University Hospitals NHS Foundation Trust]. JD is funded by the National Institute for Health Research [Senior Investigator Award].

The funding bodies had no role in the design or conduct of the study; collection, management, analysis, or interpretation of the data; preparation, review, or approval of the manuscript; or the decision to submit the manuscript for publication.

Where authors are identified as personnel of the International Agency for Research on Cancer / World Health Organization, NHS England, National Institute for Health Research or the Department of Health and Social Care, the authors alone are responsible for the views expressed in this article and they do not necessarily represent the decisions, policy or views of the corresponding organisations.

Supplementary References

1. Codd, V. *et al.* Identification of seven loci affecting mean telomere length and their association with disease. *Nat. Genet.* **45**, 422 (2013).
2. Kristiansson, K. *et al.* Genome-wide screen for metabolic syndrome susceptibility loci reveals strong lipid gene contribution but no evidence for common genetic basis for clustering of metabolic syndrome traits. *Circ. Cardiovasc. Genet.* **5**, 242–249 (2012).
3. Penninx, B. W. J. H. *et al.* The Netherlands Study of Depression and Anxiety (NESDA): rationale, objectives and methods. *Int. J. Methods Psychiatr. Res.* **17**, 121–140 (2008).
4. Ikram, M. A. *et al.* The Rotterdam Study: 2018 update on objectives, design and main results. *Eur. J. Epidemiol.* **32**, 807–850 (2017).
5. Langenberg, C. *et al.* Design and cohort description of the InterAct Project: An examination of the interaction of genetic and lifestyle factors on the incidence of type 2 diabetes in the EPIC Study. *Diabetologia* **54**, 2272–2282 (2011).
6. Langenberg, C. *et al.* Gene-Lifestyle Interaction and Type 2 Diabetes: The EPIC InterAct Case-Cohort Study. *PLoS Med.* **11**, (2014).
7. Danesh, J. *et al.* EPIC-Heart: The cardiovascular component of a prospective study of nutritional, lifestyle and biological factors in 520,000 middle-aged participants from 10 European countries. *Eur. J. Epidemiol.* **22**, 129–141 (2007).
8. Crowe, F. L. *et al.* Fruit and vegetable intake and mortality from ischaemic heart disease: results from the European Prospective Investigation into Cancer and Nutrition (EPIC)-Heart study. *Eur. Heart J.* **32**, 1235–1243 (2011).
9. Cawthon, R. M. Telomere length measurement by a novel monochrome multiplex quantitative PCR method. *Nucleic Acids Res.* **37**, e21–e21 (2009).
10. Verhoeven, J. E. *et al.* Major depressive disorder and accelerated cellular aging: results from a large psychiatric cohort study. *Mol. Psychiatry* **19**, 895–901 (2014).
11. Zhao, S. & Fernald, R. D. Comprehensive Algorithm for Quantitative Real-Time Polymerase Chain Reaction. *J. Comput. Biol.* **12**, 1047–1064 (2005).
12. Ma, Q. *et al.* MAGI3 negatively regulates Wnt/beta-catenin signaling and suppresses malignant phenotypes of glioma cells. *Oncotarget* **6**, 35851–65 (2015).
13. Ma, Q. *et al.* MAGI3 Suppresses Glioma Cell Proliferation via Upregulation of PTEN Expression. *Biomed. Environ. Sci.* **28**, 502–9 (2015).
14. Dell'Angelica, E. C., Mullins, C. & Bonifacino, J. S. AP-4, a novel protein complex related to clathrin adaptors. *J. Biol. Chem.* **274**, 7278–7285 (1999).
15. Hirst, J., Bright, N. A., Rous, B. & Robinson, M. S. Characterization of a fourth adaptor-related protein complex. *Mol. Biol. Cell* **10**, 2787–2802 (1999).
16. Bauer, P. *et al.* Mutation in the AP4B1 gene cause hereditary spastic paraplegia type 47 (SPG47). *Neurogenetics* **13**, 73–76 (2012).
17. Barber, E. K., Dasgupta, J. D., Schlossman, S. F., Trevillyan, J. M. & Rudd, C. E. The CD4 and CD8 antigens are coupled to a protein-tyrosine kinase (p56lck) that phosphorylates the CD3 complex. *Proc. Natl. Acad. Sci. U. S. A.* **86**, 3277–3281 (1989).
18. Iwashima, M., Irving, B. A., van Oers, N. S., Chan, A. C. & Weiss, A. Sequential interactions of the TCR with two distinct cytoplasmic tyrosine kinases. *Science* **263**, 1136–1139 (1994).
19. Sturm, R. A., Cassady, J. L., Das, G., Romo, A. & Evans, G. A. Chromosomal structure and expression of the human OTF1 locus encoding the Oct-1 protein. *Genomics* **16**, 333–341 (1993).

20. Segil, N., Roberts, S. B. & Heintz, N. Mitotic phosphorylation of the Oct-1 homeodomain and regulation of Oct-1 DNA binding activity. *Science* **254**, 1814–1816 (1991).
21. Roberts, S. B., Segil, N. & Heintz, N. Differential phosphorylation of the transcription factor Oct1 during the cell cycle. *Science* **253**, 1022–1026 (1991).
22. Schild-Poulter, C., Shih, A., Yarymowich, N. C. & Hache, R. J. G. Down-regulation of histone H2B by DNA-dependent protein kinase in response to DNA damage through modulation of octamer transcription factor 1. *Cancer Res.* **63**, 7197–7205 (2003).
23. Wysocka, J. & Herr, W. The herpes simplex virus VP16-induced complex: the makings of a regulatory switch. *Trends Biochem. Sci.* **28**, 294–304 (2003).
24. Lupo, B. & Trusolino, L. Inhibition of poly(ADP-ribosyl)ation in cancer: Old and new paradigms revisited. *Biochim. Biophys. Acta - Rev. Cancer* **1846**, 201–215 (2014).
25. Arnoult, N. & Karlseder, J. Complex interactions between the DNA-damage response and mammalian telomeres. *Nat. Struct. Mol. Biol* **22**, 859–866 (2015).
26. Déjardin, J. & Kingston, R. E. Purification of Proteins Associated with Specific Genomic Loci. *Cell* **136**, 175–186 (2009).
27. Liang, Y. *et al.* Association of ACYP2 and TSPYL6 Genetic Polymorphisms with Risk of Ischemic Stroke in Han Chinese Population. *Mol. Neurobiol.* **54**, 5988–5995 (2017).
28. Liu, M. *et al.* Association between single nucleotide polymorphisms in the TSPYL6 gene and breast cancer susceptibility in the Han Chinese population. *Oncotarget* **7**, 54771–54781 (2016).
29. Boulay, J. L., Dennefeld, C. & Alberga, A. The Drosophila developmental gene snail encodes a protein with nucleic acid binding fingers. *Nature* **330**, 395–398 (1987).
30. Hay, R. T. SUMO: A History of Modification. *Mol. Cell* **18**, 1–12 (2005).
31. Jones, a. M. *et al.* TERC polymorphisms are associated both with susceptibility to colorectal cancer and with longer telomeres. *Gut* **61**, 248–254 (2012).
32. Lührig, S. *et al.* Lrrc34, a novel nucleolar protein, interacts with npm1 and ncl and has an impact on pluripotent stem cells. *Stem Cells Dev.* **23**, 2862–74 (2014).
33. Fingerlin, T. E. *et al.* Genome-wide association study identifies multiple susceptibility loci for pulmonary fibrosis. *Nat. Genet.* **45**, 613–620 (2013).
34. Chow, A., Hao, Y. & Yang, X. Molecular characterization of human homologs of yeast MOB1. *Int. J. cancer* **126**, 2079–2089 (2010).
35. Lai, Z.-C. *et al.* Control of cell proliferation and apoptosis by mob as tumor suppressor, mats. *Cell* **120**, 675–685 (2005).
36. Kerjan, G. *et al.* Mice lacking doublecortin and doublecortin-like kinase 2 display altered hippocampal neuronal maturation and spontaneous seizures. *Proc. Natl. Acad. Sci. U. S. A.* **106**, 6766–6771 (2009).
37. Kiss, T., Fayet-Lebaron, E. & Jády, B. E. Box H/ACA Small Ribonucleoproteins. *Mol. Cell* **37**, 597–606 (2010).
38. Kwak, J. E., Wang, L., Ballantyne, S., Kimble, J. & Wickens, M. Mammalian GLD-2 homologs are poly(A) polymerases. *Proc. Natl. Acad. Sci. U. S. A.* **101**, 4407–4412 (2004).
39. Glahder, J. A. & Norrild, B. Involvement of hGLD-2 in cytoplasmic polyadenylation of human p53 mRNA. *APMIS* **119**, 769–775 (2011).
40. Wyman, S. K. *et al.* Post-transcriptional generation of miRNA variants by multiple nucleotidyl transferases contributes to miRNA transcriptome complexity. *Genome Res.* **21**, 1450–1461 (2011).

41. Schmidt, C. K. *et al.* Systematic E2 screening reveals a UBE2D-RNF138-CtIP axis promoting DNA repair. *Nat. Cell Biol.* **17**, 1458–1470 (2015).
42. Lehner, B. *et al.* Analysis of a high-throughput yeast two-hybrid system and its use to predict the function of intracellular proteins encoded within the human MHC class III region. *Genomics* **83**, 153–167 (2004).
43. Tang, W., Kannan, R., Blanchette, M. & Baumann, P. Telomerase RNA biogenesis involves sequential binding by Sm and Lsm complexes. *Nature* **484**, 260–264 (2012).
44. Kim, M. K. *et al.* Regulation of telomeric repeat binding factor 1 binding to telomeres by casein kinase 2-mediated phosphorylation. *J. Biol. Chem.* **283**, 14144–14152 (2008).
45. Baumann, P. Pot1, the Putative Telomere End-Binding Protein in Fission Yeast and Humans. *Science (80-.).* **292**, 1171–1175 (2001).
46. Hockemeyer, D. & Collins, K. Control of telomerase action at human telomeres. *Nat. Struct. Mol. Biol.* **22**, 848–852 (2015).
47. Lange, T. De. Shelterin : the protein complex that shapes and safeguards human telomeres. *Genes Dev.* **19**, 2100–2110 (2005).
48. Speedy, H. E. *et al.* Germ line mutations in shelterin complex genes are associated with familial chronic lymphocytic leukemia. *Blood* **128**, 2319–2326 (2016).
49. Shimizu, A. *et al.* A novel giant gene CSMD3 encoding a protein with CUB and sushi multiple domains: a candidate gene for benign adult familial myoclonic epilepsy on human chromosome 8q23.3-q24.1. *Biochem. Biophys. Res. Commun.* **309**, 143–154 (2003).
50. Toomes, C. *et al.* The presence of multiple regions of homozygous deletion at the CSMD1 locus in oral squamous cell carcinoma question the role of CSMD1 in head and neck carcinogenesis. *Genes. Chromosomes Cancer* **37**, 132–140 (2003).
51. Scholnick, S. B. & Richter, T. M. The role of CSMD1 in head and neck carcinogenesis. *Genes, chromosomes & cancer* **38**, 281–283 (2003).
52. Otsuka, M., Mizuno, Y., Yoshida, M., Kagawa, Y. & Ohta, S. Nucleotide sequence of cDNA encoding human cytochrome c oxidase subunit VIc. *Nucleic Acids Res.* **16**, 10916 (1988).
53. Kile, B. T. *et al.* The SOCS box: a tale of destruction and degradation. *Trends Biochem. Sci.* **27**, 235–241 (2002).
54. Chen, L.-Y., Redon, S. & Lingner, J. The human CST complex is a terminator of telomerase activity. *Nature* **488**, 540–544 (2012).
55. Chang, C.-W., Hsu, W.-B., Tsai, J.-J., Tang, C.-J. C. & Tang, T. K. CEP295 interacts with microtubules and is required for centriole elongation. *J. Cell Sci.* **129**, 2501–2513 (2016).
56. Wu, X. *et al.* ATM phosphorylation of Nijmegen breakage syndrome protein is required in a DNA damage response. *Nature* **405**, 477 (2000).
57. Banin, S. *et al.* Enhanced Phosphorylation of p53 by ATM in Response to DNA Damage. *Science (80-.).* **281**, 1674 LP – 1677 (1998).
58. Fan, J. *et al.* Tetrameric Acetyl-CoA Acetyltransferase 1 Is Important for Tumor Growth. *Mol. Cell* **64**, 859–874 (2016).
59. Fukao, T. *et al.* Molecular cloning and sequence of the complementary DNA encoding human mitochondrial acetoacetyl-coenzyme A thiolase and study of the variant enzymes in cultured fibroblasts from patients with 3-ketothiolase deficiency. *J. Clin. Invest.* **86**, 2086–2092 (1990).

60. Liu, L. *et al.* MCAF1/AM is involved in Sp1-mediated maintenance of cancer-associated telomerase activity. *J. Biol. Chem.* **284**, 5165–5174 (2009).
61. Liu, L. *et al.* MCAF1/AM Is Involved in Sp1-mediated Maintenance of Cancer-associated Telomerase Activity. *J. Biol. Chem.* **284**, 5165–5174 (2009).
62. Jobert, L. *et al.* The Human Base Excision Repair Enzyme SMUG1 Directly Interacts with DKC1 and Contributes to RNA Quality Control. *Mol. Cell* **49**, 339–345 (2013).
63. Lee, J. & Zhou, P. DCAFs, the Missing Link of the CUL4-DDB1 Ubiquitin Ligase. *Mol. Cell* **26**, 775–780 (2007).
64. Gao, J. *et al.* The CUL4-DDB1 ubiquitin ligase complex controls adult and embryonic stem cell differentiation and homeostasis. *Elife* **4**, (2015).
65. Mangino, M. *et al.* DCAF4, a novel gene associated with leucocyte telomere length. *J. Med. Genet.* **52**, 157–162 (2015).
66. Axe, E. L. *et al.* Autophagosome formation from membrane compartments enriched in phosphatidylinositol 3-phosphate and dynamically connected to the endoplasmic reticulum. *J. Cell Biol.* **182**, 685 LP – 701 (2008).
67. Shen, Z., Huang, S., Fang, M. & Wang, X. ENTPD5, an Endoplasmic Reticulum UDPase, Alleviates ER Stress Induced by Protein Overloading in AKT-Activated Cancer Cells. *Cold Spring Harb. Symp. Quant. Biol.* **76**, 217–223 (2011).
68. Fang, M. *et al.* The ER UDPase ENTPD5 Promotes Protein N-Glycosylation, the Warburg Effect, and Proliferation in the PTEN Pathway. *Cell* **143**, 711–724 (2010).
69. Heeringa, S. F. *et al.* COQ6 mutations in human patients produce nephrotic syndrome with sensorineural deafness. *J. Clin. Invest.* **121**, 2013–2024 (2011).
70. Tsang, W. Y. *et al.* CP110 Cooperates with Two Calcium-binding Proteins to Regulate Cytokinesis and Genome Stability. *Mol. Biol. Cell* **17**, 3423–3434 (2006).
71. Hayashi, R., Goto, Y., Ikeda, R., Yokoyama, K. K. & Yoshida, K. CDCA4 is an E2F transcription factor family-induced nuclear factor that regulates E2F-dependent transcriptional activation and cell proliferation. *J. Biol. Chem.* **281**, 35633–35648 (2006).
72. Kranz, T. M. *et al.* The chromosome 15q14 locus for bipolar disorder and schizophrenia: is C15orf53 a major candidate gene? *J. Psychiatr. Res.* **46**, 1414–1420 (2012).
73. Ebinu, J. O. *et al.* RasGRP links T-cell receptor signaling to Ras. *Blood* **95**, 3199–3203 (2000).
74. Roose, J. P., Mollenauer, M., Gupta, V. A., Stone, J. & Weiss, A. A diacylglycerol-protein kinase C-RasGRP1 pathway directs Ras activation upon antigen receptor stimulation of T cells. *Mol. Cell. Biol.* **25**, 4426–4441 (2005).
75. van der Velden, L. M. *et al.* Heteromeric interactions required for abundance and subcellular localization of human CDC50 proteins and class 1 P4-ATPases. *J. Biol. Chem.* **285**, 40088–40096 (2010).
76. Paulusma, C. C. & Oude Elferink, R. P. J. The type 4 subfamily of P-type ATPases, putative aminophospholipid translocases with a role in human disease. *Biochim. Biophys. Acta* **1741**, 11–24 (2005).
77. Gao, L. *et al.* Identification of Rare Variants in ATP8B4 as a Risk Factor for Systemic Sclerosis by Whole-Exome Sequencing. *Arthritis Rheumatol.* **68**, 191–200 (2016).
78. Hosford, D. *et al.* Candidate Single-Nucleotide Polymorphisms From a Genomewide Association Study of Alzheimer Disease. *JAMA Neurol.* **65**, 45–53 (2008).
79. Palfreyman, M. T. & Jorgensen, E. M. Unc13 Aligns SNAREs and Superprimes Synaptic

Vesicles. *Neuron* **95**, 473–475 (2017).
80. McRory, J. E. *et al.* Molecular and functional characterization of a family of rat brain T-type calcium channels. *J. Biol. Chem.* **276**, 3999–4011 (2001).
81. Cribbs, L. L. *et al.* Cloning and characterization of alpha1H from human heart, a member of the T-type Ca2+ channel gene family. *Circ. Res.* **83**, 103–109 (1998).
82. Daniil, G. *et al.* CACNA1H Mutations Are Associated With Different Forms of Primary Aldosteronism. *EBioMedicine* **13**, 225–236 (2016).
83. Vitko, I. *et al.* Functional Characterization and Neuronal Modeling of the Effects of Childhood Absence Epilepsy Variants of CACNA1H, a T-Type Calcium Channel. *J. Neurosci.* **25**, 4844–4855 (2005).
84. Van Steensel, B., Smogorzewska, A. & De Lange, T. TRF2 protects human telomeres from end-to-end fusions. *Cell* **92**, 401–413 (1998).
85. Tian, Y. *et al.* C. elegans Screen Identifies Autophagy Genes Specific to Multicellular Organisms. *Cell* **141**, 1042–1055 (2010).
86. Smogorzewska, A. *et al.* Control of human telomere length by TRF1 and TRF2. *Mol. Cell. Biol.* **20**, 1659–68 (2000).
87. Inano, S. *et al.* RFWD3-Mediated Ubiquitination Promotes Timely Removal of Both RPA and RAD51 from DNA Damage Sites to Facilitate Homologous Recombination. *Mol. Cell* **66**, 622-634.e8 (2017).
88. Fu, X. *et al.* RFWD3-Mdm2 ubiquitin ligase complex positively regulates p53 stability in response to DNA damage. *Proc. Natl. Acad. Sci. U. S. A.* **107**, 4579–4584 (2010).
89. Schilders, G., Raijmakers, R., Raats, J. M. H. & Pruijn, G. J. M. MPP6 is an exosome-associated RNA-binding protein involved in 5.8S rRNA maturation. *Nucleic Acids Res.* **33**, 6795–6804 (2005).
90. Lehner, B. & Sanderson, C. M. A protein interaction framework for human mRNA degradation. *Genome Res.* **14**, 1315–1323 (2004).
91. Moon, D. H. *et al.* Poly(A)-specific ribonuclease (PARN) mediates 3’-end maturation of the telomerase RNA component. *Nat. Genet.* **47**, 1482–1488 (2015).
92. Mutahir, Z. *et al.* Thymidine kinase 1 regulatory fine-tuning through tetramer formation. *FEBS J.* **280**, 1531–1541 (2013).
93. Shintani, M., Urano, M., Takakuwa, Y., Kuroda, M. & Kamoshida, S. Immunohistochemical characterization of pyrimidine synthetic enzymes, thymidine kinase-1 and thymidylate synthase, in various types of cancer. *Oncol. Rep.* **23**, 1345–1350 (2010).
94. Anderson, D. D., Quintero, C. M. & Stover, P. J. Identification of a de novo thymidylate biosynthesis pathway in mammalian mitochondria. *Proc. Natl. Acad. Sci.* **108**, 15163 LP – 15168 (2011).
95. Tempel, W. *et al.* Nicotinamide riboside kinase structures reveal new pathways to NAD+. *PLoS Biol.* **5**, e263 (2007).
96. Han, Z. G. *et al.* Molecular cloning of six novel Krüppel-like zinc finger genes from hematopoietic cells and identification of a novel transregulatory domain KRNB. *J. Biol. Chem.* **274**, 35741–8 (1999).
97. Kotenko, S. V *et al.* IFN-lambdas mediate antiviral protection through a distinct class II cytokine receptor complex. *Nat. Immunol.* **4**, 69–77 (2003).
98. Prosser, H. M. *et al.* Prokineticin receptor 2 (Prokr2) is essential for the regulation of circadian behavior by the suprachiasmatic nuclei. *Proc. Natl. Acad. Sci.* **104**, 648 LP – 653 (2007).

99. Dodé, C. & Rondard, P. PROK2/PROKR2 Signaling and Kallmann Syndrome. *Front. Endocrinol. (Lausanne).* **4**, 19 (2013).
100. Zhu, L. *et al.* Inhibition of cell proliferation by p107, a relative of the retinoblastoma protein. *Genes Dev.* **7**, 1111–1125 (1993).
101. Franzolin, E. *et al.* The deoxynucleotide triphosphohydrolase SAMHD1 is a major regulator of DNA precursor pools in mammalian cells. *Proc. Natl. Acad. Sci. U. S. A.* **110**, 14272–14277 (2013).
102. Ryoo, J. *et al.* The ribonuclease activity of SAMHD1 is required for HIV-1 restriction. *Nat. Med.* **20**, 936–941 (2014).
103. Laguette, N. *et al.* SAMHD1 is the dendritic- and myeloid-cell-specific HIV-1 restriction factor counteracted by Vpx. *Nature* **474**, 654–657 (2011).
104. Margalef, P. *et al.* Stabilization of Reversed Replication Forks by Telomerase Drives Telomere Catastrophe. *Cell* **172**, 439-453.e14 (2018).
105. Ballew, B. J. *et al.* A recessive founder mutation in regulator of telomere elongation helicase 1, RTEL1, underlies severe immunodeficiency and features of Hoyeraal Hreidarsson syndrome. *PLoS Genet.* **9**, e1003695 (2013).
106. Stuart, B. D. *et al.* Exome sequencing links mutations in PARN and RTEL1 with familial pulmonary fibrosis and telomere shortening. *Nat. Genet.* **47**, 512 (2015).
107. Zhang, Y. *et al.* Overexpression of SCLIP promotes growth and motility in glioblastoma cells. *Cancer Biol. Ther.* **16**, 97–105 (2015).
108. You, R. *et al.* Apoptosis of dendritic cells induced by decoy receptor 3 (DcR3). **111**, 1480–1489 (2019).
109. Pitti, R. M. *et al.* Genomic amplification of a decoy receptor for Fas ligand in lung and colon cancer. *Nature* **396**, 699–703 (1998).
110. Yang, C.-R. *et al.* Soluble decoy receptor 3 induces angiogenesis by neutralization of TL1A, a cytokine belonging to tumor necrosis factor superfamily and exhibiting angiostatic action. *Cancer Res.* **64**, 1122–1129 (2004).
111. Chevrier, S. & Corcoran, L. M. BTB-ZF transcription factors, a growing family of regulators of early and late B-cell development. *Immunol. Cell Biol.* **92**, 481–8 (2014).
112. Chen, W.-Y. *et al.* Inhibition of the androgen receptor induces a novel tumor promoter, ZBTB46, for prostate cancer metastasis. *Oncogene* **36**, 6213 (2017).
113. Li, J. S. Z. *et al.* TZAP: A telomere-associated protein involved in telomere length control. *Science (80-.).* **355**, 638–641 (2017).
114. Jahn, A. *et al.* ZBTB48 is both a vertebrate telomere-binding protein and a transcriptional activator. *EMBO Rep.* **18**, 929–946 (2017).
115. Adamson, B., Smogorzewska, A., Sigoillot, F. D., King, R. W. & Elledge, S. J. A genome-wide homologous recombination screen identifies the RNA-binding protein RBMX as a component of the DNA-damage response. *Nat. Cell Biol.* **14**, 318–328 (2012).
